# MafA Expression Preserves Immune Homeostasis in Human and Mouse Islets

**DOI:** 10.3390/genes9120644

**Published:** 2018-12-18

**Authors:** Tania Singh, Luis Sarmiento, Cheng Luan, Rashmi B. Prasad, Jenny Johansson, Luis R. Cataldo, Erik Renström, Shamit Soneji, Corrado Cilio, Isabella Artner

**Affiliations:** 1 Stem Cell Center, Lund University, 22184 Lund, Sweden; tania.singh@med.lu.se (T.S.); jenny.kb.johansson@gmail.com (J.J.); rodrigo.cataldo_buscunan@med.lu.se (L.R.C.); shamit.soneji@med.lu.se (S.S.); 2 Lund University Diabetes Centre, 22184 Lund, Sweden; luis.sarmiento-perez@med.lu.se (L.S.); cheng.luan@med.lu.se (C.L.); rashmi.prasad@med.lu.se (R.B.P.); erik.renstrom@med.lu.se (E.R.); corrado.cilio@med.lu.se (C.C.)

**Keywords:** islet of Langerhans, MafA transcription factor, islet inflammatory microenvironment, interferons, interferon-induced genes

## Abstract

Type 1 (T1D) and type 2 (T2D) diabetes are triggered by a combination of environmental and/or genetic factors. Maf transcription factors regulate pancreatic beta (β)-cell function, and have also been implicated in the regulation of immunomodulatory cytokines like interferon-β (IFNβ1). In this study, we assessed *MAFA* and *MAFB* co-expression with pro-inflammatory cytokine signaling genes in RNA-seq data from human pancreatic islets. Interestingly, *MAFA* expression was strongly negatively correlated with cytokine-induced signaling (such as *IFNAR1*, *DDX58*) and T1D susceptibility genes (*IFIH1*), whereas correlation of these genes with *MAFB* was weaker. In order to evaluate if the loss of MafA altered the immune status of islets, *MafA* deficient mouse islets (*MafA^−/−^*) were assessed for inherent anti-viral response and susceptibility to enterovirus infection. *MafA* deficient mouse islets had elevated basal levels of *Ifnβ1*, *Rig1* (*DDX58* in humans), and *Mda5* (*IFIH1*) which resulted in reduced virus propagation in response to coxsackievirus B3 (CVB3) infection. Moreover, an acute knockdown of MafA in β-cell lines also enhanced Rig1 and Mda5 protein levels. Our results suggest that precise regulation of MAFA levels is critical for islet cell-specific cytokine production, which is a critical parameter for the inflammatory status of pancreatic islets.

## 1. Introduction

Investigations of complex disorders such as cancer, diabetes, and infectious diseases have identified a crucial role of the inflammatory microenvironment in driving immune responses against self-antigens [1,2]. Aberrant activation of the immune system can result in auto-inflammatory and/or autoimmune abnormalities as observed in the pathogenesis of Type 1 diabetes (T1D), but also during inflammatory processes in Type 2 diabetes (T2D) pancreata. Human islets express more than 50% of the known T1D susceptible genes [3], reflecting the importance of β cells, at least in part, for initiating or preventing T1D pathophysiology. One of the crucial factors in the early phase of T1D development is local cytokine signaling by interferons (IFNs) [2,4,5,6,7,8]. IFNs have strong anti-viral and immunomodulatory properties, as IFN signaling regulates the expression of target genes (>2000) known as IFN-induced/stimulated/regulated genes [9,10,11] including *PRKR*, *MYD88*, *TRIM25*, *HIF1A*, *IFIT2*, *IFIH1*, and *DDX58*, which participate in diverse downstream signaling pathways releasing cytokines which affect the overall immune homeostasis within the cell. The IFN signaling network plays a central role in mediating communication between β and immune cells. In a healthy cell, expression of these cytokines is tightly regulated and maintained at low levels, with enhanced expression being observed only as a part of protective cell defense mechanisms against infections or other stress stimuli [12,13,14,15]. Uncontrolled production of cytokines specifically belonging to class type 1 IFNs (IFN-1s) may result in the initiation of destructive autoimmune reactions, now classified as interferonopathies [16]. Hence, it is vital to identify local β-cell genetic factors that may disturb the cellular immune homeostasis by regulating IFN expression in healthy cells.

A previous report has suggested that the MAFB transcription factor plays a pivotal role in the transcriptional control of IFN-1 signaling [17] by proposing that MAFB disrupts formation of the IRF3 enhanceosome complex necessary for *IFNβ1* transcription, thus preventing high levels of *Ifnβ1* transcription. During an acute phase of viral infection, MAFB expression reportedly decreases which allows for IRF3-mediated activation of *IFNβ1* transcription enhancing a pro-inflammatory status. However, if high MAFB protein levels were to be maintained, *IFNβ1* transcription would not be sufficient resulting in an increased susceptibility to viral infection [17,18]. Previous publications have shown that human β cells express both MAFA and MAFB [17,19] and that MAFA expression levels are reduced upon β-cell dysfunction and oxidative stress [20]. Until now, the role of the β-cell-specific insulin activator MAFA, which is closely related to MAFB [21,22,23,24,25] in controlling the expression of IFN-1s, has not been evaluated. MAFA polymorphisms have been identified in T1D patients [26] indicating that changes in the expression of MAFA levels may act as an important T1D susceptibility factor by regulating the islet inflammatory microenvironment.

Our results show that *MAFA* expression is significantly negatively correlated with pro-inflammatory anti-viral response candidate genes in human islets such as *IFIH1* (*Mda5*), *DDX58* (*Rig1*), *TRIM25*, *IFNAR1*, and *IFNAR2*, which are generally induced upon virus sensing as well as through other immune triggers that aggravate IFN expression. Immunohistochemical analysis of T2D pancreata, which have low MAFA expression in islets, confirmed an upregulation of DDX58, IFNAR1, and IFIH1 further supporting a negative correlation between MAFA and interferon-induced signaling genes. To investigate if these correlations were caused by the reduction of MAFA expression, we evaluated *Ifnβ1* as well as IFN-1-induced (IFI) gene expression in *MafA* deficient mouse islets. *MafA* deficient islets showed enhanced expression of both *Ifnβ1* and *Mda5* prior to and post CVB3 enterovirus infection, resulting in reduced virus propagation. Moreover, protein expression of Mda5 and Rig1 was significantly enhanced upon acute knockdown of MafA in a β-cell line. Our human islet gene correlation and mouse studies suggest a critical function of *MAFA* in the regulation of IFN expression which, if left uncontrolled, may lead to the development of autoreactive immune responses against β cells.

## 2. Materials and Methods

### 2.1. MAFA Co-Expression Correlations in RNA-Sequencing Data from Human Pancreatic Islets

Human islets from 191 cadaver donors of European ancestry were provided by the Nordic Islet Transplantation Program Uppsala under full ethical clearance (Uppsala Regional Ethics Board, Pro00001754) and the donor families written informed consent and processed for RNA-sequencing (RNA-seq) for Gene Expression Omnibus (GEO) accession code GSE50398 and GSE108072. RNA extraction, quality control, and sequencing were performed as described before [27,28]. Briefly, total RNA was isolated with the All Prep DNA/RNA Mini Kit according to the manufacturer’s instructions (Qiagen, Hilden, Germany). RNA quality and concentration were measured using an Agilent 2100 bioanalyzer and a NanoDrop ND-1000 (NanoDrop Technologies, Thermo Fisher Scientific, Waltham, MA, USA). RNA samples were prepared using the TruSeq RNA Sample Preparation Kit (Illumina, San Diego, CA, USA) and sequencing was performed on the Hi-Seq2000 platform. RNA-seq data were processed as previously described [27,28]. Briefly, raw counts were normalized using trimmed mean of M-values and log_2_-transformed correlation coefficients. Spearman correlations (R) were calculated to assess correlation of *MAFA* and *MAFB* expression with genes expressed in the human pancreatic islets using R language programming.

### 2.2. Statistical Analysis on *MAF* Co-Expression Correlations

To determine whether the expression of subsets of genes belonging to IFN-1/IFN immune response/IFN-induced/anti-viral/cytokine signaling from the PathCards pathway unification database [29] was correlated with *MAFA* or *MAFB* expression, we compared these gene sets to all expression correlation coefficients (21,806) using a Kolmogorov–Smirnov test, the null hypothesis being that the correlation values from the subset under test and total correlations could be drawn from the same distribution; for example, the expression correlation of *T1D* genes to *MAFA* versus the correlations from all genes to *MAFA*. Those with a Bonferroni corrected *p*-value < 0.01 were considered significantly different. These biases were visualized using density plots using the density function in the R language, where the total correlations were shown in black and the subsets were shown as indicated.

### 2.3. Animals

*MafA* deficient (*MafA^−/−^*) animals were generated by crossing *MafA* floxed [22] with *Sox2*-Cre [30] transgenic animals. All experimental procedures were approved by the Animal Welfare and Ethics committee in the Lund-Malmö region (Jordbruksverket; permit numbers: M 43-13, M 47-12, M 385-12). All experimental procedures were carried out in accordance with approved Swedish national guidelines.

### 2.4. Mouse Islet Isolation

Islets from 2–3-month-old and 6-month-old wild-type (WT) and *MafA^−/−^* mice were collected for RNA isolation and virus infections. For islet isolation, mice were euthanized by cervical dislocation, following V-incision on the lower abdomen to expose the pancreas. The pancreatic bile duct was identified and clamped. A small incision was made at the junction of the main pancreatic duct connecting with the duodenum, to inject a fresh mixture of collagenase P (20 U/mL, Roche, Basel, Switzerland) dissolved in Hanks (Sigma-Aldrich, Merck, St. Louis, MO, USA) until the entire pancreas was filled with the solution. The pancreas was then digested at 37 °C for 10–12 min, followed by tissue disruption through manual shaking and several washes with cold Hanks buffer. Thereafter, islets were picked under an inverted bright-field microscope.

### 2.5. Coxsackievirus B3 Virus Islet Infection Assay

The coxsackievirus B3 (CVB3) strain used in this study was isolated from a patient with aseptic meningitis [31]. Virus stocks were prepared in green monkey kidney (GMK) cells containing Complete Eagle’s Minimum Essential Medium (SVA, Uppsala, Sweden) supplemented with 10% FBS (Biochrom AG, Berlin, Germany). All experiments were performed on 50 hand-picked 2–3-month *WT* and *MafA* mutant islets per well, and cultured in low-attachment plates in 3 mL RPMI (Gibco, Thermo Fisher Scientific, Waltham, MA, USA) containing 5.5 mM glucose (Sigma-Aldrich, Merck, St. Louis, Missouri, USA), supplemented with 10% FBS and 2 mM L-glutamine (Gibco, Thermo Fisher Scientific, Waltham, MA, USA). Free floating islets were infected with a 1000-cell culture infectious dose-50 (CCID50)/0.2 mL of CVB3 strain. Islets were examined each day in a light microscope for virus-induced morphological changes. Virus replication was determined using cytopathic effect (CPE) microtitration assays and expressed as 50% cell culture infective dose (CCID50) per milliliter (mL) according to the Kärber formula [32]. Briefly, 0.2 mL of 10-fold serial dilutions (1:10 to 1:108) of samples of the culture medium collected on day zero, and day three post infection, were added in triplicate to GMK cells cultured in 96-well plates. CPE was read on day five and CCID50 titer was calculated using the Kärber formula. The virus production extent was expressed as the difference between the CCID50 titer at day three and at day zero post infection (samples of culture medium collected directly after infection).

### 2.6. Assessment of Islet Cell Viability

Islets were dissociated using accutase (BD, Bioscience, East Rutherford, NJ, USA) at 37 °C. FBS was added to stop the process after 10 min. The accutase-dissociated islet cells were stained with 7-aminoactinomycin D (7-AAD; Sigma-Aldrich, Merck, St. Louis, MA, USA), which binds to DNA when cell membrane permeability is altered after cell death. Cell suspensions were examined using a CyAn ADP Flow Cytometer with Summit Software v4.3 (Beckman Coulter, Brea, CA, USA). Data were analyzed using the Kaluza software package (Beckman Coulter, Brea, CA, USA).

### 2.7. RNA Extraction from Mouse Islets

RNA from 6-month-old newly isolated WT and mutant islets was extracted using RNeasy mini kit (Qiagen), and treated with RNase free DNaseI (Qiagen). RNA from CVB3-infected islets was extracted using Trizol (Invitrogen, Carlsbad, CA, USA), RNA carrier (AmpTec, Hamburg, Germany), and RNeasy mini kit according to the supplier’s instructions (Qiagen, Hilden, Germany). RNA quality was analyzed with an Agilent 2100 bioanalyzer and samples with RIN (RNA integrity number) higher than seven were used for quantitative PCR. RNA concentrations were measured with a NanoDrop ND-1000 spectrophotometer, and concentrations were equalized for each round of complementary DNA (cDNA) synthesis.

### 2.8. Complementary DNA Synthesis and Quantitative PCR

Reverse transcription was performed using SuperScript III reverse transcriptase (RT) (Invitrogen) according to the manufacturer’s instructions, using at least 100 ng total RNA for cDNA synthesis. All assays were performed with Fast SYBR^®^ Green Master Mix on a StepOnePlusTM Real-Time PCR instrument (Applied Biosystems, Foster City, CA, USA).Primer sequences are listed in Table S1. PCR products were confirmed by agarose and melt curve analysis. For each experiment, RT negative control, real time PCR (q-PCR) negative control (blank), and several housekeeping genes were included. Gene expression data were normalized with delta C_T_ method against the geomean of the internal control genes hypoxanthine-guanine phosphoribosyltransferase (HPRT) and beta actin (β-actin) with the additional two housekeeping genes peptidylprolyl isomerase A (PPIA) and TATA box binding protein (TBP) for virus experiments. Data are represented as mean expression with standard error mean and were analyzed with multiple *t*-test/one-way/two-way analysis of variance (ANOVA) analysis, as indicated in the figure legends.

### 2.9. Cell Culture, Small Interfering RNA Transfection, and Protein Analysis

INS-1 832/13 cells were cultured in RPMI-1640 containing 11.1 mM D-glucose supplemented with 10% fetal bovine serum, 100 U/mL penicillin (Gibco), 100 μg/mL streptomycin (Gibco), 10 mM N-2 hydroxyethylpiperazine-N′-2-ethanesulfonic acid (HEPES), 2 mM glutamine, 1 mM sodium pyruvate, and 50 μM β-mercaptoethanol (Sigma-Aldrich) in a humidified atmosphere containing 95% air and 5% CO_2_ at 37 °C. INS-1 832/13 cells were seeded 1 day prior to transfection. An amount of 30 nM RNA interference oligonucleotides or Negative Control #1 (Ambion, Thermo Fisher Scientific, Waltham, MA, USA) was applied together with Lipofectamine RNAiMAX (Invitrogen, Carlsbad, CA, USA) to silence *MafA*.

Cells were homogenized in ice cold RIPA buffer containing complete protease inhibitor (Roche, asel, Switzerland) 72 h after transfection. Extracted total protein content was measured by Pierce bicinchoninic acid assay protein assay kit (Thermo Fisher Scientific, Waltham, MA, USA), and 10–20 μg of protein was electrophoresed on 4–15% Stain-free sodium dodecyl sulfate–polyacrylamide gel electrophoresis (Bio- Rad, Hercules, CA, USA ). The separated proteins were then transferred onto a polyvinylidene difluoride membrane (Bio- Rad, Hercules, CA, USA), followed by blocking with 5.0% nonfat dry milk in TBST (Tris-buffered saline with Tween 20) (pH 7.4; 0.15M NaCl, 10 mM Tris-HCl, and 0.1% Tween 20) for 1 h at room temperature. The membrane was incubated overnight at 4 °C with anti-Rig1 (1:500, Sigma-Aldrich), Mda5 (1:250, Abcam, Cambridge, UK) and Ifnar1 (1:250, Sigma-Aldrich, Merck, St. Louis, MA, USA) antibodies followed by incubation with anti-rabbit IgG (1:2000, Cell Signaling Technology, Danvers, MA, USA) for 1 h at room temperature. Membranes were developed and analyzed using the Bio-Rad ChemiDocTM MP imaging system and Image Lab^TM^ software (6,0,1: Bio-Rad, Hercules, CA, USA). Normalization was carried out by the total protein blotting.

### 2.10. Immunohistochemistry

Pancreatic biopsies from normoglycemic and T2D donors were provided by the Nordic Network for Clinical Transplantation. Sections were processed for immunohistochemistry [33] Paraffin-embedded human and mouse pancreatic tissue sections were stained with the following antibodies: guinea pig α-insulin (1:1000, DAKO, Glostrup, Denmark), rabbit α-IFNAR1, rabbit α-IFIH1, rabbit α-DDX58 (1:50, Sigma-Aldrich, Merck, St. Louis, MA, USA). Heat-induced epitope retrieval was performed for all stainings. Cy2- and Cy3-conjugated α-guinea pig and α-rabbit secondary antibodies (1:500, Jackson Immuno Research Laboratories, West Grove, PA, USA) were used. Nuclear counter staining was performed using 4′,6-diamidino-2-phenylindole (DAPI, 1:6000, Invitrogen). Immunofluorescence images were captured using a Zeiss 780 confocal microscope using Zen black edition software (10,0,0,910: ZEISS, Oberkochen, Germany). Adobe Photoshop CC (2015.0.0 20150529.r.88 2015/05/29:23:59:59 CL 1024429 x64) and InDesign CC 2015 ( 11,0,1,105 x64: Adobe, San Jose, CA, USA) were used for image processing and preparation of figures.

## 3. Results

### 3.1. Expression of the β-Cell-Specific Transcription Factor *MAFA* Is Strongly Negatively Correlated with Pro-Inflammatory Cytokine-Induced Signaling Networks and T1D Susceptibility Genes

**Figure 1:**
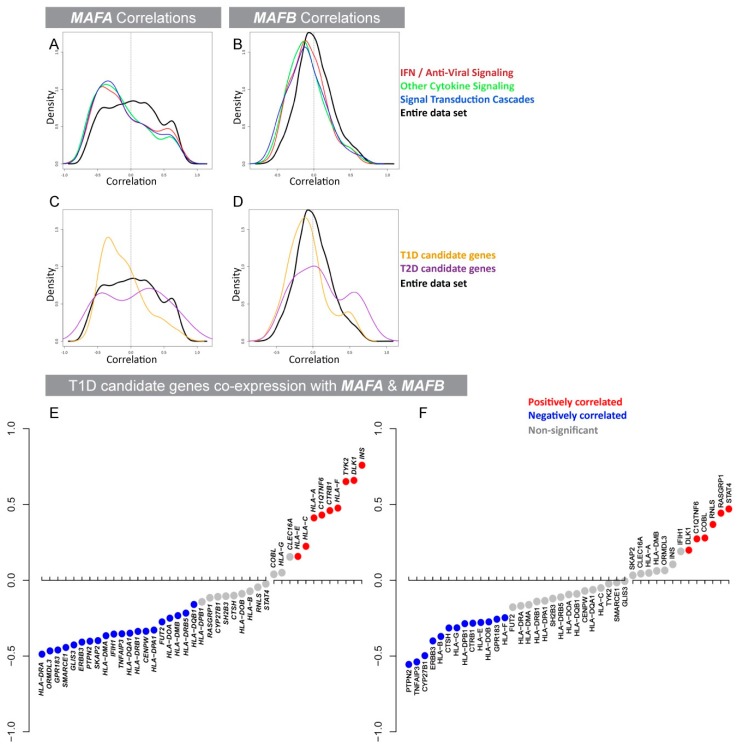
*MAFA* and *MAFB* co-expression comparisons in human islets show a strong correlation between MAFA and cytokine-induced signaling and T1D risk genes. (**A**,**B**) Density correlation distribution plot for interferon, cytokine, and cytokine-induced signaling genes with *MAFA* (*p*-values are *p* = 3.777 × 10^−10^, *p* < 2.2 × 10^−16^, and *p* < 2.2 × 10^−16^, respectively) and MAFB (*p* = 5.457 × 10^−9^, *p* < 2.2 × 10^−16^, and *p* < 2.2 × 10^−16^, respectively) expression. (**C**,**D**) T1D candidate gene (yellow curve) correlations have significantly higher negative associations with *MAFA* (*p* = 7.942 × 10^−5^) than with *MAFB* (*p* = 0.01756) expression. (**C**,**D**) T2D candidate gene (purple curve) correlations with *MAFB* (*p* = 0.008516) and *MAFA* (*p* = 0.4022). (**A**–**D**) Black curve represents the density correlations from the whole transcriptome with *MAFA* and *MAFB* (*n* = 21,806). (**E**–**F**) HLA and islet expressed T1D susceptibility gene expression correlations with (**E**) *MAFA* and (**F**) *MAFB*. *p* < 0.01 was considered significant and was determined using Kolmogorov–Smirnov tests.

MAFA and MAFB are expressed in human β cells and are crucial for maintaining blood glucose levels in a cooperative and sequential manner [21,22,23,24]. MafB expression is vital for β-cell development [21,34,35] and has been implicated in the regulation of IFN-1s in other cell types [17,18]. In contrast, mouse studies identified the importance of MafA in the later stages of development and in adults promoting functional maturation of β cells [23,24,36]. MAFA expression is altered in response to pathological states such as oxidative stress and hyperglycemia in human islets [20,37], however its role in regulating cytokine signaling in the islet has not yet been studied. To establish if *MAFA* and/or *MAFB* expression was correlated with cytokine production in human islets, gene co-expression profiles from human islet RNA-seq were generated and analyzed. For this we selected the entire panel of genes listed under cytokine regulated pathways using keywords such as IFN-1/IFN immune response/IFN-induced/anti-viral/cytokine signaling from the PathCards pathway unification database [29]. This enabled us to generate an unbiased list of genes participating in inflammatory signaling pathways as well as in T1D and T2D susceptibility (Tables S2 and S3). Interestingly, expression of *MAFA* was strongly negatively correlated with pro-inflammatory signaling pathway genes, whereas *MAFB* expression correlations were only weakly associated (Figure 1A,B; Table S2A,C). Moreover, T1D susceptible/risk genes had a strong negative correlation distribution (*p* = 7.942 × 10^−5^) with *MAFA*, whereas *MAFB* was only weakly correlated (*p* = 0.01756, Figure 1C,D; yellow line; Table S3A). In contrast, *MAFA* and *MAFB* co-expression was predominantly positively correlated with T2D susceptibility genes (Figure 1C,D: purple line; Table S3B) supporting the known function of MAF transcription factors in activating glucose sensing and insulin secretion genes [22,24]. To dissect the co-expression correlation data further, all T1D susceptible genes, previously shown to be expressed in islets/β cells, as well as human leukocyte antigen [38] genes were selected and their respective correlation values with *MAFA* (Figure 1E) and *MAFB* (Figure 1F) were assessed. Interestingly, *MAFA* and *MAFB* showed opposing correlations for some T1D risk genes such as *ORMDL3*, *HLA-DMB*, *HLA-E*, *CTRB1*, and *IFIH1*. Overall, our analysis suggests that *MAFA* expression in β cells is critical for establishing a physiologically balanced immune microenvironment in human islets.

### 3.2. Interferon-Induced (IFI) Genes *IFIH1* (*Mda5*) and *DDX58* (*Rig1*) Were Negatively Correlated with MAFA Expression in Human Islets and Protein Expression Was Enhanced in Type 2 Diabetic Islets

**Figure 2:**
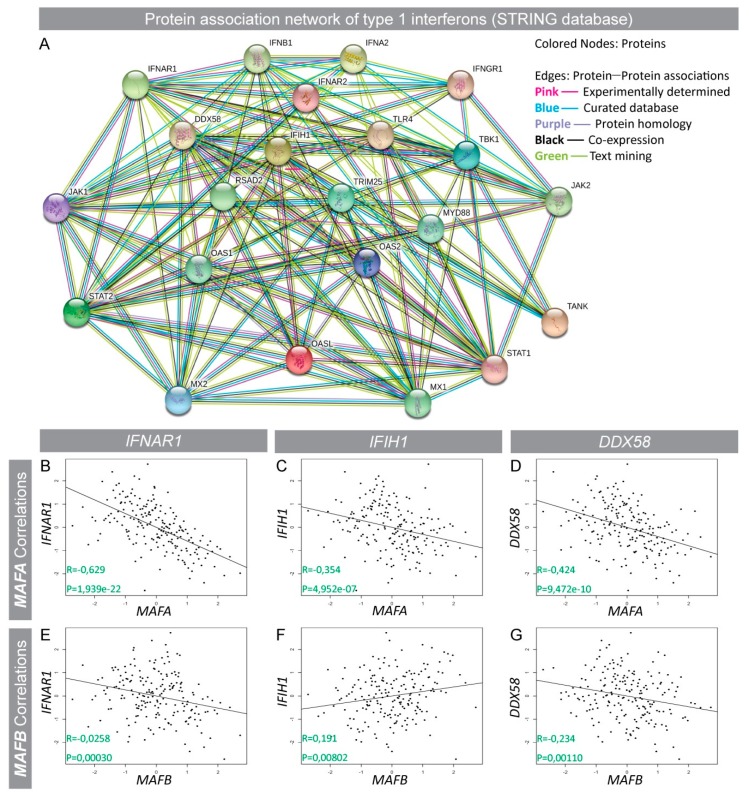
*MAFA* is highly negatively correlated with interferon-induced (*IFI*) genes in human islets. (**A**) Protein–protein association network of *IFN-1*-induced signaling from the STRING database. (**B**–**G**) Individual correlations of (**B**–**D**) MAFA and (**E**–**G**) MAFB expression with *IFI* genes *IFNAR1*, *IFIH1*, and *DDX58* assessed by RNA-seq data analysis of human donor islets (*n* = 195). (**B**–**G**) Spearman correlations (R) and *p*-values are indicated in respective plots.

**Figure 3:**
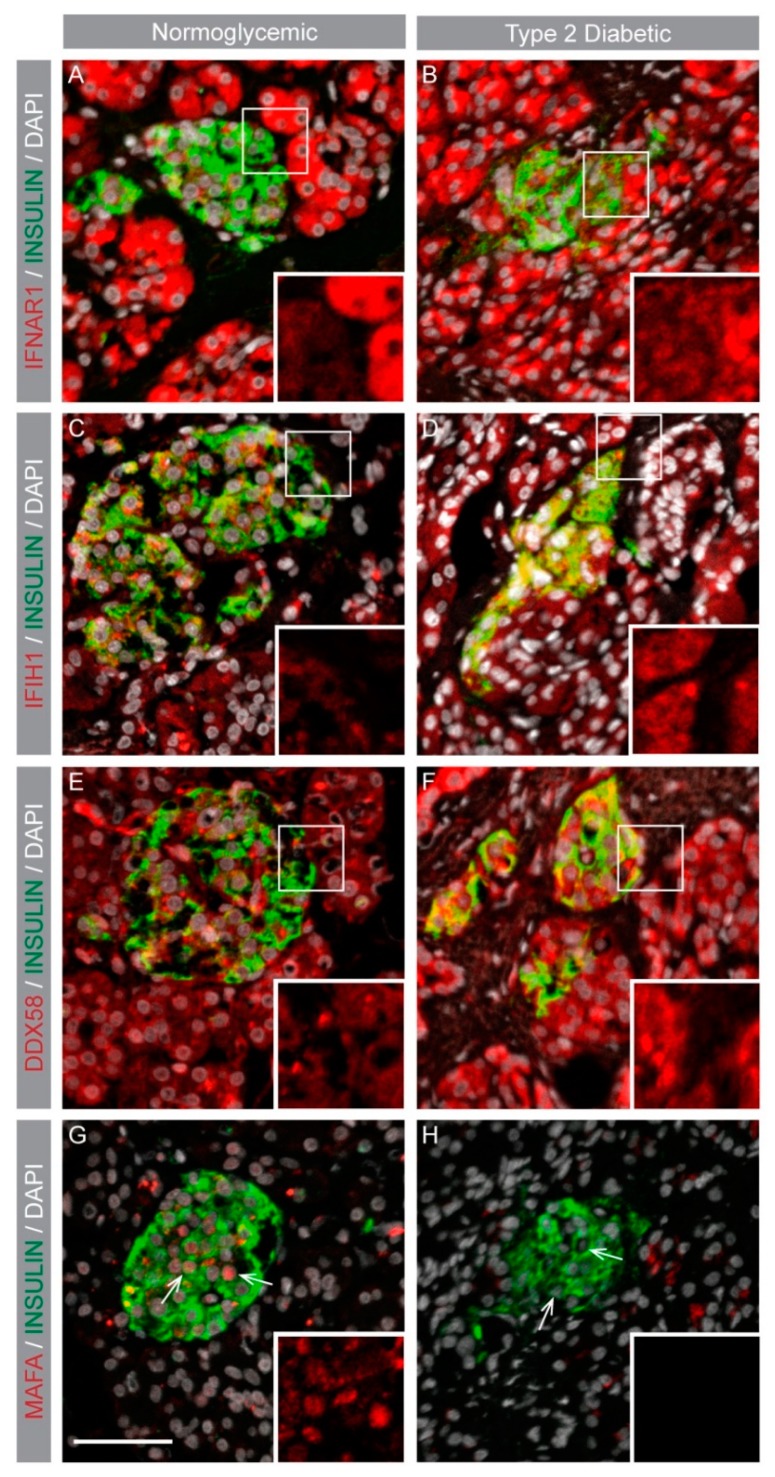
IFI protein expression is enhanced in MAFA-deficient T2D endocrine cells. (**A**–**F**) Normoglycemic and T2D pancreatic sections showing immunohistochemistry stainings for IFNAR1, IFIH1, DDX58 (red) with INSULIN (green) co-expression, and nuclei (grey). Images were captured at 20× magnification and the scale bar is 50 µm. (**A**–**F**) Small white boxes depict area shown in the inserts as magnified portions. (**G**,**H**) White arrows point to nuclei magnified in the inserts.

Human islet *MAFA* gene correlations and comparisons pointed to a significant role of *MAFA* (directly and/or indirectly) in regulating IFN signaling networks, including downstream signaling components like IFN-induced genes. In silico protein association network analysis using the STRING database [39] identified key IFN-1 signaling-induced targets (Figure 2A) which, if dysregulated, may alter the β-cell pro-inflammatory environment. Expression of IFI pro-inflammatory cytokines *IFNα, IFNβ1* as well as anti-viral genes *IFIH1* and *DDX58* was assessed in human islets. *IFN* gene expression was not detected in human islet RNA-seq; however, IFN receptor *IFNAR1*, *IFIH1*, and *DDX58* transcripts were present and showed a strong negative correlation with *MAFA* (Figure 2B–D), whereas these genes had weaker correlations with *MAFB* (Figure 2E–G). Similarly, other IFI genes such as *IFNAR2* and *TRIM25* were also negatively correlated with *MAFA* (Figure S1). Immunohistochemical analysis of pancreatic samples from normoglycemic and T2D donors was performed to assess if IFIH1, DDX58, and IFNAR1 were expressed in islets and if expression was increased in T2D islets which had lower MAFA expression [20]. Expression of IFNAR1, IFIH1, and DDX58 was detected in both exocrine and endocrine pancreas from normoglycemic donors, and expression appeared to be enhanced in T2D endocrine cells (Figure 3B,D,F). As expected, MAFA expression was drastically reduced in T2D compared to control β cells (Figure 3G,H) further supporting the notion that MAFA expression was negatively associated with IFI pro-inflammatory genes.

### 3.3. Acute and Long-Term Loss of MafA Enhances Mda5 and Rig1 Expression

**Figure 4:**
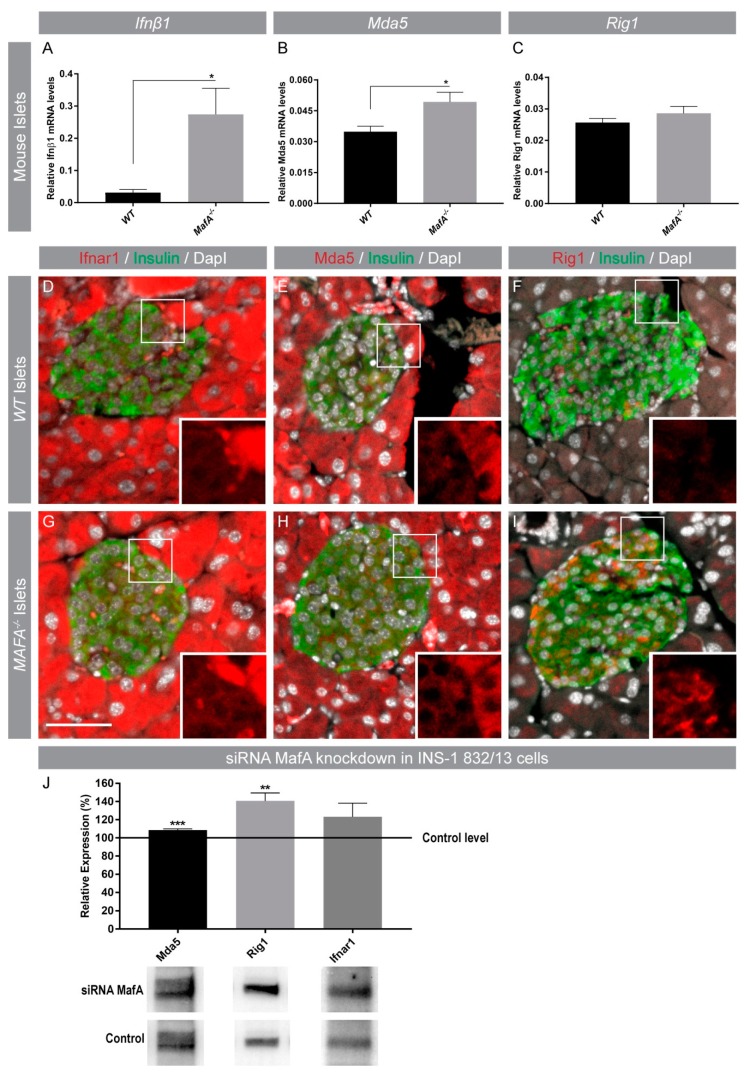
MafA loss induces expression of *IFI* genes. (**A**–**C**) Gene expression analysis of (**A**) *Ifnβ1* and *IFI* genes (**B**) MDA5 and (**C**) Rig1 in MafA deficient mouse islets, sample number (n) is at least 5 or 6 mice per genotype. Data are presented as ± standar error of the mean and were analyzed using Welch’s *t*-test with * *p* < 0.05 as significant. (**D**–**I**) WT and *MafA^−/−^* pancreatic sections showing immunohistochemical stainings for Ifnar1, Mda5, Rig1 (red) with insulin (green) co-expression, and nuclei (grey). Images were captured at 20× magnification and the scale bar is 50 µm. (**D**–**I**) Small white boxes depict area shown in the inserts as magnified portions. (**J**) Western blot analysis from INS-1 832/13 cells treated with small interfering RNA (siRNA) MafA showing relative protein expression (*n* = 4) ** *p* < 0.01, *** *p* < 0.001.

Previous studies have shown that MAFB negatively regulates *IFNβ1* transcription in human cell lines [17], thus we next wanted to investigate if MafA had a similar function in adult mouse β cells which lack MafB expression. Gene expression analysis of mouse *MafA* deficient islets (*MafA^−/−^*) showed that *Ifnβ1* transcription was significantly enhanced in *MafA^−/−^* islets (Figure 4A). Additionally, expression of the *IFI* gene *Mda5* but not *Rig1* was significantly increased (Figure 4B,C) in *MafA^−/−^* islets. Immunohistochemical analysis of WT and *MafA^−/−^* pancreata confirmed that Mda5, Rig1, and Ifnar1 were present in the mouse pancreas and expression appeared to be specifically enhanced in *MafA^−/−^* β cells (Figure 4D–I). To assess if acute loss of MafA had a similar effect on islet cytokine expression, INS-1 832/13 cells were treated with MafA-specific siRNAs. Acute loss of MafA resulted in a significant upregulation of Mda5 and Rig1 protein levels (Figure 4J) suggesting that MafA’s function in maintaining immune homeostasis is conserved in human and mouse β cells.

### 3.4. *MafA* Deficient Mouse Islets Have Reduced Virus Propagation after Coxsackievirus B3 Infection

**Figure 5:**
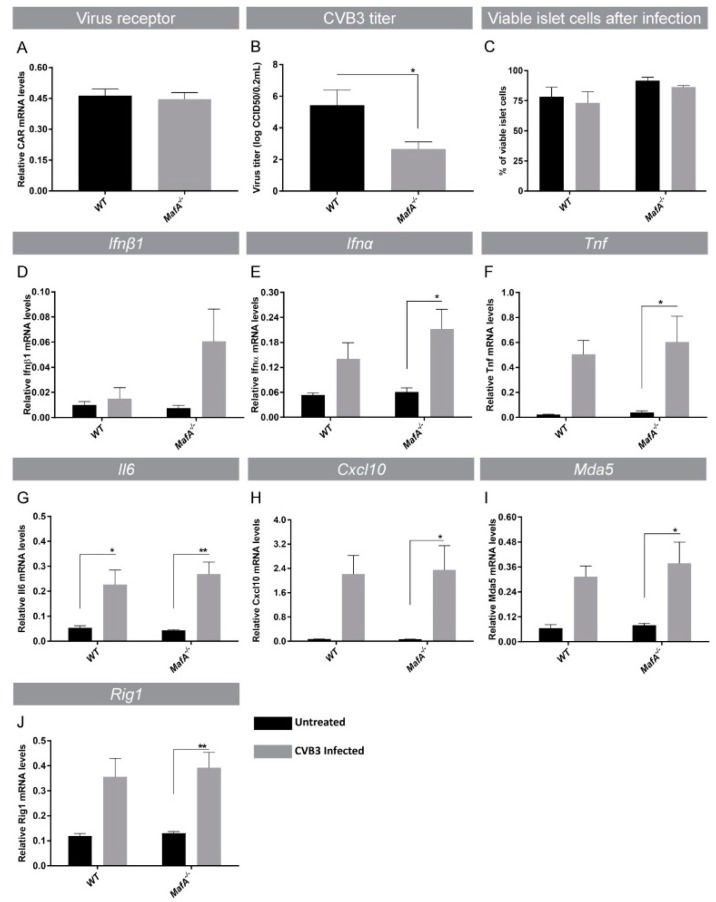
*MafA* deficient mouse islets have an intact anti-viral response. (**A**) CVB3 virus receptor adenovirus receptor (CAR) expression was unaltered in *MafA^−/−^* islets (*n* = 5/6 per genotype). (**B**) CVB3 virus titer was reduced in *MafA^−/−^* islets (*n* ≥ 10 per genotype). (**C**) No changes in the percentage of viable cells were observed between CVB3-infected WT and *MafA^−/−^* islets (*n* = 3/4 per genotype). (**D**–**H**) Cytokines as well as (**I**,**J**) anti-viral response gene expression in WT and *MafA^−/−^* islets after CVB3 infection (*n* ≤ 10 per genotype). (**A**–**J**) Data were presented as ± SEM and analyzed using Welch’s *t*-test (**A**,**B**) and Sidak’s multiple comparison test (**C**–**J**) with * *p* < 0.05; ** *p* < 0.01 as significant.

In virus-infected cells, cumulative actions of IFNβ1, IFIH1, and DDX58 play a crucial role in mediating early host cellular defense mechanisms [9,10,11,15,40,41,42,43,44] to initiate anti-viral immune responses against infections. Previous studies have shown that CVB3 replicates in mouse islets [45] and that mouse islet cells express coxsackievirus and adenovirus receptor (CAR) which was readily detectable and unchanged between WT and *MafA^−/−^* islets (Figure 5A). To evaluate if inherent higher expression of anti-viral response genes also restricted virus propagation, virus titer was assessed in WT and *MafA^−/−^* islets infected with CVB3. The amount of newly synthesized virus particles in the culture supernatant was significantly reduced in *MafA^−/−^* islets in comparison with CVB3-infected WT islets (Figure 5B). Remarkably, CVB3 infection did not alter cell viability in WT and mutant islets (Figure 3C) supporting the notion that mouse islet cells remained alive after CVB3 infection [46]. This suggested that the differences in CVB3 propagation were not a result of changes in CAR expression or viability of islets (Figure 3A,C) after infection. Expression analysis revealed that the infected *MafA^−/−^* islets responded efficiently against CVB3 infection by inducing IFNs (Figure 3D,E) as well as other pro-inflammatory cytokines such as tumor necrosis factor (*Tnf*), interleukin 6 (*Il6*), and C-X-C motif chemokine 10 (*Cxcl10*) (Figure 5F–H), as well as IFN-1 anti-viral genes Mda5 and Rig1 (Figure 5I,J). These data show that *MafA^−/−^* islets are more efficient in clearing CVB3 infections than WT islets, most likely due to enhanced basal expression of IFNs and anti-viral genes. Taken together, these results suggest that the loss of MafA in β cells altered the inflammatory microenvironment by producing an inability to restrict Ifnβ1 expression efficiently.

## 4. Discussion

Immunomodulatory signaling cytokines such as INF-1s orchestrate an intricate network of regulatory pathways that initiate innate and adaptive immune responses against invading pathogens, as a part of host cell defense mechanisms. In a healthy cell, the pro-inflammatory signature is tightly regulated to avoid any inappropriate immune activation, which may lead to the initiation of destructive autoimmune reactions against self-antigens. Enhanced IFN expression has been detected in T1D pancreatic tissue [7,47] as well as in the blood of children prior to clinical onset of T1D [6,48]. Several reports have also linked the development of T1D to IFN-1 therapy for the treatment of chronic diseases such as viral hepatitis and malignant tumors [8,49,50,51]. Moreover, T1D genetic associations with IFN-1 regulation and signaling pathways have been identified by genome wide association studies [52,53,54]. Thus, genetic abnormalities leading to inherent overproduction of IFN/cytokines may enhance the risk of developing interferonopathies and/or autoimmune disorders such as T1D [6,7,47,48] even in the absence of an antigen-mediated trigger. Therefore, in our present study, we evaluated and compared the relation of transcription factors MAFA and MAFB in controlling the immune equilibrium within human islets by regulating cytokine expression and identified a more prominent function of MAFA in maintaining a physiological immune status in the human islets, an observation that was confirmed in mouse *MafA^−/−^* islets.

Human islet *MAFA* and *MAFB* co-expression analysis with genes involved in inflammatory/cytokine-induced signaling pathways showed a stronger negative correlation with *MAFA* than *MAFB.* This pointed toward an intriguing unknown function of MAFA in the regulation of cytokine expression. Therefore, we next investigated if *MAFA* co-expression was correlated with the expression of T1D risk genes. Interestingly, T1D susceptibility genes expressed in human islets were also strongly negatively correlated with *MAFA* in comparison to *MAFB*. In contrast, expression of genes known to be activated by MAFA (insulin) was positively correlated supporting the known role of MAFA as a regulator of β-cell function [24]. Moreover, individual T1D risk genes such as *IFIH1* and *CTRB1* showed opposite correlations to *MAFA* and *MAFB*, indicating different regulatory functions of MAFA and MAFB in human islets. We also showed that the human β-cell-specific gene *DLK1* was significantly positively correlated with both MAFA and MAFB which had also been implicated in the progression of diabetes by impairing β-cell function [55,56]. These correlations from human islet co-expression analyses with *MAFs* highlight their different functions, by identifying distinct target genes in human islets.

We identified a category of genes induced by IFN-1s (IFI) through protein association network analysis (STRING) [39] and assessed if expression of these genes (*IFIH1*, *DDX58*) with anti-viral function was correlated with *MAFA* and *MAFB*. Expression of type 1 IFNs in human islets was not detected, which most likely reflected their extremely low basal gene expression, although the IFN-1 receptor *IFNAR1* which is induced by high levels of IFNs had a strong negative correlation with *MAFA* expression. A similar negative correlation was observed for *IFIH1* and *DDX58*, indicating that the presence of MAFA is required for the repression of IFNs and their downstream target genes. These results indicate that the reduction of MAFA levels in β cells may enhance the production of IFNs and possibly IFI genes which may further increase the production of pro-inflammatory cytokines in a positive feedback loop. A similar relation of MAFB with IFN-β1 was identified in human cell lines, where high MAFB expression inhibited IFN-1 production by the disruption of an enhanceosome complex, necessary for IFN-β transcription [17]. This study also suggested a role for MAFB in restricting unwarranted induction of IFN-1s in healthy cells, critical for preventing the unnecessary activation of the immune system [17]. Statistical analysis indicated a much weaker negative correlation of *MAFB* with *IFNAR1* and *DDX58* expression, whereas *IFIH1* expression was even positively correlated with MAFB. Our gene correlation assessment suggests a critical immune regulatory function of MAFA by controlling *IFN* and *IFI* gene signature in human islets.

IFNs and intracellular viral recognition helicases *Mda5* (*IFIH1*) and *Rig1* (*DDX58*) are critical components of the cellular anti-viral response cascade, which mediates induction of IFN expression in a regulated manner [57]. However, overexpression of IFN-1 signaling components (through Mda5) may contribute to islet autoimmunity [54] as both Ifnβ1 and Mda5 overexpression have been implicated in pro-inflammatory responses [40]. Previous studies have shown that Mda5 has a dual role in cellular immune responses, because high Mda5 expression levels resulted in decreased susceptibility to viral infection, but also induced a chronic type 1 interferon signature which accelerated autoimmunity [40,58]. Moreover, a majority of polymorphisms in this gene were associated with elevated Mda5 expression and human T1D [40,59,60,61,62,63,64,65] and reduced expression has been shown to induce regulatory immune responses preventing autoimmune diabetes [66]. Our results showed that MafA negatively regulated Ifnβ1 and anti-viral response gene expression in mouse islets as *MafA^−/−^* islets had significantly enhanced basal mRNA levels of *Ifnβ1* and *Mda5* and an acute knockdown of MafA enhanced Mda5 and Rig1 protein levels, which was in accordance with co-expression correlations in human islets and immunohistochemical analysis of type 2 diabetic islets which lacked MAFA expression. As expected, upon CVB3 infection in vitro, both WT and *MafA^−/−^* islets responded by inducing Ifnβ1, Ifnα, Tnf, Il6, Cxcl10, Mda5, and Rig1 mRNA levels, indicating adequate virus recognition and anti-viral response generated through pattern recognition receptors. This finding indicated that both WT and *MafA^−/−^* islets were infected, but *MafA^−/−^* islets eradicated the virus more effectively than WT islets, probably due to their elevated inherent immune response. We also confirmed that the reduction in CVB3 virus titer was not due to apoptosis of islets cells in culture or alterations in CVB3 CAR receptor expression, as no differences among these were detected when compared to WT controls. These results supported the idea that the lower susceptibility of *MafA^−/−^* islets to viral replication was not attributed to differences in virus recognition/entry or their ability to detect viral infection, but due to an inherent elevated immune status which restricted efficient virus propagation.

Previous studies have shown that MafA and MafB expression domains within islets of Langerhans differed significantly between species [19,22,36,67]. MAFB expression in β cells was only detected in primates but developmentally MAFB preceded MAFA expression [22,34,67], whereas MAFA was essential in the later stages of postnatal development and regulated glucose and autonomic nervous system-induced insulin secretion [23,24,33,36]. Human β cells expressed both MAFA and MAFB [17,19], whereas in adult murine islets MafA was specifically detected in β cells and MafB was restricted to α cells [22,34,68]. In this study, we report that *MAFA* co-expression correlations in human islets are much stronger and are associated with a higher number of genes within cytokine signaling and T1D genetic risk spectrum than *MAFB*, indicating that *MAFA* has a unique role in regulating the expression of these immune modulatory genes directly and indirectly through interferon-induced positive feedback loops. Additionally, the loss of β-cell function due to metabolic/oxidative stress also leads to reduced MafA levels [20,37] and may possibly elevate cytokine levels as observed in our human and mouse islet expression analysis, indicating that the loss of MAFA is the primary event leading to a disturbed islet microenvironment. It is highly likely that these cells, in a stressed environment, shed higher amounts of autoantigens as well as secrete pro-inflammatory mediators. Such changes may be recognized by tissue resident innate immune cells, initiating a cascade of destructive adaptive immune responses against β cells leading to the development of T1D and inflammatory processes in T2D. Cultured human islets have relatively high *MAFB* transcript levels, whereas *MAFA* expression was more difficult to detect [19]. This suggests that cultured islets, which are critical for scientific studies, but also commonly used for cell replacement therapy, are dysfunctional and have an altered cytokine expression profile. This is of clinical interest as alterations in cytokine expression may significantly affect the therapeutic success of islet transplantation by triggering graft rejection and poor graft cell survival. Thus, a thorough analysis of MAFA, MAFB, and cytokine expression levels may be a critical indicator of transplantation outcome.

## Supplementary Materials

The following are available online at http://www.mdpi.com/2073-4425/9/12/644/s1, Figure S1: *MAFA* and MAFBA are negatively correlated with IFI genes, Table S1: Quantitative PCR primer sequences, Tables S2 and S3: Gene list from PathCards pathway unification database.

## References

[1] Cheon H., Borden E.C., Stark G.R. (2014). Interferons and their stimulated genes in the tumor microenvironment. Semin. Oncol..

[2] Newby B.N., Mathews C.E. (2017). Type I interferon is a catastrophic feature of the diabetic islet microenvironment. Front. Endocrinol. (Lausanne).

[3] Eizirik D.L., Sammeth M., Bouckenooghe T., Bottu G., Sisino G., Igoillo-Esteve M., Ortis F., Santin I., Colli M.L., Barthson J. (2012). The human pancreatic islet transcriptome: expression of candidate genes for type 1 diabetes and the impact of pro-inflammatory cytokines. PLoS Genet..

[4] Akbar A.N., Lord J.M., Salmon M. (2000). IFN-alpha and IFN-beta: A link between immune memory and chronic inflammation. Immunol. Today.

[5] Alba A., Puertas M.C., Carrillo J., Planas R., Ampudia R., Pastor X., Bosch F., Pujol-Borrell R., Verdaguer J., Vives-Pi M. (2004). IFN beta accelerates autoimmune type 1 diabetes in nonobese diabetic mice and breaks the tolerance to beta cells in nondiabetes-prone mice. J. Immunol..

[6] Ferreira R.C., Guo H., Coulson R.M., Smyth D.J., Pekalski M.L., Burren O.S., Cutler A.J., Doecke J.D., Flint S., McKinney E.F. (2014). A type I interferon transcriptional signature precedes autoimmunity in children genetically at risk for type 1 diabetes. Diabetes.

[7] Huang X., Yuang J., Goddard A., Foulis A., James R.F., Lernmark A., Pujol-Borrell R., Rabinovitch A., Somoza N., Stewart T.A. (1995). Interferon expression in the pancreases of patients with type I diabetes. Diabetes.

[8] Kado S., Miyamoto J., Komatsu N., Iwaki Y., Ozaki H., Taguchi H., Kure M., Sarashina G., Watanabe T., Katsura Y. (2000). Type 1 diabetes mellitus caused by treatment with interferon-beta. Intern. Med..

[9] Schneider W.M., Chevillotte M.D., Rice C.M. (2014). Interferon-stimulated genes: A complex web of host defenses. Annu. Rev. Immunol..

[10] Schoggins J.W., Rice C.M. (2011). Interferon-stimulated genes and their antiviral effector functions. Curr. Opin. Virol..

[11] Takaoka A., Yanai H. (2006). Interferon signalling network in innate defence. Cell. Microbiol..

[12] Belardelli F. (1995). Role of interferons and other cytokines in the regulation of the immune response. APMIS.

[13] Bocci V. (1985). The physiological interferon response. Immunol. Today.

[14] Taniguchi T., Takaoka A. (2001). A weak signal for strong responses: interferon-alpha/beta revisited. Nat. Rev. Mol. Cell Biol..

[15] Taniguchi T., Takaoka A. (2002). The interferon-alpha/beta system in antiviral responses: a multimodal machinery of gene regulation by the IRF family of transcription factors. Curr. Opin. Immunol..

[16] Qaisar N., Jurczyk A., Wang J.P. (2018). Potential role of type I interferon in the pathogenic process leading to type 1 diabetes. Curr. Opin. Endocrinol. Diabetes Obes..

[17] Kim H., Seed B. (2010). The transcription factor MafB antagonizes antiviral responses by blocking recruitment of coactivators to the transcription factor IRF3. Nat. Immunol..

[18] Motohashi H., Igarashi K. (2010). MafB as a type I interferon rheostat. Nat. Immunol..

[19] Dai C., Brissova M., Hang Y., Thompson C., Poffenberger G., Shostak A., Chen Z., Stein R., Powers A.C. (2012). Islet-enriched gene expression and glucose-induced insulin secretion in human and mouse islets. Diabetologia.

[20] Guo S., Dai C., Guo M., Taylor B., Harmon J.S., Sander M., Robertson R.P., Powers A.C., Stein R. (2013). Inactivation of specific beta cell transcription factors in type 2 diabetes. J. Clin. Investig..

[21] Artner I., Bianchi B., Raum J.C., Guo M., Kaneko T., Cordes S., Sieweke M., Stein R. (2007). MafB is required for islet beta cell maturation. Proc. Natl. Acad. Sci. USA.

[22] Artner I., Hang Y., Mazur M., Yamamoto T., Guo M., Lindner J., Magnuson M.A., Stein R. (2010). MafA and MafB regulate genes critical to beta-cells in a unique temporal manner. Diabetes.

[23] Nishimura W., Takahashi S., Yasuda K. (2015). MafA is critical for maintenance of the mature beta cell phenotype in mice. Diabetologia.

[24] Zhang C., Moriguchi T., Kajihara M., Esaki R., Harada A., Shimohata H., Oishi H., Hamada M., Morito N., Hasegawa K. (2005). MafA is a key regulator of glucose-stimulated insulin secretion. Mol. Cell. Biol..

[25] Zhu Y., Liu Q., Zhou Z., Ikeda Y. (2017). PDX1, Neurogenin-3, and MAFA: Critical transcription regulators for beta cell development and regeneration. Stem Cell Res. Ther..

[26] Noso S., Kawabata Y., Babaya N., Hiromine Y., Kawasaki E. (2013). Association study of *MAFA* and *MAFB*, genes related to organ-specific autoimmunity, with susceptibility to type 1 diabetes in japanese and caucasian populations. J. Genet. Syndr. Gene Ther..

[27] Fadista J., Vikman P., Laakso E.O., Mollet I.G., Esguerra J.L., Taneera J., Storm P., Osmark P., Ladenvall C., Prasad R.B. (2014). Global genomic and transcriptomic analysis of human pancreatic islets reveals novel genes influencing glucose metabolism. Proc. Natl. Acad. Sci. USA.

[28] Ottosson-Laakso E., Krus U., Storm P., Prasad R.B., Oskolkov N., Ahlqvist E., Fadista J., Hansson O., Groop L., Vikman P. (2017). Glucose-induced changes in gene expression in human pancreatic islets: Causes or consequences of chronic hyperglycemia. Diabetes.

[29] Belinky F., Nativ N., Stelzer G., Zimmerman S., Iny Stein T., Safran M., Lancet D. (2015). PathCards: multi-source consolidation of human biological pathways. Database.

[30] Hayashi S., Tenzen T., McMahon A.P. (2003). Maternal inheritance of Cre activity in a Sox2Cre deleter strain. Genesis.

[31] Sarmiento L. (2004). Enteroviral Meningitis and Emergence of Rare Enterovirus Types: Cuban Experience.

[32] Lennette E.H. (1969). General Principles Underlying Laboratory Diagnosis of Viral and Rickettsial Infections.

[33] Ganic E., Singh T., Luan C., Fadista J., Johansson J.K., Cyphert Holly A., Bennet H., Storm P., Prost G., Ahlenius H. (2002). MafA-controlled nicotinic receptor expression is essential for insulin secretion and is impaired in patients with type 2 diabetes. Cell Rep..

[34] Artner I., Le Lay J., Hang Y., Elghazi L., Schisler J.C., Henderson E., Sosa-Pineda B., Stein R. (2006). MafB: an activator of the glucagon gene expressed in developing islet alpha- and beta-cells. Diabetes.

[35] Nishimura W., Kondo T., Salameh T., El Khattabi I., Dodge R., Bonner-Weir S., Sharma A. (2006). A switch from MafB to MafA expression accompanies differentiation to pancreatic beta-cells. Dev. Biol..

[36] Hang Y., Yamamoto T., Benninger R.K., Brissova M., Guo M., Bush W., Piston D.W., Powers A.C., Magnuson M., Thurmond D.C. (2014). The MafA transcription factor becomes essential to islet beta-cells soon after birth. Diabetes.

[37] Butler A.E., Robertson R.P., Hernandez R., Matveyenko A.V., Gurlo T., Butler P.C. (2012). Beta cell nuclear musculoaponeurotic fibrosarcoma oncogene family A (MafA) is deficient in type 2 diabetes. Diabetologia.

[38] Stewart T.A., Hultgren B., Huang X., Pitts-Meek S., Hully J., MacLachlan N.J. (1993). Induction of type I diabetes by interferon-alpha in transgenic mice. Science.

[39] Szklarczyk D., Franceschini A., Wyder S., Forslund K., Heller D., Huerta-Cepas J., Simonovic M., Roth A., Santos A., Tsafou K.P. (2015). STRING v10: Protein-protein interaction networks, integrated over the tree of life. Nucleic Acids Res..

[40] Crampton S.P., Deane J.A., Feigenbaum L., Bolland S. (2012). *Ifih1* gene dose effect reveals MDA5-mediated chronic type I IFN gene signature, viral resistance, and accelerated autoimmunity. J. Immunol..

[41] Errett J.S., Suthar M.S., McMillan A., Diamond M.S., Gale M. (2013). The essential, nonredundant roles of RIG-I and MDA5 in detecting and controlling West Nile virus infection. J. Virol..

[42] Kato H., Takeuchi O., Sato S., Yoneyama M., Yamamoto M., Matsui K., Uematsu S., Jung A., Kawai T., Ishii K.J. (2006). Differential roles of MDA5 and RIG-I helicases in the recognition of RNA viruses. Nature.

[43] Gorman J.A., Hundhausen C., Errett J.S., Stone A.E., Allenspach E.J., Ge Y., Arkatkar T., Clough C., Dai X., Khim S. (2017). The A946T variant of the RNA sensor IFIH1 mediates an interferon program that limits viral infection but increases the risk for autoimmunity. Nat. Immunol..

[44] Hultcrantz M., Huhn M.H., Wolf M., Olsson A., Jacobson S., Williams B.R., Korsgren O., Flodstrom-Tullberg M. (2007). Interferons induce an antiviral state in human pancreatic islet cells. Virology.

[45] Drescher K.M., Kono K., Bopegamage S., Carson S.D., Tracy S. (2004). Coxsackievirus B3 infection and type 1 diabetes development in NOD mice: Insulitis determines susceptibility of pancreatic islets to virus infection. Virology.

[46] Kanno T., Kim K., Kono K., Drescher K.M., Chapman N.M., Tracy S. (2006). Group B coxsackievirus diabetogenic phenotype correlates with replication efficiency. J. Virol..

[47] Foulis A.K., Farquharson M.A., Meager A. (1987). Immunoreactive alpha-interferon in insulin-secreting beta cells in type 1 diabetes mellitus. Lancet.

[48] Kallionpaa H., Elo L.L., Laajala E., Mykkanen J., Ricano-Ponce I., Vaarma M., Laajala T.D., Hyoty H., Ilonen J., Veijola R. (2014). Innate immune activity is detected prior to seroconversion in children with HLA-conferred type 1 diabetes susceptibility. Diabetes.

[49] Guerci A.P., Guerci B., Levy-Marchal C., Ongagna J., Ziegler O., Candiloros H., Guerci O., Drouin P. (1994). Onset of insulin-dependent diabetes mellitus after interferon-alfa therapy for hairy cell leukaemia. Lancet.

[50] Oka R., Hiroi N., Shigemitsu R., Sue M., Oshima Y., Yoshida-Hiroi M. (2011). Type 1 diabetes mellitus associated with pegylated interferon-alpha plus ribavirin treatment for chronic hepatitis c: case report and literature review. Clin. Med. Insights Endocrinol. Diabetes.

[51] Uonaga T., Yoshida K., Harada T., Shimodahira M., Nakamura Y. (2012). Case of type 1 diabetes mellitus following interferon beta-1a treatment for multiple sclerosis. Intern. Med..

[52] Concannon P., Rich S.S., Nepom G.T. (2009). Genetics of type 1A diabetes. N. Engl. J. Med..

[53] Todd J.A. (2010). Etiology of type 1 diabetes. Immunity.

[54] Santin I., Eizirik D.L. (2013). Candidate genes for type 1 diabetes modulate pancreatic islet inflammation and beta-cell apoptosis. Diabetes. Obes. Metab..

[55] Kameswaran V., Bramswig N.C., McKenna L.B., Penn M., Schug J., Hand N.J., Chen Y., Choi I., Vourekas A., Won K.J. (2014). Epigenetic regulation of the DLK1-MEG3 microRNA cluster in human type 2 diabetic islets. Cell Metab..

[56] Wallace C., Smyth D.J., Maisuria-Armer M., Walker N.M., Todd J.A., Clayton D.G. (2010). The imprinted DLK1-MEG3 gene region on chromosome 14q32.2 alters susceptibility to type 1 diabetes. Nat. Genet..

[57] Yoneyama M., Onomoto K., Jogi M., Akaboshi T., Fujita T. (2015). Viral RNA detection by RIG-I-like receptors. Curr. Opin. Immunol..

[58] Rice G.I., Del Toro Duany Y., Jenkinson E.M., Forte G.M., Anderson B.H., Ariaudo G., Bader-Meunier B., Baildam E.M., Battini R., Beresford M.W. (2014). Gain-of-function mutations in IFIH1 cause a spectrum of human disease phenotypes associated with upregulated type I interferon signaling. Nat. Genet..

[59] Liu S., Wang H., Jin Y., Podolsky R., Reddy M.V., Pedersen J., Bode B., Reed J., Steed D., Anderson S. (2009). IFIH1 polymorphisms are significantly associated with type 1 diabetes and IFIH1 gene expression in peripheral blood mononuclear cells. Hum. Mol. Genet..

[60] Zurawek M., Fichna M., Fichna P., Skowronska B., Dzikiewicz-Krawczyk A., Januszkiewicz D., Nowak J. (2015). Cumulative effect of IFIH1 variants and increased gene expression associated with type 1 diabetes. Diabetes Res. Clin. Pract..

[61] Winkler C., Lauber C., Adler K., Grallert H., Illig T., Ziegler A.G., Bonifacio E. (2011). An interferon-induced helicase (*IFIH1*) gene polymorphism associates with different rates of progression from autoimmunity to type 1 diabetes. Diabetes.

[62] Robinson T., Kariuki S.N., Franek B.S., Kumabe M., Kumar A.A., Badaracco M., Mikolaitis R.A., Guerrero G., Utset T.O., Drevlow B.E. (2011). Autoimmune disease risk variant of *IFIH1* is associated with increased sensitivity to IFN-alpha and serologic autoimmunity in lupus patients. J. Immunol..

[63] Jermendy A., Szatmari I., Laine A.P., Lukacs K., Horvath K.H., Korner A., Madacsy L., Veijola R., Simell O., Knip M. (2010). The interferon-induced helicase IFIH1 Ala946Thr polymorphism is associated with type 1 diabetes in both the high-incidence Finnish and the medium-incidence Hungarian populations. Diabetologia.

[64] Aminkeng F., Van Autreve J.E., Weets I., Quartier E., Van Schravendijk C., Gorus F.K., Van der Auwera B.J., Belgian Diabetes R. (2009). *IFIH1* gene polymorphisms in type 1 diabetes: genetic association analysis and genotype-phenotype correlation in the Belgian population. Hum. Immunol..

[65] Smyth D.J., Cooper J.D., Bailey R., Field S., Burren O., Smink L.J., Guja C., Ionescu-Tirgoviste C., Widmer B., Dunger D.B. (2006). A genome-wide association study of nonsynonymous SNPs identifies a type 1 diabetes locus in the interferon-induced helicase (*IFIH1*) region. Nat. Genet..

[66] Lincez P.J., Shanina I., Horwitz M.S. (2015). Reduced expression of the MDA5 Gene *IFIH1* prevents autoimmune diabetes. Diabetes.

[67] Conrad E., Dai C., Spaeth J., Guo M., Cyphert H.A., Scoville D., Carroll J., Yu W.M., Goodrich L.V., Harlan D.M. (2016). The MAFB transcription factor impacts islet alpha-cell function in rodents and represents a unique signature of primate islet beta-cells. Am. J. Physiol. Endocrinol. Metab..

[68] Matsuoka T.A., Zhao L., Artner I., Jarrett H.W., Friedman D., Means A., Stein R. (2003). Members of the large Maf transcription family regulate insulin gene transcription in islet beta cells. Mol. Cell. Biol..

