# Supplementary material for: MafA Expression Preserves Immune Homeostasis in Human and Mouse Islets
Source: Genes (Basel). 2018 Dec 18;9(12):644. doi: 10.3390/genes9120644 (PMC6315686; doi:10.3390/genes9120644)
Supplement: Supplementary file 1 [file genes-09-00644-s001.pdf]

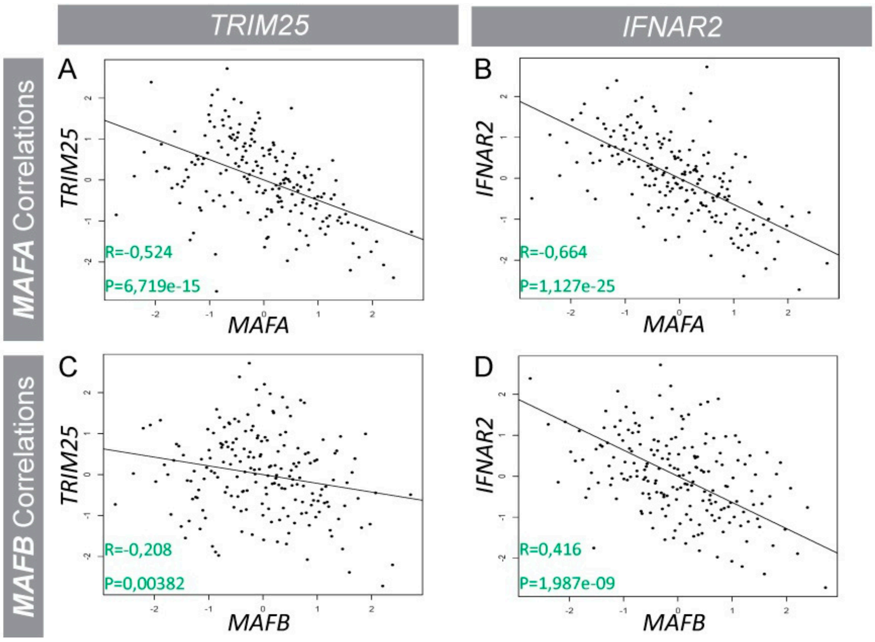

Figure S1: *MAFA* and *MAFB* are negatively correlated with IFI genes. (A–D) Individual correlations of (A,B) *MAFA* and (C,D) *MAFB* with TRIM25 and IFNAR2. (A–D) Spearman correlations (R) and *p*-values are indicated in the respective plots.

Table S1: Quantitative PCR primer sequences. Primer sequences of all genes used in the quantitative PCR experiments.

Gene primer sequences used in quantitative PCR reactions

**Gene**	**Forward primer**	**Reverse primer**
	Housekeeping genes	
HPRT	5’-AGCCCCAAAATGGTTAAGGT-3’	5’-CAAGGGCATATCCAACAACA-3’
Actin-β	5’-GCTTCTTTGCAGCTCCTTCGTTG-3’	5’-TTTGCACATGCCGGAGCCGTTGT-3’
TBP	5’-CTACCGTGAATCTTGGCTGTAA-3’	5’-GTTGTCCGTGGCTCTCTTATT-3’
PPIA	5’-GTGGTCTTTGGGAAGGTGAA-3’	5’-CAGTCGGAAATGGTGATCTTCT-3’
	Cytokine genes	
Ifnβ1	5’-CACAGCCCTCTCCATCAACT-3’	5’-TCCCACGTCAATCTTTCCTC-3’
Ifnα	5’-AGTGAGGTGACCCAGCAGAT-3’	5’-CAGGGGCTGTGTTTCTTCTC-3’
Tnf	5’-CAGGCGGTGCCTATGTCTC-3’	5’-CGATCACCCCGAAGTTCAGTA-3’
IL6	5’-CTGCAAGAGACTTCCATCCAG-3’	5’-AGTGGTATAGACAGGTCTGTT-3’
Cxcl10	5’-GAGCAGAGATGTCTGAATCCG-3’	5’-TGTGCGTGGCTTCACTC-3’
	Anti-viral genes	
Mda5	5’-GTGCCAATCTTGATGCCTTTAC-3’	5’-CTCTGGTGTCATCAGCAATCA-3’
Rig1	5’-CTGACATTCGAGTGGTTGAGA-3’	5’-TGGTTTAGGGTGTGGCTTAC-3’
	Coxsackie and adenoviral receptor gene	
CAR	5’-TGATAACCAGATAGTGGATCAAGTG-3’	5’-TTTATAGATGCGTCGCCAGAC-3’

Tables S2 and S3: Genes list from PathCards pathway unification database. Tables present gene lists from IFN and anti-viral signaling (Table S2A), cytokine and SOCS signaling (Table S2B), T1D susceptibility genes (Table S3A), and T2D susceptibility genes (Table S3B) with their respective *MAFA* and *MAFB* gene correlation (GC) and *p*-values.

Table S2A

MAFA and _MAFB_ co-expression correlations with genes involved in IFN response and anti-viral signaling

Gene name	GeneID	MAFA_GC	*P*-value	MAFB_GC	*P*-value
ADAR	ENSG00000160710	**-0,48**	2.5866477004417e-12	**0,27**	0.000128472739365877
AFAP1	ENSG00000196526	**-0,22**	0.00254180989078645	**-0,27**	0.000119528225769204
AGER	ENSG00000204305	**0,39**	3.59588223542614e-08	**-0,17**	0.0174285717422343
AKT1	ENSG00000142208	**0,49**	4.07315761278472e-13	**-0,12**	0.0868735039250571
AKT2	ENSG00000105221	**0,49**	3.72634275580924e-13	**-0,19**	0.00818748202932539
AKT3	ENSG00000117020	**0,15**	0.0403729970246283	**0,58**	1.18585366248312e-18
APP	ENSG00000142192	**-0,47**	3.89752983276076e-12	**0,16**	0.0240831635670573
ATF2	ENSG00000115966	**-0,30**	3.2891616577512e-05	**0,32**	8.62644875169852e-06
ATG12	ENSG00000145782	**-0,64**	1.69389230749401e-23	**-0,20**	0.00675881239009479
ATG5	ENSG00000057663	**-0,47**	4.90127566011895e-12	**-0,31**	1.11914538411058e-05
AZI2	ENSG00000163512	**-0,24**	0.000980419055712147	**0,09**	0.200308193683534
BECN1	ENSG00000126581	**-0,60**	1.06372871425917e-19	**-0,18**	0.0139385659320826
BRCA1	ENSG00000012048	**-0,32**	8.04821881966992e-06	**0,17**	0.0166236166214121
BST2	ENSG00000130303	**0,20**	0.00654340416103118	**-0,18**	0.0140068959553135
CALM1	ENSG00000198668	**-0,23**	0.00171352867689847	**0,47**	4.1178448492596e-12
CALM2	ENSG00000143933	**-0,68**	3.47715337791891e-27	**-0,21**	0.00303439391972829
CALM3	ENSG00000160014	**-0,14**	0.0504772536289049	**-0,04**	0.59804089178043
CAMK2A	ENSG00000070808	**0,04**	0.559925961380528	**0,03**	0.690815171680589
CAMK2B	ENSG00000058404	**0,74**	1.32581980065863e-34	**0,48**	2.01245174844293e-12
CAMK2D	ENSG00000145349	**-0,07**	0.308804696441233	**0,44**	1.26025680881935e-10
CAMK2G	ENSG00000148660	**0,09**	0.229968443341259	**0,56**	4.74561885427332e-17
CASP1	ENSG00000137752	**-0,37**	1.27731924197375e-07	**-0,28**	9.59804207795709e-05
CASP10	ENSG00000003400	**-0,50**	1.70790007118818e-13	**-0,34**	1.45270888085695e-06
CASP8	ENSG00000064012	**-0,50**	2.00944932332427e-13	**-0,40**	8.97088158582183e-09
CBL	ENSG00000110395	**-0,55**	3.46855474788441e-16	**0,05**	0.504839833517787
CD14	ENSG00000170458	**0,63**	1.80968303395632e-22	**0,10**	0.15297374967771
CDKN1A	ENSG00000124762	**0,21**	0.00313834927732949	**-0,13**	0.0807422336973029
CEBPB	ENSG00000172216	**0,60**	2.9899164695653e-20	**-0,17**	0.0189782157002947
CHUK	ENSG00000213341	**-0,34**	1.1178268897534e-06	**0,20**	0.00625863331816267
CIITA	ENSG00000179583	**0,16**	0.0251925400006313	**0,05**	0.454992654836226
CREB1	ENSG00000118260	**-0,50**	2.03336285650518e-13	**0,13**	0.0669786486565456
CREBBP	ENSG00000005339	**0,21**	0.00401466401809987	**-0,14**	0.0520105793553384
CRK	ENSG00000167193	**-0,75**	8.05055390381393e-36	**-0,30**	2.82393030151967e-05
CRKL	ENSG00000099942	**-0,32**	4.76681209703592e-06	**0,30**	3.16490373461747e-05
CXCL10	ENSG00000169245	**-0,20**	0.00635049523593809	**-0,30**	1.90252887758536e-05
CXCL12	ENSG00000107562	**-0,25**	0.000383990851726459	**-0,39**	2.10479387532965e-08
CXCL8	ENSG00000169429	**-0,42**	1.12097774486885e-09	**-0,54**	1.55144253234339e-15
CXCL9	ENSG00000138755	**-0,21**	0.00288462294587905	**-0,21**	0.00355173584180992
CYBB	ENSG00000165168	**-0,44**	2.64750126097317e-10	**-0,10**	0.162358053660834
CYLD	ENSG00000083799	**-0,24**	0.000867446080372755	**0,22**	0.00257779403575003
DAPK1	ENSG00000196730	**-0,19**	0.0102461393968577	**0,01**	0.885551992977293
DDX17	ENSG00000100201	**0,02**	0.834030117120401	**0,00**	0.96295952316099
DDX3X	ENSG00000215301	**-0,45**	4.97326746490228e-11	**-0,09**	0.194963106377314
DDX3Y	ENSG00000067048	**-0,21**	0.00310636883519242	**-0,05**	0.487427870641955
DDX58	ENSG00000107201	**-0,42**	9.47228472906936e-10	**-0,23**	0.00110291790660329
DHX36	ENSG00000174953	**-0,45**	1.01826907853331e-10	**0,20**	0.00500524683407353
DHX58	ENSG00000108771	**0,47**	4.42466552537902e-12	**-0,22**	0.00206682956848339

DHX9	ENSG00000135829	**-0,51**	4.06940241394339e-14	**-0,12**	0.11122258089935
EGR1	ENSG00000120738	**-0,14**	0.0623343681152722	**-0,22**	0.00280085718660578
EIF2AK2	ENSG00000055332	**-0,46**	3.30817892243563e-11	**0,15**	0.0406389192141145
EIF4A1	ENSG00000161960	**-0,74**	2.31419729734339e-34	**-0,31**	1.32143630545985e-05
EIF4B	ENSG00000063046	**-0,59**	1.13502892661456e-19	**0,00**	0.960161084589656
EIF4E	ENSG00000151247	**-0,59**	2.91968333681233e-19	**-0,01**	0.913889836816078
EIF4EBP1	ENSG00000187840	**0,40**	7.69254668744038e-09	**-0,29**	5.82275126060127e-05
EP300	ENSG00000100393	**-0,21**	0.00340861626434952	**0,00**	0.990585855749299
FADD	ENSG00000168040	**-0,15**	0.0356583211386548	**-0,22**	0.00279319763901373
FANCC	ENSG00000158169	**-0,15**	0.0415773279297585	**0,14**	0.0502005998938999
FYN	ENSG00000010810	**-0,11**	0.115624618811205	**0,36**	4.65577043086155e-07
GAB2	ENSG00000033327	**-0,34**	1.31467093679976e-06	**-0,07**	0.367957087429214
GBP1	ENSG00000117228	**-0,54**	3.72749092694847e-16	**-0,39**	1.62642204239511e-08
GBP2	ENSG00000162645	**-0,48**	2.60404210434064e-12	**-0,48**	2.35061797063963e-12
HERC5	ENSG00000138646	**0,05**	0.506459355392549	**0,50**	2.87793406986351e-13
HLA-A	ENSG00000206503	**0,41**	3.17676782898581e-09	**0,05**	0.501993587554691
HLA-B	ENSG00000234745	**-0,07**	0.321245752822301	**-0,37**	1.35154815601213e-07
HLA-C	ENSG00000204525	**0,22**	0.00177092256548731	**-0,05**	0.493859111819826
HLA-E	ENSG00000204592	**0,16**	0.0291932833068203	**-0,28**	9.86111154029346e-05
HLA-F	ENSG00000204642	**0,48**	3.0844601875437e-12	**-0,25**	0.000591139503654884
HLA-G	ENSG00000204632	**0,05**	0.482261735708689	**-0,31**	9.9969053922563e-06
HLA-H	ENSG00000206341	**0,18**	0.0140794221492256	**0,09**	0.239411023029804
HMGB1	ENSG00000189403	**-0,47**	1.09830958412856e-11	**-0,19**	0.00703428082896763
ICAM1	ENSG00000090339	**-0,26**	0.000310326558739677	**-0,38**	7.74971946141322e-08
IFI16	ENSG00000163565	**-0,34**	1.67670760690685e-06	**-0,27**	0.000192580545122838
IFI27	ENSG00000165949	**0,06**	0.417463327642741	**-0,18**	0.012460364710499
IFI27L1	ENSG00000165948	**0,05**	0.510543782695452	**0,25**	0.000530577354782791
IFI27L2	ENSG00000119632	**0,69**	9.76574874205656e-29	**0,18**	0.0117519093294205
IFI30	ENSG00000216490	**0,08**	0.295313404702536	**-0,12**	0.110463236495231
IFI35	ENSG00000068079	**0,08**	0.291372865987342	**0,02**	0.766252150166224
IFI44	ENSG00000137965	**-0,34**	1.16965246796782e-06	**-0,42**	1.28196123577402e-09
IFI44L	ENSG00000137959	**-0,24**	0.000934117970662363	**-0,27**	0.000119049051259401
IFI6	ENSG00000126709	**0,60**	2.28537513168798e-20	**0,14**	0.0534002297273771
IFIH1	ENSG00000115267	**-0,35**	4.95295621421065e-07	**0,19**	0.0080246166595001
IFIT1	ENSG00000185745	**-0,10**	0.168179329055682	**0,13**	0.0790116548077697
IFIT2	ENSG00000119922	**-0,39**	1.81926495916762e-08	**-0,37**	1.77032737388667e-07
IFIT3	ENSG00000119917	**-0,46**	1.83893380809121e-11	**-0,36**	2.66527425198204e-07
IFIT5	ENSG00000152778	**-0,40**	1.38063670814945e-08	**0,22**	0.00235545560046322
IFITM1	ENSG00000185885	**-0,12**	0.0868732507529924	**-0,10**	0.182545759924403
IFITM10	ENSG00000244242	**0,29**	5.56725742440848e-05	**-0,16**	0.0243587877409559
IFITM2	ENSG00000185201	**-0,08**	0.259989669232281	**-0,40**	6.3595868417778e-09
IFITM3	ENSG00000142089	**0,18**	0.0128806254229862	**-0,45**	4.72199476985729e-11
IFNA20P	ENSG00000226393	**0,21**	0.00416376696711289	**-0,04**	0.538358637859135
IFNAR1	ENSG00000142166	**-0,63**	1.93927974544317e-22	**-0,26**	0.000304375990188567
IFNAR2	ENSG00000159110	**-0,66**	1.12799580148387e-25	**-0,42**	1.9875301607214e-09
IFNGR1	ENSG00000027697	**-0,61**	9.20222557434055e-21	**-0,47**	6.89646309761597e-12
IFNGR2	ENSG00000159128	**-0,51**	2.75065468913454e-14	**-0,54**	4.3989143154181e-16
IFNLR1	ENSG00000185436	**-0,31**	1.556330238438e-05	**-0,13**	0.0754043913196151
IKBKB	ENSG00000104365	**0,00**	0.955333101431861	**-0,29**	6.32388919004456e-05
IL10	ENSG00000136634	**-0,35**	6.62961598191475e-07	**-0,14**	0.0481751827565998
IL12A	ENSG00000168811	**0,00**	0.991110726408829	**-0,38**	7.9570963243035e-08
IL15	ENSG00000164136	**-0,22**	0.00267988795387774	**-0,70**	6.9321397775139e-29
IL16	ENSG00000172349	**-0,06**	0.424249139118085	**-0,01**	0.857373004345726
IL18	ENSG00000150782	**-0,27**	0.000193778534666499	**-0,32**	4.62993766015631e-06
IL1A	ENSG00000115008	**-0,32**	8.45224131569533e-06	**-0,30**	2.66784939150809e-05
IL1B	ENSG00000125538	**-0,35**	7.39860946753494e-07	**-0,35**	8.80488246544108e-07
IL33	ENSG00000137033	**-0,39**	3.03371256792028e-08	**-0,42**	9.27237350542814e-10
IL6	ENSG00000136244	**-0,38**	6.23851661010328e-08	**-0,54**	1.04547716421021e-15

IP6K2	ENSG00000068745	**0,30**	2.25135147047044e-05	**0,07**	0.324641430677502
IRF1	ENSG00000125347	**-0,22**	0.00218498430516907	**-0,45**	6.8595246836266e-11
IRF2	ENSG00000168310	**-0,53**	2.64551578741172e-15	**-0,12**	0.0860556588295567
IRF3	ENSG00000126456	**0,62**	1.55820327947092e-21	**-0,13**	0.0674161842953076
IRF4	ENSG00000137265	**-0,37**	1.1784521145191e-07	**-0,50**	3.2774625407313e-13
IRF5	ENSG00000128604	**0,52**	6.57680600283766e-15	**-0,13**	0.0848245154461347
IRF6	ENSG00000117595	**-0,56**	4.31345058997722e-17	**0,07**	0.317195808715754
IRF7	ENSG00000185507	**0,66**	2.2146313704963e-25	**-0,12**	0.108319133878486
IRF8	ENSG00000140968	**-0,17**	0.0189482704504927	**-0,20**	0.00606151359148182
IRF9	ENSG00000213928	**-0,01**	0.927355270888246	**-0,11**	0.13027341850353
IRS1	ENSG00000169047	**0,19**	0.00779396677946712	**-0,36**	2.89957043440865e-07
IRS2	ENSG00000185950	**0,26**	0.000226329855985828	**0,23**	0.00164737210953704
ISG15	ENSG00000187608	**0,61**	3.31669997958626e-21	**-0,13**	0.0836352099427672
ISG20	ENSG00000172183	**0,40**	1.36808822637563e-08	**-0,27**	0.000146216702121402
ITCH	ENSG00000078747	**-0,60**	4.22823020929935e-20	**0,00**	0.995443777689389
ITPR1	ENSG00000150995	**-0,19**	0.00737111439320625	**0,36**	2.55509115938012e-07
ITPR2	ENSG00000123104	**-0,43**	3.87773800527843e-10	**-0,02**	0.777396709371253
ITPR3	ENSG00000096433	**0,52**	1.4137230967383e-14	**0,03**	0.644539315465679
JAK1	ENSG00000162434	**-0,52**	1.21965899014069e-14	**-0,17**	0.0181285138677532
JAK2	ENSG00000096968	**-0,21**	0.00293620050676593	**0,31**	1.15950395778076e-05
LCK	ENSG00000182866	**-0,15**	0.0353462625797426	**-0,23**	0.00136232130345569
LY96	ENSG00000154589	**-0,22**	0.00195846263185404	**-0,13**	0.0753037824128098
MAP2K1	ENSG00000169032	**-0,42**	1.0089549299884e-09	**-0,13**	0.0641132819324937
MAP2K3	ENSG00000034152	**0,31**	1.74885548526188e-05	**-0,30**	2.24391426857431e-05
MAP2K6	ENSG00000108984	**-0,40**	1.53752344947262e-08	**0,19**	0.00843758230584174
MAP3K1	ENSG00000095015	**-0,59**	2.14954752454161e-19	**-0,38**	5.75968519951562e-08
MAP3K11	ENSG00000173327	**0,64**	3.91960507895763e-23	**-0,08**	0.276514101965175
MAP3K4	ENSG00000085511	**-0,31**	1.25307634064807e-05	**0,26**	0.000275213929728696
MAP3K7	ENSG00000135341	**-0,35**	6.39003437413233e-07	**-0,01**	0.867092351786451
MAPK1	ENSG00000100030	**-0,60**	2.99200936810869e-20	**0,08**	0.251282465884628
MAPK10	ENSG00000109339	**0,00**	0.962978066789964	**0,63**	1.24976684735071e-22
MAPK11	ENSG00000185386	**0,64**	1.33530520718186e-23	**0,08**	0.268922126268205
MAPK12	ENSG00000188130	**0,70**	3.25251537512553e-29	**0,02**	0.772127715624114
MAPK13	ENSG00000156711	**0,42**	2.1806270771068e-09	**0,09**	0.206238498899922
MAPK14	ENSG00000112062	**-0,44**	1.17566206635571e-10	**0,05**	0.507090038075621
MAPK3	ENSG00000102882	**0,36**	2.87917061872028e-07	**0,30**	2.0009180219269e-05
MAPK8	ENSG00000107643	**-0,56**	2.23804290203971e-17	**-0,30**	1.96903338705634e-05
MAPK9	ENSG00000050748	**-0,46**	1.64214884535091e-11	**0,20**	0.00516805948761377
MAPKAP1	ENSG00000119487	**-0,41**	2.42341639458969e-09	**-0,41**	5.9112783825463e-09
MAVS	ENSG00000088888	**0,04**	0.579983207901823	**0,02**	0.83104014829524
MCM5	ENSG00000100297	**0,25**	0.000389994148458065	**-0,21**	0.00307861805674677
MLST8	ENSG00000167965	**0,70**	6.58810809014195e-30	**0,05**	0.49115449048096
MTOR	ENSG00000198793	**-0,46**	1.39801399115379e-11	**0,03**	0.700528947537056
MX1	ENSG00000157601	**-0,18**	0.015182873912748	**-0,26**	0.000298574134706061
MX2	ENSG00000183486	**-0,20**	0.00461039956638195	**-0,20**	0.0065061668848554
MYC	ENSG00000136997	**-0,17**	0.0168645538571789	**-0,49**	3.93334794486251e-13
MYD88	ENSG00000172936	**-0,63**	1.30792485750974e-22	**-0,47**	7.90972736361841e-12
NFKB1	ENSG00000109320	**-0,59**	1.41337345847154e-19	**-0,40**	1.27178951836757e-08
NFKB2	ENSG00000077150	**0,22**	0.00270211457539767	**-0,42**	1.95754158193685e-09
NFKBIA	ENSG00000100906	**-0,22**	0.00228519241789342	**-0,59**	5.63470401469128e-19
NFKBIB	ENSG00000104825	**0,39**	3.51981854643213e-08	**-0,19**	0.00737618848454116
NFKBIE	ENSG00000146232	**0,27**	0.00017205593019912	**-0,46**	3.34329888230106e-11
NLRC5	ENSG00000140853	**0,25**	0.000597976325727681	**-0,31**	1.17107390335828e-05
NLRX1	ENSG00000160703	**0,62**	2.53561542128123e-21	**0,06**	0.393120541657589
NOS2	ENSG00000007171	**0,04**	0.553775969547528	**-0,01**	0.849052646270222
OAS1	ENSG00000089127	**-0,15**	0.0328201484234877	**0,05**	0.480262960349265
OAS2	ENSG00000111335	**-0,24**	0.000864956449482526	**-0,08**	0.252654097541656
OAS3	ENSG00000111331	**-0,26**	0.000330722148169071	**-0,14**	0.0474655004707886

OASL	ENSG00000135114		**-0,28**	0.000104403949329355		**-0,29**	3.71092703163127e-05
OTUD5	ENSG00000068308		**0,29**	6.14263040136555e-05		**0,01**	0.87548682456972
PCBP2	ENSG00000197111		**-0,59**	5.60588267442549e-19		**-0,22**	0.00202972390267889
PDCD4	ENSG00000150593		**-0,42**	2.1729409110657e-09		**0,06**	0.442384894582261
PDPK1	ENSG00000140992		**-0,03**	0.729074006098694		**0,32**	8.23945206464345e-06
PIAS1	ENSG00000033800		**-0,28**	9.55460502211832e-05		**0,38**	4.92018850220099e-08
PIAS3	ENSG00000131788		**0,19**	0.00845215288768675		**0,13**	0.0730097499669709
PIAS4	ENSG00000105229		**0,79**	2.5433816202907e-41		**0,16**	0.0283593171943679
PIK3CA	ENSG00000121879		**-0,67**	2.39529234700326e-26		**-0,17**	0.0210010819676031
PIK3CB	ENSG00000051382		**-0,63**	4.05405996223875e-22		**-0,20**	0.00456660588519594
PIK3CD	ENSG00000171608		**0,29**	5.85616427793295e-05		**-0,34**	1.86148637990212e-06
PIK3R1	ENSG00000145675		**-0,70**	4.94075866857068e-29		**-0,09**	0.19404408185396
PIK3R2	ENSG00000105647		**0,77**	6.52792398222713e-39		**0,42**	2.10515977066689e-09
PIK3R3	ENSG00000117461		**-0,05**	0.526593151012579		**0,55**	1.10202969066579e-16
PIN1	ENSG00000127445		**0,73**	1.91107049191611e-33		**0,10**	0.154176054860317
PLCG2	ENSG00000197943		**-0,16**	0.0230647123569113		**0,20**	0.00670425002113378
PML	ENSG00000140464		**0,61**	1.82685940214907e-20		**-0,07**	0.305318714987008
POLR1C	ENSG00000171453		**-0,65**	6.72601292243326e-24		**-0,13**	0.0787710947675291
POLR1D	ENSG00000186184		**-0,06**	0.420677839503687		**-0,47**	4.01528347944659e-12
POLR2E	ENSG00000099817		**0,69**	2.2828896680807e-28		**0,00**	0.951618086529431
POLR2F	ENSG00000100142		**0,60**	7.13476459649141e-20		**-0,08**	0.271306099048625
POLR2H	ENSG00000163882		**-0,16**	0.0269426954024577		**-0,41**	5.04454933891137e-09
POLR2K	ENSG00000147669		**-0,46**	2.07301605992995e-11		**0,04**	0.626605996919733
POLR2L	ENSG00000177700		**0,62**	5.2426411038459e-22		**-0,11**	0.122441216082193
POLR3A	ENSG00000148606		**-0,39**	2.44187865131178e-08		**0,26**	0.000256085395636696
POLR3B	ENSG00000013503		**-0,43**	4.75155332246433e-10		**0,13**	0.0674830671013431
POLR3C	ENSG00000186141		**-0,73**	1.07674710882475e-33		**-0,34**	1.8282085847092e-06
POLR3D	ENSG00000168495		**-0,38**	4.00090315212754e-08		**0,03**	0.674412646468754
POLR3E	ENSG00000058600		**0,49**	4.8213937289235e-13		**-0,13**	0.0816767426354021
POLR3F	ENSG00000132664		**-0,26**	0.000337003956817899		**-0,09**	0.194474995439798
POLR3G	ENSG00000113356		**0,12**	0.0951089131873933		**0,01**	0.94427891029144
POLR3GL	ENSG00000121851		**0,09**	0.207137666035868		**0,53**	5.7375730845458e-15
POLR3H	ENSG00000100413		**0,50**	1.13306605884702e-13		**0,00**	0.954956478957725
POLR3K	ENSG00000161980		**-0,14**	0.0510724430660249		**0,17**	0.020543450809824
PRKCA	ENSG00000154229		**-0,38**	6.21125575143855e-08		**0,14**	0.0617953671253469
PRKCD	ENSG00000163932		**-0,35**	8.07477316843992e-07		**-0,18**	0.0106597716713838
PRMT1	ENSG00000126457		**0,19**	0.00840133714714741		**-0,12**	0.0966882497966188
PSMB8	ENSG00000204264		**-0,61**	3.10896602614374e-21		**-0,37**	1.56551206915383e-07
PSMB9	ENSG00000240065		**-0,07**	0.31394140105893		**-0,33**	2.37041173053431e-06
PTGES2	ENSG00000148334		**0,74**	2.04536819350297e-34		**0,01**	0.879309167688131
PTK2B	ENSG00000120899		**0,18**	0.0104280244768455		**-0,09**	0.227088637359813
PTPN1	ENSG00000196396		**0,13**	0.0757562099293001		**0,19**	0.00894536646261136
PTPN11	ENSG00000179295		**-0,44**	2.83742915818007e-10		**0,19**	0.00849151413351793
PTPN6	ENSG00000111679		**0,28**	9.07159554868796e-05		**-0,09**	0.193182499885867
PTPRC	ENSG00000081237		**-0,40**	1.48074898772267e-08		**-0,12**	0.0917501247852444
PYCARD	ENSG00000103490		**0,50**	9.96104671154407e-14		**-0,16**	0.0309530348574785
RAC1	ENSG00000136238		**-0,57**	4.08102412241896e-18		**-0,25**	0.000530049895111784
RACK1	ENSG00000204628		**-0,51**	4.53203202169132e-14		**-0,42**	1.06766533445868e-09
RAP1A	ENSG00000116473		**-0,62**	2.68365683637027e-21		**-0,06**	0.375865435998495
RAPGEF1	ENSG00000107263		**0,47**	7.21420063705013e-12		**-0,15**	0.0328580551711442
REG1A	ENSG00000115386		**-0,50**	1.35020805961934e-13		**-0,50**	1.51155536234944e-13
REL	ENSG00000162924		**-0,52**	7.85048830110015e-15		**-0,14**	0.0478115197701923
RELA	ENSG00000173039		**0,33**	2.60509100663762e-06		**-0,32**	4.81257582740091e-06
RELB	ENSG00000104856		**0,34**	1.49315930795871e-06		**-0,40**	1.15561076208071e-08
RIPK1	ENSG00000137275		**-0,55**	8.4800183856022e-17		**-0,05**	0.461793480056313
RIPK3	ENSG00000129465		**-0,20**	0.00530966421272976		**-0,42**	1.15219531942584e-09
RNASEL	ENSG00000135828		**-0,23**	0.00109628575311977		**0,14**	0.060581002434791
RNF125	ENSG00000101695		**-0,51**	5.82151579330119e-14		**-0,29**	5.12187430003616e-05

RNF135	ENSG00000181481	**-0,22**	0.00260534700927174	**0,18**	0.0150170743464126
RNF216	ENSG00000011275	**0,32**	6.44379989509249e-06	**0,33**	2.97326583175837e-06
RPS27A	ENSG00000143947	**-0,52**	1.29280938389443e-14	**-0,34**	1.10727580600848e-06
RPS6	ENSG00000137154	**-0,68**	3.50912401250252e-27	**-0,36**	2.15580365028761e-07
RPS6KA4	ENSG00000162302	**0,60**	6.93114278622626e-20	**-0,13**	0.0645481035396358
RPS6KA5	ENSG00000100784	**-0,44**	2.27624067378863e-10	**-0,45**	9.60597349232598e-11
RPS6KB1	ENSG00000108443	**-0,56**	2.24965118646076e-17	**-0,32**	4.80619939811087e-06
RPTOR	ENSG00000141564	**0,59**	1.3986796784502e-19	**0,04**	0.615954005030012
RSAD2	ENSG00000134321	**-0,22**	0.00234969317224505	**-0,19**	0.00789868450016011
S100B	ENSG00000160307	**-0,12**	0.104800630357125	**0,24**	0.000667004503289965
SAA1	ENSG00000173432	**-0,20**	0.00533019861105571	**-0,17**	0.0223168546849501
SAMHD1	ENSG00000101347	**-0,43**	4.29490781061603e-10	**-0,09**	0.239278167595729
SIKE1	ENSG00000052723	**-0,49**	4.9648970395018e-13	**-0,01**	0.856104626085877
SMAD7	ENSG00000101665	**0,44**	2.95199093677303e-10	**0,05**	0.504104942979813
SNW1	ENSG00000100603	**-0,48**	2.9315833496172e-12	**-0,06**	0.438683105845325
SOCS1	ENSG00000185338	**0,62**	1.13362876375065e-21	**-0,11**	0.135148843402378
SOCS3	ENSG00000184557	**0,24**	0.000917143429051332	**-0,39**	1.89577019821364e-08
SPI1	ENSG00000066336	**0,49**	9.08606474832705e-13	**-0,10**	0.173284886774545
SRC	ENSG00000197122	**0,08**	0.262402472097997	**-0,39**	2.39005445263932e-08
STAT1	ENSG00000115415	**-0,46**	1.4084182737644e-11	**0,08**	0.270253906811317
STAT2	ENSG00000170581	**-0,39**	1.74298679914003e-08	**0,06**	0.42665854407596
STAT3	ENSG00000168610	**-0,46**	1.24763123118793e-11	**0,13**	0.0793916043044484
STAT4	ENSG00000138378	**-0,02**	0.759118455330614	**0,47**	5.63943268292532e-12
STAT5A	ENSG00000126561	**-0,17**	0.0193706168094797	**-0,35**	8.63634668143367e-07
TANK	ENSG00000136560	**-0,76**	7.68308912814543e-38	**-0,31**	1.21568714013484e-05
TAP1	ENSG00000168394	**-0,42**	1.73146238727583e-09	**-0,44**	2.13727148818295e-10
TAX1BP1	ENSG00000106052	**-0,55**	1.28351384299721e-16	**-0,07**	0.328172593866926
TBK1	ENSG00000183735	**-0,29**	3.91972671249438e-05	**-0,17**	0.0192847214049868
TBKBP1	ENSG00000198933	**0,64**	5.27569005744411e-23	**0,00**	0.946312658712726
TICAM1	ENSG00000127666	**0,63**	1.40496099616993e-22	**-0,10**	0.149260318141619
TICAM2	ENSG00000243414	**0,06**	0.413887218692825	**0,09**	0.22107127916853
TIRAP	ENSG00000150455	**-0,24**	0.00087036894631872	**0,11**	0.12756277882149
TKFC	ENSG00000149476	**0,57**	5.43588946004606e-18	**0,02**	0.819657856220774
TLR3	ENSG00000164342	**-0,47**	4.00939827290733e-12	**-0,39**	2.31978643767263e-08
TLR4	ENSG00000136869	**-0,43**	7.82742680718726e-10	**-0,07**	0.357624727027409
TMEM173	ENSG00000184584	**-0,11**	0.11548464364466	**-0,11**	0.123682115112946
TMEM256-PLSCR3	ENSG00000187838	**0,57**	4.94436605652343e-18	**-0,08**	0.270944163989383
TNF	ENSG00000232810	**-0,17**	0.0219343961824148	**-0,33**	2.27682620675884e-06
TNFAIP3	ENSG00000118503	**-0,35**	5.59862265015102e-07	**-0,54**	1.00186587810008e-15
TOMM70	ENSG00000154174	**-0,45**	4.46333941640318e-11	**0,06**	0.398543697012559
TRADD	ENSG00000102871	**0,64**	1.44562537015072e-23	**-0,11**	0.126888734861629
TRAF2	ENSG00000127191	**0,62**	1.04382589432806e-21	**-0,03**	0.656224728116167
TRAF3	ENSG00000131323	**0,56**	1.74566636174895e-17	**0,09**	0.213743615770432
TRAF6	ENSG00000175104	**-0,55**	8.75714446453464e-17	**-0,03**	0.708375135881821
TREX1	ENSG00000213689	**0,56**	3.19332791810519e-17	**-0,11**	0.136949702291795
TRIM10	ENSG00000204613	**-0,23**	0.00156289312372338	**-0,24**	0.000974832087318299
TRIM11	ENSG00000154370	**0,56**	2.18117330494137e-17	**-0,12**	0.101633916584728
TRIM13	ENSG00000204977	**-0,21**	0.00329184242278166	**0,03**	0.70871019540628
TRIM14	ENSG00000106785	**0,20**	0.00517951985555743	**-0,03**	0.700124652637551
TRIM15	ENSG00000204610	**0,06**	0.407606701103027	**-0,14**	0.0548654665661716
TRIM16	ENSG00000221926	**-0,41**	3.74669092039446e-09	**-0,22**	0.00253787350219161
TRIM16L	ENSG00000108448	**-0,41**	5.98827791901165e-09	**-0,13**	0.0832113088151327
TRIM17	ENSG00000162931	**0,28**	7.0171863253822e-05	**-0,15**	0.0400242635409909
TRIM2	ENSG00000109654	**-0,06**	0.404391480392056	**0,53**	3.48048683374772e-15
TRIM21	ENSG00000132109	**-0,52**	1.69581064085608e-14	**-0,23**	0.00114695604613319
TRIM22	ENSG00000132274	**-0,29**	5.60814749934302e-05	**-0,20**	0.00657301357749269
TRIM23	ENSG00000113595	**-0,24**	0.00106071157031425	**0,30**	2.66713333087657e-05
TRIM24	ENSG00000122779	**-0,56**	2.05264525241692e-17	**-0,08**	0.296038338205718

TRIM25	ENSG00000121060	**-0,52**	6.71996864034073e-15	**-0,21**	0.00382735405395848
TRIM26	ENSG00000234127	**-0,17**	0.0188485001001899	**-0,29**	4.20187009111721e-05
TRIM27	ENSG00000204713	**-0,26**	0.000272990296265981	**-0,08**	0.245735717831493
TRIM28	ENSG00000130726	**0,45**	7.02028911433587e-11	**-0,12**	0.104425585181782
TRIM29	ENSG00000137699	**-0,11**	0.136420081544235	**-0,19**	0.00917730597847544
TRIM3	ENSG00000110171	**0,65**	2.28360443101778e-24	**0,18**	0.0153836516626748
TRIM31	ENSG00000204616	**-0,15**	0.0351680587470194	**-0,25**	0.000410941117778941
TRIM32	ENSG00000119401	**-0,50**	3.06309645124751e-13	**0,06**	0.407611804186044
TRIM33	ENSG00000197323	**-0,41**	4.11854950265433e-09	**0,10**	0.160088315725925
TRIM34	ENSG00000258659	**-0,47**	4.18960538952854e-12	**-0,21**	0.00307383823744581
TRIM35	ENSG00000104228	**-0,01**	0.934064622295998	**0,12**	0.101272344512958
TRIM36	ENSG00000152503	**-0,43**	5.82980472562968e-10	**-0,25**	0.000604174657109914
TRIM37	ENSG00000108395	**-0,15**	0.0413269256599019	**0,48**	1.79861350871942e-12
TRIM38	ENSG00000112343	**-0,51**	3.94248494347381e-14	**-0,31**	1.5450032546911e-05
TRIM39	ENSG00000204599	**-0,14**	0.0474505278124327	**0,11**	0.132556193261022
TRIM39-RPP21	ENSG00000248167	**0,60**	3.6248595677376e-20	**-0,07**	0.317128305900084
TRIM4	ENSG00000146833	**-0,13**	0.0631532512088453	**0,40**	6.41711007654757e-09
TRIM41	ENSG00000146063	**0,53**	3.21216373174691e-15	**-0,03**	0.716586627958111
TRIM44	ENSG00000166326	**-0,57**	8.62323511412767e-18	**0,05**	0.534847210650807
TRIM45	ENSG00000134253	**-0,28**	7.80981009979289e-05	**-0,36**	3.74850625143589e-07
TRIM46	ENSG00000163462	**0,71**	2.6361543793505e-30	**0,18**	0.010887443150471
TRIM47	ENSG00000132481	**0,42**	1.47643880143584e-09	**-0,34**	1.3199292958065e-06
TRIM5	ENSG00000132256	**-0,49**	3.73190027201171e-13	**-0,27**	0.000168554525425517
TRIM50	ENSG00000146755	**0,23**	0.00134237225802692	**-0,19**	0.0101819990336803
TRIM52	ENSG00000183718	**0,24**	0.000637574722293975	**-0,04**	0.572122128641236
TRIM52-AS1	ENSG00000248275	**0,25**	0.000515736123557216	**0,08**	0.274163183875925
TRIM54	ENSG00000138100	**0,39**	2.44401011971338e-08	**-0,29**	3.92635778553277e-05
TRIM55	ENSG00000147573	**-0,01**	0.885213223865771	**0,39**	2.61027390901611e-08
TRIM56	ENSG00000169871	**0,22**	0.00254659398818353	**0,00**	0.996849285750957
TRIM58	ENSG00000162722	**0,05**	0.45141543797027	**0,09**	0.199965775171284
TRIM59	ENSG00000213186	**-0,23**	0.00131951907721887	**0,22**	0.0027207590845368
TRIM6	ENSG00000121236	**-0,56**	4.72032373754496e-17	**-0,39**	2.67008418478172e-08
TRIM60P18	ENSG00000227986	**0,15**	0.0424259589264074	**0,00**	0.976543048100179
TRIM61	ENSG00000183439	**-0,32**	7.56410831920075e-06	**0,21**	0.00298408112157224
TRIM62	ENSG00000116525	**0,11**	0.139952373286212	**0,02**	0.798763084780243
TRIM63	ENSG00000158022	**-0,10**	0.177816128095305	**-0,14**	0.0618388406339085
TRIM65	ENSG00000141569	**0,43**	8.62955850607143e-10	**0,06**	0.401967125570945
TRIM66	ENSG00000166436	**0,09**	0.230875767021715	**-0,04**	0.545006077859981
TRIM67	ENSG00000119283	**0,08**	0.249584764110123	**-0,06**	0.383448353125519
TRIM68	ENSG00000167333	**-0,50**	2.53573098673162e-13	**0,16**	0.0252675494280328
TRIM69	ENSG00000185880	**-0,43**	6.69769668719785e-10	**-0,27**	0.00016951169846237
TRIM6-TRIM34	ENSG00000258588	**-0,50**	1.40409601550081e-13	**-0,23**	0.00171544328588778
TRIM7	ENSG00000146054	**0,48**	2.51082976714821e-12	**0,08**	0.291074324382573
TRIM71	ENSG00000206557	**0,04**	0.590216084808043	**0,07**	0.309221870940347
TRIM73	ENSG00000178809	**0,44**	1.79002193202822e-10	**-0,11**	0.116525394148827
TRIM74	ENSG00000155428	**0,21**	0.00283220874435234	**-0,18**	0.0154366524699441
TRIM8	ENSG00000171206	**0,54**	3.93309820324546e-16	**-0,15**	0.0422667660760826
TRIM9	ENSG00000100505	**0,11**	0.133473306241226	**0,47**	5.41138252119461e-12
TYK2	ENSG00000105397	**0,65**	1.55184202437123e-24	**-0,02**	0.769230270167196
UBA52	ENSG00000221983	**-0,72**	1.86618598734546e-31	**-0,24**	0.000873939378966847
UBA7	ENSG00000182179	**0,05**	0.480049135193552	**-0,23**	0.00158606487991596
UBB	ENSG00000170315	**-0,59**	2.50119210933739e-19	**0,14**	0.0625638107445379
UBC	ENSG00000150991	**-0,56**	2.78723123981906e-17	**-0,07**	0.364084254050423
UBE2D1	ENSG00000072401	**-0,17**	0.0223304402788047	**-0,37**	1.48904201358508e-07
UBE2D2	ENSG00000131508	**-0,12**	0.0896614230489009	**-0,02**	0.767514104162234
UBE2D3	ENSG00000109332	**-0,45**	7.80311120389263e-11	**-0,11**	0.116257490538917
UBE2K	ENSG00000078140	**-0,56**	4.67131082155242e-17	**0,05**	0.524008506943129
UBE2L6	ENSG00000156587	**-0,40**	1.07744726518065e-08	**-0,01**	0.928487466220116

USP18	ENSG00000184979	**-0,22**	0.00230514533456041	**0,38**	4.49955508301541e-08
VAV1	ENSG00000141968	**-0,22**	0.00188089971434898	**-0,11**	0.137469707678826
XAF1	ENSG00000132530	**-0,12**	0.10030354173073	**-0,04**	0.607941869840259
ZAP70	ENSG00000115085	**0,31**	9.52484863713989e-06	**-0,18**	0.0119483164628887

Table S2B

***MAFA* and *MAFB* co-expression correlations with genes involved in cytokine signaling**

Genename	GeneID	MAFA_GC	*P*-value	MAFB_GC	*P*-value
AAAS	ENSG00000094914	**0,06**	0.418353187153833	**0,02**	0.803032573683404
ACTN2	ENSG00000077522	**-0,16**	0.0247037136171466	**-0,51**	6.89902915031085e-14
ADAM17	ENSG00000151694	**-0,72**	1.267694686376e-31	**-0,42**	1.01165290409279e-09
ADAR	ENSG00000160710	**-0,48**	2.5866477004417e-12	**0,27**	0.000128472739365877
AKAP9	ENSG00000127914	**-0,01**	0.850846400836398	**0,29**	3.79979757256914e-05
AKT1	ENSG00000142208	**0,49**	4.07315761278472e-13	**-0,12**	0.0868735039250571
ALOX5	ENSG00000012779	**0,30**	1.87152802173304e-05	**-0,06**	0.437355233947124
ANGPT1	ENSG00000154188	**-0,24**	0.00074241863314501	**0,09**	0.202952411457814
ANXA1	ENSG00000135046	**-0,50**	2.37579656480532e-13	**-0,30**	1.97301562785172e-05
APBB1IP	ENSG00000077420	**-0,15**	0.0325617445019794	**-0,02**	0.745400340305931
ARAF	ENSG00000078061	**0,39**	1.91839473261534e-08	**-0,19**	0.00903695751973378
ARIH1	ENSG00000166233	**-0,60**	4.52948091855036e-20	**-0,05**	0.500349434672052
ARRB1	ENSG00000137486	**-0,35**	6.15697284981389e-07	**0,09**	0.191838526312754
ARRB2	ENSG00000141480	**0,19**	0.00850345391815282	**0,05**	0.531170073130129
ARTN	ENSG00000117407	**0,59**	4.91100952427545e-19	**0,03**	0.690824983544474
B2M	ENSG00000166710	**-0,63**	1.57472746308634e-22	**-0,26**	0.000301092518759588
BATF	ENSG00000156127	**0,15**	0.040132158107048	**-0,13**	0.0763698625994746
BCL2	ENSG00000171791	**-0,61**	1.57569459075146e-20	**-0,37**	1.25077746579985e-07
BCL2L1	ENSG00000171552	**-0,05**	0.485231362791119	**-0,20**	0.00575913089355628
BCL6	ENSG00000113916	**-0,13**	0.0713773538285527	**-0,51**	6.06666734707207e-14
BIRC2	ENSG00000110330	**-0,70**	1.91247074936007e-29	**-0,49**	3.47471121485483e-13
BIRC3	ENSG00000023445	**-0,53**	5.64894897445921e-15	**-0,52**	1.01889991519376e-14
BIRC5	ENSG00000089685	**0,00**	0.973333701574282	**0,15**	0.0340550428876236
BLNK	ENSG00000095585	**-0,43**	7.83727840585589e-10	**-0,42**	2.33253796601925e-09
BRAF	ENSG00000157764	**-0,20**	0.00537578330723444	**0,27**	0.000171517098880588
BRAP	ENSG00000089234	**-0,23**	0.00133923489047767	**0,27**	0.00012032414880185
BRWD1	ENSG00000185658	**-0,01**	0.907683994264766	**-0,01**	0.883376751895466
BST2	ENSG00000130303	**0,20**	0.00654340416103118	**-0,18**	0.0140068959553135
BTC	ENSG00000174808	**-0,21**	0.00292347572146095	**0,16**	0.0258743696278418
BTRC	ENSG00000166167	**-0,41**	2.45485533120055e-09	**0,32**	6.28551551572272e-06
CALM1	ENSG00000198668	**-0,23**	0.00171352867689847	**0,47**	4.1178448492596e-12
CALM2	ENSG00000143933	**-0,68**	3.47715337791891e-27	**-0,21**	0.00303439391972829
CALM3	ENSG00000160014	**-0,14**	0.0504772536289049	**-0,04**	0.59804089178043
CAMK2A	ENSG00000070808	**0,04**	0.559925961380528	**0,03**	0.690815171680589
CAMK2B	ENSG00000058404	**0,74**	1.32581980065863e-34	**0,48**	2.01245174844293e-12
CAMK2D	ENSG00000145349	**-0,07**	0.308804696441233	**0,44**	1.26025680881935e-10
CAMK2G	ENSG00000148660	**0,09**	0.229968443341259	**0,56**	4.74561885427332e-17
CASP1	ENSG00000137752	**-0,37**	1.27731924197375e-07	**-0,28**	9.59804207795709e-05
CASP3	ENSG00000164305	**-0,44**	2.73282089423008e-10	**-0,27**	0.000118375769396683
CBL	ENSG00000110395	**-0,55**	3.46855474788441e-16	**0,05**	0.504839833517787
CCL11	ENSG00000172156	**0,00**	0.954269817554332	**-0,13**	0.0817787883638779
CCL2	ENSG00000108691	**-0,47**	6.41416189475132e-12	**-0,46**	1.41332859075084e-11
CCL20	ENSG00000115009	**-0,18**	0.0128253516225991	**-0,26**	0.000341703645504321
CCL21	ENSG00000137077	**-0,29**	4.65304645899121e-05	**-0,21**	0.00301162239899604

CCL22	ENSG00000102962	**-0,26**	0.000314023038841977	**-0,25**	0.000424401838374593
CCL26	ENSG00000006606	**0,00**	0.950115594817246	**0,20**	0.0066928053768366
CCL27	ENSG00000213927	**0,30**	1.90683482366933e-05	**0,02**	0.782201176780412
CCL28	ENSG00000151882	**-0,40**	1.35714015049185e-08	**-0,21**	0.00387088706530998
CCL7	ENSG00000108688	**-0,43**	3.93576890874292e-10	**-0,48**	3.56698113758084e-12
CCL8	ENSG00000108700	**-0,27**	0.000146778473349971	**-0,14**	0.0540654083282926
CCND1	ENSG00000110092	**-0,33**	4.00428196513867e-06	**-0,37**	1.33681641242413e-07
CCR1	ENSG00000163823	**-0,35**	5.08879003215275e-07	**0,04**	0.559399183849147
CCR10	ENSG00000184451	**0,61**	3.18812512187198e-21	**0,04**	0.594731985386306
CCR5	ENSG00000160791	**-0,38**	5.16932265268277e-08	**-0,03**	0.706147121741022
CCR6	ENSG00000112486	**-0,26**	0.000275987101459466	**0,05**	0.471190891314659
CCR7	ENSG00000126353	**-0,12**	0.105218799104109	**-0,29**	5.34596688565147e-05
CCR9	ENSG00000173585	**-0,15**	0.0440352197050433	**-0,25**	0.00045398895934604
CCRL2	ENSG00000121797	**-0,41**	2.98920790419968e-09	**-0,35**	7.00620400797777e-07
CD27	ENSG00000139193	**-0,02**	0.735132116826637	**0,18**	0.0153443478823606
CD36	ENSG00000135218	**-0,14**	0.0501483907875544	**0,33**	3.98276990022087e-06
CD4	ENSG00000010610	**-0,26**	0.00026842756662553	**0,09**	0.203957226172094
CD40	ENSG00000101017	**-0,22**	0.00245987679052234	**-0,27**	0.000173068923433882
CD44	ENSG00000026508	**-0,61**	3.86976825536918e-21	**-0,55**	8.21955012712848e-17
CD80	ENSG00000121594	**-0,09**	0.222628048127229	**-0,26**	0.000315313495242874
CD86	ENSG00000114013	**-0,45**	5.19997845189528e-11	**-0,22**	0.00274785207855291
CDKN1A	ENSG00000124762	**0,21**	0.00313834927732949	**-0,13**	0.0807422336973029
CEBPD	ENSG00000221869	**0,55**	1.46597502611609e-16	**-0,24**	0.000846135608802876
CHUK	ENSG00000213341	**-0,34**	1.1178268897534e-06	**0,20**	0.00625863331816267
CIITA	ENSG00000179583	**0,16**	0.0251925400006313	**0,05**	0.454992654836226
CISH	ENSG00000114737	**-0,04**	0.623436251380992	**-0,53**	1.83498073104994e-15
CLCF1	ENSG00000175505	**0,17**	0.0184926902909362	**-0,49**	9.04654029009691e-13
CNKSR1	ENSG00000142675	**0,49**	1.15206168368363e-12	**-0,21**	0.00299979158012222
CNKSR2	ENSG00000149970	**0,21**	0.00380327098192265	**0,46**	1.73974498159781e-11
CNTF	ENSG00000242689	**-0,38**	8.88862438427588e-08	**-0,15**	0.035450938097936
CNTFR	ENSG00000122756	**0,37**	2.04816568631161e-07	**-0,06**	0.411403060567298
COL1A2	ENSG00000164692	**0,06**	0.397584704617204	**0,20**	0.00601987398531924
CRK	ENSG00000167193	**-0,75**	8.05055390381393e-36	**-0,30**	2.82393030151967e-05
CRKL	ENSG00000099942	**-0,32**	4.76681209703592e-06	**0,30**	3.16490373461747e-05
CRLF1	ENSG00000006016	**0,64**	2.47341036668506e-23	**0,01**	0.89934932703081
CSF1	ENSG00000184371	**0,25**	0.000403824125722475	**-0,39**	2.98123865451969e-08
CSF1R	ENSG00000182578	**-0,18**	0.0137113305549515	**0,03**	0.704893184768505
CSF2	ENSG00000164400	**0,03**	0.681839701485133	**-0,33**	4.43750237595565e-06
CSF2RB	ENSG00000100368	**0,13**	0.0688725900856352	**0,05**	0.470530770022826
CSF3	ENSG00000108342	**0,18**	0.0154185750024194	**-0,36**	2.92319384299176e-07
CSF3R	ENSG00000119535	**-0,02**	0.827356498428349	**-0,05**	0.48260413478207
CSK	ENSG00000103653	**0,39**	3.39924160397455e-08	**-0,21**	0.00320616186254278
CTF1	ENSG00000150281	**0,06**	0.374255243047533	**-0,15**	0.0449285995603537
CUL1	ENSG00000055130	**-0,52**	2.14761458340907e-14	**-0,21**	0.00400553337582581
CUL3	ENSG00000036257	**-0,46**	2.56155369936478e-11	**0,11**	0.122519232267966
CXCL1	ENSG00000163739	**-0,21**	0.00367753408294341	**-0,41**	2.5377874669153e-09
CXCL10	ENSG00000169245	**-0,20**	0.00635049523593809	**-0,30**	1.90252887758536e-05
CXCL11	ENSG00000169248	**-0,25**	0.000407303894057176	**-0,23**	0.00109819780946129
CXCL12	ENSG00000107562	**-0,25**	0.000383990851726459	**-0,39**	2.10479387532965e-08
CXCL14	ENSG00000145824	**0,00**	0.980920224019042	**-0,30**	2.02427194060977e-05
CXCL16	ENSG00000161921	**-0,28**	0.000114844855785013	**-0,50**	2.06541890758647e-13
CXCL17	ENSG00000189377	**-0,40**	1.28106250427303e-08	**-0,42**	9.1866463267923e-10
CXCL2	ENSG00000081041	**-0,17**	0.0193860342280525	**-0,47**	7.45811154682921e-12
CXCL3	ENSG00000163734	**-0,13**	0.0751678973976666	**-0,53**	3.42505660121794e-15
CXCL5	ENSG00000163735	**-0,35**	9.93191522809374e-07	**-0,44**	1.84244199464914e-10
CXCL6	ENSG00000124875	**-0,23**	0.00166338197486167	**-0,19**	0.00773373521915048
CXCL8	ENSG00000169429	**-0,42**	1.12097774486885e-09	**-0,54**	1.55144253234339e-15
CXCL9	ENSG00000138755	**-0,21**	0.00288462294587905	**-0,21**	0.00355173584180992

CXCR4	ENSG00000121966	**-0,18**	0.0125262267219206	**0,03**	0.650330003763597
CXCR5	ENSG00000160683	**0,34**	1.14022885829554e-06	**-0,30**	2.11468012073142e-05
CXCR6	ENSG00000172215	**-0,23**	0.00109499009940793	**-0,15**	0.0406312147914688
DAB2IP	ENSG00000136848	**0,28**	7.61443521425966e-05	**-0,41**	3.6488594170386e-09
DDX58	ENSG00000107201	**-0,42**	9.47228472906936e-10	**-0,23**	0.00110291790660329
DLG4	ENSG00000132535	**0,54**	1.23149471987978e-15	**0,61**	1.18205184310589e-20
DUSP1	ENSG00000120129	**0,14**	0.0570076158581769	**-0,23**	0.00143559494779822
DUSP10	ENSG00000143507	**-0,38**	7.0817711486128e-08	**-0,32**	6.31271011761031e-06
DUSP16	ENSG00000111266	**-0,51**	5.02361833977153e-14	**-0,18**	0.0109236136489219
DUSP2	ENSG00000158050	**0,11**	0.133037817598459	**-0,37**	1.65423665672134e-07
DUSP4	ENSG00000120875	**-0,42**	2.32293611490365e-09	**-0,54**	1.09789203695076e-15
DUSP5	ENSG00000138166	**-0,30**	3.24551606542836e-05	**-0,40**	6.65653677018933e-09
DUSP6	ENSG00000139318	**-0,17**	0.0206498672669411	**-0,29**	5.71289633798277e-05
DUSP7	ENSG00000164086	**0,57**	4.05257135265869e-18	**0,03**	0.716312135057821
DUSP8	ENSG00000184545	**0,72**	1.58629569059562e-31	**0,11**	0.136548053720883
EDA	ENSG00000158813	**-0,38**	8.51774978205229e-08	**0,33**	2.3572475846563e-06
EDA2R	ENSG00000131080	**-0,04**	0.586812672901528	**0,21**	0.0043740962918577
EDAR	ENSG00000135960	**-0,39**	2.77433278130626e-08	**-0,30**	3.42712704108932e-05
EDARADD	ENSG00000186197	**0,11**	0.11663795231865	**0,71**	3.56169356825958e-31
EGF	ENSG00000138798	**-0,41**	5.7824858431494e-09	**-0,32**	8.98574152974385e-06
EGFR	ENSG00000146648	**-0,60**	4.3058782674096e-20	**-0,50**	1.1243564967149e-13
EGR1	ENSG00000120738	**-0,14**	0.0623343681152722	**-0,22**	0.00280085718660578
EIF2AK2	ENSG00000055332	**-0,46**	3.30817892243563e-11	**0,15**	0.0406389192141145
EIF4A1	ENSG00000161960	**-0,74**	2.31419729734339e-34	**-0,31**	1.32143630545985e-05
EIF4A2	ENSG00000156976	**-0,20**	0.00481290536003911	**0,30**	3.25275922543956e-05
EIF4A3	ENSG00000141543	**-0,58**	1.05148927620302e-18	**-0,20**	0.00611830274315773
EIF4E	ENSG00000151247	**-0,59**	2.91968333681233e-19	**-0,01**	0.913889836816078
EIF4E2	ENSG00000135930	**-0,75**	4.96274006533698e-36	**-0,19**	0.0101865175958272
EIF4E3	ENSG00000163412	**-0,40**	1.13331191181991e-08	**0,27**	0.000195718528081111
EIF4G1	ENSG00000114867	**-0,12**	0.100919174736973	**-0,10**	0.148598787003511
EIF4G2	ENSG00000110321	**-0,66**	1.63482213911667e-25	**-0,10**	0.149164948088084
EIF4G3	ENSG00000075151	**-0,18**	0.0108499286361439	**0,32**	7.98161501374028e-06
ERBB2	ENSG00000141736	**0,06**	0.409347835376235	**-0,38**	5.87054493238728e-08
ERBB3	ENSG00000065361	**-0,41**	4.66347012065983e-09	**-0,40**	1.01911321543803e-08
ERBB4	ENSG00000178568	**-0,17**	0.0211733740474937	**0,35**	7.20541999998462e-07
EREG	ENSG00000124882	**-0,40**	1.23180034589255e-08	**-0,28**	9.85874900087042e-05
F13A1	ENSG00000124491	**-0,13**	0.0726193017982728	**0,47**	1.08277496324921e-11
FBXW11	ENSG00000072803	**-0,34**	1.52499677719491e-06	**0,30**	2.63687140639665e-05
FCGR1A	ENSG00000150337	**-0,16**	0.0235144840293745	**-0,06**	0.432421826844612
FGA	ENSG00000171560	**-0,31**	1.56959891589665e-05	**-0,31**	1.34666094371246e-05
FGB	ENSG00000171564	**-0,25**	0.000378372380822799	**-0,19**	0.0080915117011165
FGF1	ENSG00000113578	**-0,44**	2.44066913828584e-10	**0,02**	0.754378968271158
FGF10	ENSG00000070193	**-0,55**	2.46422122616766e-16	**-0,36**	2.34550805683999e-07
FGF17	ENSG00000158815	**0,56**	4.12854930573952e-17	**0,03**	0.721583744842591
FGF18	ENSG00000156427	**0,45**	6.02265824835725e-11	**0,03**	0.703577358408286
FGF19	ENSG00000162344	**0,00**	0.974288497012945	**-0,05**	0.511373306168205
FGF2	ENSG00000138685	**-0,46**	3.48955204221941e-11	**-0,41**	2.42795689103835e-09
FGF20	ENSG00000078579	**0,06**	0.405200564211745	**-0,06**	0.434892270569
FGF5	ENSG00000138675	**-0,34**	1.10199987998084e-06	**-0,12**	0.110867479809079
FGF7	ENSG00000140285	**-0,34**	1.68361060791868e-06	**-0,23**	0.00144647655547454
FGF9	ENSG00000102678	**0,19**	0.00714864285943579	**0,64**	1.37001729738829e-23
FGFR1	ENSG00000077782	**0,22**	0.00183201446032256	**0,06**	0.394943323990646
FGFR2	ENSG00000066468	**-0,42**	1.04165032592503e-09	**-0,29**	5.36124292340821e-05
FGFR3	ENSG00000068078	**0,28**	8.57267080027185e-05	**-0,32**	7.7194370713869e-06
FGFR4	ENSG00000160867	**0,52**	1.62775920503534e-14	**-0,18**	0.0145844884117514
FGG	ENSG00000171557	**-0,29**	3.76327983815819e-05	**-0,32**	8.44356327212361e-06
FLNB	ENSG00000136068	**-0,43**	6.23983462640779e-10	**0,02**	0.793107892842371
FLT3	ENSG00000122025	**-0,38**	4.27464393275612e-08	**-0,42**	1.62298966237945e-09

FLT3LG	ENSG00000090554	**0,62**	5.53901359587075e-22	**-0,08**	0.275421422615208
FN1	ENSG00000115414	**-0,10**	0.148416945251089	**0,15**	0.0355965389095339
FOS	ENSG00000170345	**0,07**	0.319430866802173	**-0,10**	0.171969344062599
FOXO1	ENSG00000150907	**-0,09**	0.224808866156479	**0,51**	2.87947047397879e-14
FOXO3	ENSG00000118689	**-0,51**	3.26633890967499e-14	**-0,28**	0.000115347596844021
FPR1	ENSG00000171051	**-0,29**	4.06826197094733e-05	**-0,04**	0.564032146368334
FRS2	ENSG00000166225	**-0,44**	1.88320491524232e-10	**0,14**	0.0500883303385876
FRS3	ENSG00000137218	**0,66**	2.85439463413742e-25	**0,03**	0.694289602025594
FSCN1	ENSG00000075618	**0,67**	3.42132863793156e-26	**0,03**	0.714942547253353
FYN	ENSG00000010810	**-0,11**	0.115624618811205	**0,36**	4.65577043086155e-07
GAB2	ENSG00000033327	**-0,34**	1.31467093679976e-06	**-0,07**	0.367957087429214
GATA3	ENSG00000107485	**0,07**	0.316648697971256	**-0,19**	0.0069102554727463
GBP1	ENSG00000117228	**-0,54**	3.72749092694847e-16	**-0,39**	1.62642204239511e-08
GBP2	ENSG00000162645	**-0,48**	2.60404210434064e-12	**-0,48**	2.35061797063963e-12
GBP3	ENSG00000117226	**-0,33**	3.50073784638969e-06	**-0,26**	0.000333079671380302
GBP4	ENSG00000162654	**-0,20**	0.00599815698168757	**-0,01**	0.87584477265925
GBP5	ENSG00000154451	**-0,32**	8.76934311592565e-06	**-0,24**	0.000648027424968863
GDNF	ENSG00000168621	**-0,17**	0.0163307015225077	**-0,13**	0.0772094121907166
GFRA1	ENSG00000151892	**-0,38**	6.71371640309965e-08	**0,16**	0.0302918587636134
GFRA2	ENSG00000168546	**0,00**	0.970842449324579	**-0,13**	0.0820891720087893
GFRA3	ENSG00000146013	**-0,19**	0.00819200193096893	**-0,29**	5.35970964473786e-05
GHR	ENSG00000112964	**-0,32**	8.68237515685064e-06	**0,29**	6.13402030775807e-05
GRB2	ENSG00000177885	**-0,33**	4.11133454298968e-06	**0,00**	0.988232456850839
GRIN1	ENSG00000176884	**0,53**	2.1166464702785e-15	**0,21**	0.00357098649132247
GRIN2A	ENSG00000183454	**-0,08**	0.261036892101587	**0,38**	7.441138253032e-08
GRIN2C	ENSG00000161509	**0,28**	0.000100751990510586	**-0,05**	0.492181515814096
GRIN2D	ENSG00000105464	**0,60**	5.68511513929615e-20	**0,19**	0.00965993608863983
HAVCR2	ENSG00000135077	**-0,30**	3.37417972950292e-05	**-0,12**	0.096150386924099
HBEGF	ENSG00000113070	**-0,39**	3.46525375311741e-08	**-0,33**	2.64354643526928e-06
HCK	ENSG00000101336	**-0,24**	0.000667377904420875	**-0,03**	0.634449615819388
HERC5	ENSG00000138646	**0,05**	0.506459355392549	**0,50**	2.87793406986351e-13
HGF	ENSG00000019991	**-0,30**	1.93213994726155e-05	**-0,09**	0.214792319155701
HIF1A	ENSG00000100644	**-0,57**	1.31809194555056e-17	**-0,30**	2.94551704669528e-05
HIST1H3D	ENSG00000197409	**0,43**	6.94604895681224e-10	**-0,15**	0.0407650591691055
HLA-A	ENSG00000206503	**0,41**	3.17676782898581e-09	**0,05**	0.501993587554691
HLA-B	ENSG00000234745	**-0,07**	0.321245752822301	**-0,37**	1.35154815601213e-07
HLA-C	ENSG00000204525	**0,22**	0.00177092256548731	**-0,05**	0.493859111819826
HLA-DPA1	ENSG00000231389	**-0,33**	3.48390737616717e-06	**-0,14**	0.0625700888034012
HLA-DPB1	ENSG00000223865	**-0,14**	0.0500989017843944	**-0,29**	6.04620119758697e-05
HLA-DQA1	ENSG00000196735	**-0,35**	7.32409952502228e-07	**-0,06**	0.395299635736331
HLA-DQB1	ENSG00000179344	**-0,16**	0.0281057297508137	**-0,09**	0.220240452128911
HLA-DRA	ENSG00000204287	**-0,49**	9.0078408324313e-13	**-0,17**	0.0198061579232227
HLA-DRB1	ENSG00000196126	**-0,34**	1.84758362592658e-06	**-0,14**	0.0539708285037983
HLA-DRB5	ENSG00000198502	**-0,22**	0.00268285198387145	**-0,11**	0.127383172104309
HLA-E	ENSG00000204592	**0,16**	0.0291932833068203	**-0,28**	9.86111154029346e-05
HLA-F	ENSG00000204642	**0,48**	3.0844601875437e-12	**-0,25**	0.000591139503654884
HLA-G	ENSG00000204632	**0,05**	0.482261735708689	**-0,31**	9.9969053922563e-06
HLA-H	ENSG00000206341	**0,18**	0.0140794221492256	**0,09**	0.239411023029804
HMOX1	ENSG00000100292	**0,09**	0.201600022912768	**-0,07**	0.326888502930445
HRAS	ENSG00000174775	**0,55**	2.46001885119236e-16	**0,06**	0.405352115542166
HSP90AA1	ENSG00000080824	**-0,39**	3.46285886000608e-08	**0,10**	0.167349914358279
HSP90B1	ENSG00000166598	**-0,30**	2.46119325311968e-05	**0,00**	0.96229049370025
HSPA8	ENSG00000109971	**-0,53**	3.69698672886195e-15	**0,05**	0.483995357792431
ICAM1	ENSG00000090339	**-0,26**	0.000310326558739677	**-0,38**	7.74971946141322e-08
IFI27	ENSG00000165949	**0,06**	0.417463327642741	**-0,18**	0.012460364710499
IFI30	ENSG00000216490	**0,08**	0.295313404702536	**-0,12**	0.110463236495231
IFI35	ENSG00000068079	**0,08**	0.291372865987342	**0,02**	0.766252150166224
IFI6	ENSG00000126709	**0,60**	2.28537513168798e-20	**0,14**	0.0534002297273771

IFIT1	ENSG00000185745		**-0,10**	0.168179329055682		**0,13**	0.0790116548077697
IFIT2	ENSG00000119922		**-0,39**	1.81926495916762e-08		**-0,37**	1.77032737388667e-07
IFIT3	ENSG00000119917		**-0,46**	1.83893380809121e-11		**-0,36**	2.66527425198204e-07
IFITM1	ENSG00000185885		**-0,12**	0.0868732507529924		**-0,10**	0.182545759924403
IFITM2	ENSG00000185201		**-0,08**	0.259989669232281		**-0,40**	6.3595868417778e-09
IFITM3	ENSG00000142089		**0,18**	0.0128806254229862		**-0,45**	4.72199476985729e-11
IFNAR1	ENSG00000142166		**-0,63**	1.93927974544317e-22		**-0,26**	0.000304375990188567
IFNAR2	ENSG00000159110		**-0,66**	1.12799580148387e-25		**-0,42**	1.9875301607214e-09
IFNGR1	ENSG00000027697		**-0,61**	9.20222557434055e-21		**-0,47**	6.89646309761597e-12
IFNGR2	ENSG00000159128		**-0,51**	2.75065468913454e-14		**-0,54**	4.3989143154181e-16
IFNLR1	ENSG00000185436		**-0,31**	1.556330238438e-05		**-0,13**	0.0754043913196151
IGHG1	ENSG00000211896		**-0,01**	0.877749134991903		**-0,14**	0.0566350368286664
IKBKB	ENSG00000104365		**0,00**	0.955333101431861		**-0,29**	6.32388919004456e-05
IL10	ENSG00000136634		**-0,35**	6.62961598191475e-07		**-0,14**	0.0481751827565998
IL10RA	ENSG00000110324		**0,18**	0.0125206546242096		**0,57**	3.4786531533015e-18
IL10RB	ENSG00000243646		**-0,42**	9.56550701097362e-10		**-0,38**	5.57365766134934e-08
IL11	ENSG00000095752		**-0,07**	0.365283087770948		**-0,32**	7.52132567074106e-06
IL11RA	ENSG00000137070		**0,39**	1.96444396848656e-08		**-0,07**	0.319166387616187
IL12A	ENSG00000168811		**0,00**	0.991110726408829		**-0,38**	7.9570963243035e-08
IL12RB1	ENSG00000096996		**0,23**	0.00122230356369201		**0,15**	0.0390269569523579
IL12RB2	ENSG00000081985		**-0,33**	2.71023299394142e-06		**-0,30**	1.96597294538937e-05
IL13RA1	ENSG00000131724		**-0,53**	3.74050990258939e-15		**-0,02**	0.760937217074865
IL13RA2	ENSG00000123496		**-0,31**	1.25502366462693e-05		**-0,18**	0.0109951027134327
IL15	ENSG00000164136		**-0,22**	0.00267988795387774		**-0,70**	6.9321397775139e-29
IL15RA	ENSG00000134470		**0,02**	0.776052277699761		**-0,58**	2.89052913197342e-18
IL16	ENSG00000172349		**-0,06**	0.424249139118085		**-0,01**	0.857373004345726
IL17D	ENSG00000172458		**0,35**	6.02171551746405e-07		**0,30**	1.98213111798617e-05
IL17RA	ENSG00000177663		**-0,08**	0.258794478145161		**0,12**	0.0878496665060153
IL17RB	ENSG00000056736		**0,26**	0.000214422555033613		**0,01**	0.922246113753298
IL17RC	ENSG00000163702		**0,71**	2.46898296774402e-30		**0,10**	0.188557956787351
IL17RD	ENSG00000144730		**-0,51**	3.5797286312822e-14		**-0,37**	1.06999737368533e-07
IL17RE	ENSG00000163701		**0,10**	0.187447665640803		**-0,03**	0.714042088606822
IL18	ENSG00000150782		**-0,27**	0.000193778534666499		**-0,32**	4.62993766015631e-06
IL18BP	ENSG00000137496		**0,66**	1.94215147758583e-25		**-0,03**	0.668847876969088
IL18R1	ENSG00000115604		**-0,14**	0.0532949380420931		**-0,29**	3.72886234460458e-05
IL18RAP	ENSG00000115607		**0,03**	0.73091966474103		**-0,32**	6.22477610834922e-06
IL1A	ENSG00000115008		**-0,32**	8.45224131569533e-06		**-0,30**	2.66784939150809e-05
IL1B	ENSG00000125538		**-0,35**	7.39860946753494e-07		**-0,35**	8.80488246544108e-07
IL1R1	ENSG00000115594		**-0,54**	1.42662150131136e-15		**-0,47**	7.81353200584664e-12
IL1R2	ENSG00000115590		**-0,24**	0.000893563792501214		**-0,37**	1.98110558437503e-07
IL1RAP	ENSG00000196083		**-0,55**	2.20439359523922e-16		**-0,41**	3.36281933133914e-09
IL1RL1	ENSG00000115602		**-0,22**	0.00213247658506211		**-0,23**	0.00114694442430058
IL1RL2	ENSG00000115598		**-0,33**	2.81793629147517e-06		**0,00**	0.998551812062902
IL1RN	ENSG00000136689		**-0,28**	7.23573823279185e-05		**-0,34**	1.91738457528817e-06
IL20RA	ENSG00000016402		**0,09**	0.193573144428191		**0,41**	3.82020705217257e-09
IL20RB	ENSG00000174564		**0,01**	0.867333638448443		**-0,22**	0.0023392089228126
IL21R	ENSG00000103522		**0,37**	1.34228519291631e-07		**0,06**	0.427981985859474
IL22RA1	ENSG00000142677		**-0,30**	3.08367034022956e-05		**-0,48**	2.87726914589897e-12
IL23A	ENSG00000110944		**-0,03**	0.644645797902959		**-0,45**	9.67115060054828e-11
IL23R	ENSG00000162594		**-0,22**	0.00257454489699698		**0,22**	0.00182712979345897
IL24	ENSG00000162892		**-0,29**	3.79070970091223e-05		**-0,17**	0.0192959023633358
IL27RA	ENSG00000104998		**-0,10**	0.162994150074083		**-0,40**	8.86323974097409e-09
IL2RB	ENSG00000100385		**-0,18**	0.0144431332335081		**-0,26**	0.000343963819283761
IL2RG	ENSG00000147168		**-0,40**	7.15846989614708e-09		**-0,24**	0.000732077598678967
IL31RA	ENSG00000164509		**-0,29**	5.37242335659899e-05		**-0,16**	0.0259961120033516
IL32	ENSG00000008517		**-0,18**	0.010515760811482		**-0,42**	2.06121281580945e-09
IL33	ENSG00000137033		**-0,39**	3.03371256792028e-08		**-0,42**	9.27237350542814e-10
IL34	ENSG00000157368		**0,28**	9.03762598218732e-05		**-0,16**	0.0261104425619999

IL4I1	ENSG00000104951	**0,36**	3.7034875200183e-07	**0,00**	0.976900621332181
IL4R	ENSG00000077238	**-0,24**	0.000698199612902479	**-0,60**	9.01546609244505e-20
IL5RA	ENSG00000091181	**0,03**	0.700688253503954	**0,18**	0.0131308852385535
IL6	ENSG00000136244	**-0,38**	6.23851661010328e-08	**-0,54**	1.04547716421021e-15
IL6R	ENSG00000160712	**0,24**	0.00098114997789636	**0,36**	3.88431360002777e-07
IL6ST	ENSG00000134352	**-0,75**	3.44003434019804e-35	**-0,27**	0.000126298974719615
IL6STP1	ENSG00000227018	**0,22**	0.00182667155608608	**-0,05**	0.467673423794732
IL7	ENSG00000104432	**-0,13**	0.0689954954170196	**-0,11**	0.125212694452644
IL7R	ENSG00000168685	**-0,39**	2.05832329971e-08	**-0,28**	9.30815288694941e-05
ILDR1	ENSG00000145103	**-0,26**	0.000339162859106476	**-0,17**	0.0212016910487067
ILDR2	ENSG00000143195	**0,16**	0.0255808337937447	**0,63**	1.23206894912374e-22
ILF2	ENSG00000143621	**-0,72**	3.39653681088795e-32	**-0,15**	0.0421147591165147
ILF3	ENSG00000129351	**0,27**	0.000177465020068769	**-0,16**	0.0284876796290236
ILF3-AS1	ENSG00000267100	**0,29**	3.90272388294899e-05	**0,24**	0.00104592585053249
ILK	ENSG00000166333	**-0,54**	4.93156949548619e-16	**-0,14**	0.0624471459452703
ILKAP	ENSG00000132323	**0,36**	2.4144066299645e-07	**-0,17**	0.0198846575309082
ILVBL	ENSG00000105135	**0,66**	2.61039099230088e-25	**-0,04**	0.592512226701362
INPP5D	ENSG00000168918	**-0,03**	0.680907530145568	**0,13**	0.0686788270498966
INPPL1	ENSG00000165458	**0,40**	8.76495288213761e-09	**-0,03**	0.68995585681913
IP6K2	ENSG00000068745	**0,30**	2.25135147047044e-05	**0,07**	0.324641430677502
IQGAP1	ENSG00000140575	**-0,53**	3.21739049189942e-15	**0,02**	0.784677937699159
IRAK1	ENSG00000184216	**0,50**	2.20514607971888e-13	**-0,12**	0.103457118518497
IRAK2	ENSG00000134070	**-0,35**	6.95029660032743e-07	**-0,43**	7.01973501615173e-10
IRAK3	ENSG00000090376	**-0,39**	2.47592964601667e-08	**-0,13**	0.0726301846659023
IRAK4	ENSG00000198001	**-0,47**	5.89109885371024e-12	**-0,07**	0.309402333728931
IRF1	ENSG00000125347	**-0,22**	0.00218498430516907	**-0,45**	6.8595246836266e-11
IRF2	ENSG00000168310	**-0,53**	2.64551578741172e-15	**-0,12**	0.0860556588295567
IRF3	ENSG00000126456	**0,62**	1.55820327947092e-21	**-0,13**	0.0674161842953076
IRF4	ENSG00000137265	**-0,37**	1.1784521145191e-07	**-0,50**	3.2774625407313e-13
IRF5	ENSG00000128604	**0,52**	6.57680600283766e-15	**-0,13**	0.0848245154461347
IRF6	ENSG00000117595	**-0,56**	4.31345058997722e-17	**0,07**	0.317195808715754
IRF7	ENSG00000185507	**0,66**	2.2146313704963e-25	**-0,12**	0.108319133878486
IRF8	ENSG00000140968	**-0,17**	0.0189482704504927	**-0,20**	0.00606151359148182
IRF9	ENSG00000213928	**-0,01**	0.927355270888246	**-0,11**	0.13027341850353
IRS1	ENSG00000169047	**0,19**	0.00779396677946712	**-0,36**	2.89957043440865e-07
IRS2	ENSG00000185950	**0,26**	0.000226329855985828	**0,23**	0.00164737210953704
ISG15	ENSG00000187608	**0,61**	3.31669997958626e-21	**-0,13**	0.0836352099427672
ISG20	ENSG00000172183	**0,40**	1.36808822637563e-08	**-0,27**	0.000146216702121402
ITGA2B	ENSG00000005961	**0,36**	3.40895296839718e-07	**-0,07**	0.360035315947675
ITGAM	ENSG00000169896	**-0,25**	0.000595098079308897	**-0,39**	3.24725303031618e-08
ITGAX	ENSG00000140678	**-0,10**	0.184469724830344	**-0,31**	1.00793506347633e-05
ITGB1	ENSG00000150093	**-0,70**	1.15592737610596e-29	**-0,30**	2.34315958300453e-05
ITGB2	ENSG00000160255	**0,11**	0.115161320399373	**-0,07**	0.302669643195739
ITGB3	ENSG00000259207	**-0,26**	0.000233543787175904	**0,19**	0.00963725695924336
JAK1	ENSG00000162434	**-0,52**	1.21965899014069e-14	**-0,17**	0.0181285138677532
JAK2	ENSG00000096968	**-0,21**	0.00293620050676593	**0,31**	1.15950395778076e-05
JAK3	ENSG00000105639	**0,16**	0.0254759799124599	**-0,12**	0.10278525129845
JUNB	ENSG00000171223	**0,64**	7.67212510391768e-24	**-0,12**	0.102603877780216
KBTBD7	ENSG00000120696	**0,00**	0.986803472005442	**0,57**	4.26627095608914e-18
KIT	ENSG00000157404	**-0,32**	6.06911145992919e-06	**-0,14**	0.0548835554504869
KITLG	ENSG00000049130	**-0,59**	2.75247625001181e-19	**-0,24**	0.000939067200012976
KL	ENSG00000133116	**-0,09**	0.206308778408487	**0,56**	2.74952829375224e-17
KLB	ENSG00000134962	**-0,14**	0.0588452193828641	**0,15**	0.0368329413312485
KPNA1	ENSG00000114030	**-0,56**	2.77776469206779e-17	**0,04**	0.59372479604011
KPNA2	ENSG00000182481	**-0,61**	7.6938482526533e-21	**-0,08**	0.286337600973617
KPNA3	ENSG00000102753	**-0,60**	6.51895522566435e-20	**-0,15**	0.0332375772025721
KPNA4	ENSG00000186432	**-0,55**	1.12461451997208e-16	**-0,26**	0.000223376112058276
KPNA5	ENSG00000196911	**0,15**	0.0416289471735843	**0,42**	1.22578310365348e-09

KPNA7	ENSG00000185467	**-0,32**	7.63486624903092e-06	**-0,46**	1.44370295296161e-11
KPNB1	ENSG00000108424	**-0,69**	1.63870755330394e-28	**0,00**	0.951373334868211
KRAS	ENSG00000133703	**-0,67**	5.23274226421398e-26	**-0,14**	0.0467193743947844
KSR1	ENSG00000141068	**-0,10**	0.170705294008302	**-0,09**	0.197240330825575
KSR2	ENSG00000171435	**-0,11**	0.13610262226458	**0,57**	4.82254697579913e-18
LAMA5	ENSG00000130702	**0,70**	1.89977734364957e-29	**-0,04**	0.619628551027396
LAMTOR2	ENSG00000116586	**0,07**	0.30320300443563	**-0,19**	0.00783042584098862
LAMTOR3	ENSG00000109270	**-0,48**	3.19905307693503e-12	**0,06**	0.44713511686235
LAT	ENSG00000213658	**0,43**	3.55290622637628e-10	**-0,19**	0.00780893729859522
LBP	ENSG00000129988	**-0,31**	1.41177897209494e-05	**-0,36**	4.62292072031325e-07
LCK	ENSG00000182866	**-0,15**	0.0353462625797426	**-0,23**	0.00136232130345569
LCN2	ENSG00000148346	**-0,02**	0.78839820497184	**-0,43**	8.31216146093399e-10
LGALS9	ENSG00000168961	**-0,24**	0.000649493932591455	**-0,41**	6.19081511091341e-09
LIF	ENSG00000128342	**-0,05**	0.482819781309866	**-0,45**	6.38650230162e-11
LIFR	ENSG00000113594	**-0,64**	2.66231348605376e-23	**-0,42**	1.55726367986194e-09
LTA	ENSG00000226979	**0,11**	0.115156450378494	**-0,07**	0.32644901266702
LTB	ENSG00000227507	**0,23**	0.00158753042490447	**-0,20**	0.00452746806166365
LTBR	ENSG00000111321	**0,47**	7.46962476537229e-12	**-0,15**	0.0394906107635093
LYN	ENSG00000254087	**-0,60**	9.33987560128866e-20	**-0,54**	1.34097702010841e-15
MAOA	ENSG00000189221	**-0,46**	1.69661508782793e-11	**0,04**	0.540401251319004
MAP2K1	ENSG00000169032	**-0,42**	1.0089549299884e-09	**-0,13**	0.0641132819324937
MAP2K2	ENSG00000126934	**0,66**	1.17674104864783e-25	**0,00**	0.958168479966848
MAP2K4	ENSG00000065559	**-0,62**	1.34976778554994e-21	**-0,07**	0.364097650238115
MAP2K6	ENSG00000108984	**-0,40**	1.53752344947262e-08	**0,19**	0.00843758230584174
MAP3K11	ENSG00000173327	**0,64**	3.91960507895763e-23	**-0,08**	0.276514101965175
MAP3K14	ENSG00000006062	**0,46**	1.93455883792861e-11	**-0,23**	0.00161677069109988
MAP3K3	ENSG00000198909	**0,19**	0.00723584433967179	**-0,24**	0.00070182737795194
MAP3K7	ENSG00000135341	**-0,35**	6.39003437413233e-07	**-0,01**	0.867092351786451
MAP3K8	ENSG00000107968	**-0,24**	0.000771602514091049	**-0,43**	5.60752233400665e-10
MAPK1	ENSG00000100030	**-0,60**	2.99200936810869e-20	**0,08**	0.251282465884628
MAPK3	ENSG00000102882	**0,36**	2.87917061872028e-07	**0,30**	2.0009180219269e-05
MARK3	ENSG00000075413	**-0,52**	1.00512518326643e-14	**-0,31**	1.59263964646641e-05
MCL1	ENSG00000143384	**-0,62**	2.55283843795004e-21	**-0,52**	1.22181414236806e-14
MET	ENSG00000105976	**-0,51**	3.38581637335033e-14	**-0,45**	6.2958833509714e-11
MID1	ENSG00000101871	**-0,53**	1.98427652752285e-15	**-0,43**	4.58300749788245e-10
MMP1	ENSG00000196611	**-0,42**	1.24415407656481e-09	**-0,28**	7.22680920601027e-05
MMP2	ENSG00000087245	**-0,04**	0.566889346600683	**-0,12**	0.086433619869771
MMP3	ENSG00000149968	**-0,31**	1.34277874227605e-05	**-0,29**	5.77960404913741e-05
MMP9	ENSG00000100985	**0,37**	1.19306251744215e-07	**0,00**	0.959604944654703
MT2A	ENSG00000125148	**0,58**	1.71821744569472e-18	**-0,14**	0.049420713386341
MUC1	ENSG00000185499	**-0,15**	0.0355048427511423	**-0,36**	4.62149623156673e-07
MX1	ENSG00000157601	**-0,18**	0.015182873912748	**-0,26**	0.000298574134706061
MX2	ENSG00000183486	**-0,20**	0.00461039956638195	**-0,20**	0.0065061668848554
MYC	ENSG00000136997	**-0,17**	0.0168645538571789	**-0,49**	3.93334794486251e-13
MYD88	ENSG00000172936	**-0,63**	1.30792485750974e-22	**-0,47**	7.90972736361841e-12
NCAM1	ENSG00000149294	**-0,05**	0.490859806182678	**0,47**	1.21484470358627e-11
NDC1	ENSG00000058804	**-0,66**	4.09127682020578e-25	**-0,26**	0.000335425175741885
NDN	ENSG00000182636	**0,70**	4.80593322642305e-29	**0,48**	1.44946504495289e-12
NEDD4	ENSG00000069869	**-0,66**	2.13251434405689e-25	**-0,26**	0.000313731354862089
NF1	ENSG00000196712	**-0,39**	2.28684008877139e-08	**0,20**	0.00551045757259773
NFKB1	ENSG00000109320	**-0,59**	1.41337345847154e-19	**-0,40**	1.27178951836757e-08
NFKB2	ENSG00000077150	**0,22**	0.00270211457539767	**-0,42**	1.95754158193685e-09
NOD1	ENSG00000106100	**0,31**	1.28556623781801e-05	**-0,16**	0.0233950677883959
NOD2	ENSG00000167207	**-0,16**	0.0271555303549858	**-0,40**	1.19593954178795e-08
NOS2	ENSG00000007171	**0,04**	0.553775969547528	**-0,01**	0.849052646270222
NRAS	ENSG00000213281	**-0,72**	2.4429538510366e-32	**-0,37**	2.07769539640877e-07
NRG1	ENSG00000157168	**-0,22**	0.00223620687201368	**0,10**	0.188268758914819
NRG2	ENSG00000158458	**0,61**	2.94648449444351e-21	**0,19**	0.01011891365336

NRG3	ENSG00000185737	**0,06**	0.38644227058143	**0,21**	0.00352491287516225
NRG4	ENSG00000169752	**-0,33**	3.71305767243e-06	**-0,38**	5.18476130305131e-08
NRTN	ENSG00000171119	**0,56**	3.79973082258011e-17	**-0,16**	0.0255635048195903
NUP107	ENSG00000111581	**-0,62**	9.43538460457257e-22	**-0,25**	0.000494824408482587
NUP133	ENSG00000069248	**-0,42**	2.16965760109799e-09	**0,23**	0.00111830145709892
NUP153	ENSG00000124789	**-0,54**	4.82339383706369e-16	**-0,02**	0.7920089123163
NUP155	ENSG00000113569	**-0,61**	5.81849116112152e-21	**0,07**	0.367715895286642
NUP160	ENSG00000030066	**-0,63**	3.28564870516045e-22	**-0,12**	0.103083038929497
NUP188	ENSG00000095319	**-0,40**	6.23734467188947e-09	**0,08**	0.269944093112653
NUP205	ENSG00000155561	**-0,49**	6.05373792187457e-13	**0,18**	0.0107606160356692
NUP210	ENSG00000132182	**0,52**	1.56145423902717e-14	**0,43**	5.11198069651801e-10
NUP214	ENSG00000126883	**-0,48**	1.53136965319655e-12	**0,09**	0.210719310211561
NUP35	ENSG00000163002	**-0,63**	2.4225536094197e-22	**-0,13**	0.0654848141450346
NUP37	ENSG00000075188	**-0,62**	5.57337639243015e-22	**-0,13**	0.0788255914557274
NUP43	ENSG00000120253	**-0,46**	2.05722737722958e-11	**-0,04**	0.617820075165719
NUP50	ENSG00000093000	**-0,64**	8.1042268834265e-24	**-0,08**	0.301341611027481
NUP54	ENSG00000138750	**-0,37**	1.31410703669273e-07	**-0,42**	2.34668204893079e-09
NUP58	ENSG00000139496	**-0,41**	5.95306995962975e-09	**0,04**	0.542416341467262
NUP62	ENSG00000213024	**0,37**	1.36548541348522e-07	**0,10**	0.175080687082358
NUP85	ENSG00000125450	**-0,23**	0.0013597614869025	**-0,31**	1.71806150412982e-05
NUP88	ENSG00000108559	**-0,58**	2.17069448206945e-18	**-0,17**	0.0188588087895462
NUP93	ENSG00000102900	**-0,60**	2.71805834933065e-20	**-0,17**	0.0161207349946931
NUP98	ENSG00000110713	**-0,57**	9.18879869168056e-18	**0,08**	0.278583444628742
NUPL2	ENSG00000136243	**-0,18**	0.0113063582261728	**0,02**	0.785220701246807
OAS1	ENSG00000089127	**-0,15**	0.0328201484234877	**0,05**	0.480262960349265
OAS2	ENSG00000111335	**-0,24**	0.000864956449482526	**-0,08**	0.252654097541656
OAS3	ENSG00000111331	**-0,26**	0.000330722148169071	**-0,14**	0.0474655004707886
OASL	ENSG00000135114	**-0,28**	0.000104403949329355	**-0,29**	3.71092703163127e-05
OPRD1	ENSG00000116329	**0,78**	1.54169413849143e-40	**0,28**	7.23468169245944e-05
OPRM1	ENSG00000112038	**0,02**	0.759475644215202	**0,16**	0.0291026114821989
OSM	ENSG00000099985	**0,24**	0.000652799939162351	**-0,25**	0.000431003212695394
OSMR	ENSG00000145623	**-0,65**	2.03919984970206e-24	**-0,60**	7.58445286554061e-20
PAQR3	ENSG00000163291	**-0,49**	5.41468657538426e-13	**-0,07**	0.340281542559996
PDGFA	ENSG00000197461	**0,04**	0.626089165063433	**-0,45**	4.13858779805118e-11
PDGFB	ENSG00000100311	**0,19**	0.0081006964117123	**-0,15**	0.0411937918306799
PDGFRA	ENSG00000134853	**-0,34**	1.69482491096601e-06	**-0,17**	0.0201033963368846
PDGFRB	ENSG00000113721	**0,02**	0.827196848427399	**0,19**	0.00836832573983567
PEA15	ENSG00000162734	**-0,65**	1.64023752354094e-24	**-0,27**	0.000202666382510082
PEBP1	ENSG00000089220	**0,25**	0.000516161877985369	**0,46**	2.02154583339568e-11
PELI1	ENSG00000197329	**-0,38**	4.34016196806095e-08	**-0,13**	0.0785560525101174
PELI2	ENSG00000139946	**-0,33**	3.06310669946723e-06	**-0,24**	0.000705477624879387
PELI3	ENSG00000174516	**0,83**	1.58777613151634e-49	**0,50**	2.80364309678431e-13
PHB	ENSG00000167085	**-0,52**	7.11281114075435e-15	**-0,12**	0.102923764522995
PIAS1	ENSG00000033800	**-0,28**	9.55460502211832e-05	**0,38**	4.92018850220099e-08
PIK3CA	ENSG00000121879	**-0,67**	2.39529234700326e-26	**-0,17**	0.0210010819676031
PIK3CB	ENSG00000051382	**-0,63**	4.05405996223875e-22	**-0,20**	0.00456660588519594
PIK3CD	ENSG00000171608	**0,29**	5.85616427793295e-05	**-0,34**	1.86148637990212e-06
PIK3R1	ENSG00000145675	**-0,70**	4.94075866857068e-29	**-0,09**	0.19404408185396
PIK3R2	ENSG00000105647	**0,77**	6.52792398222713e-39	**0,42**	2.10515977066689e-09
PIK3R3	ENSG00000117461	**-0,05**	0.526593151012579	**0,55**	1.10202969066579e-16
PIM1	ENSG00000137193	**-0,17**	0.0224194258620482	**-0,53**	1.66038013424789e-15
PIN1	ENSG00000127445	**0,73**	1.91107049191611e-33	**0,10**	0.154176054860317
PLCG1	ENSG00000124181	**0,23**	0.00140045220331286	**0,09**	0.230714922547303
PML	ENSG00000140464	**0,61**	1.82685940214907e-20	**-0,07**	0.305318714987008
POM121	ENSG00000196313	**0,26**	0.000299840881021032	**0,10**	0.153894421241205
POMC	ENSG00000115138	**0,70**	3.84337161142992e-29	**0,00**	0.950619597990398
POU2F1	ENSG00000143190	**-0,27**	0.000176383941602508	**0,14**	0.0505151537066798
PPM1B	ENSG00000138032	**-0,45**	7.49294356710572e-11	**0,01**	0.896168314644143

PPP2CA	ENSG00000113575	**-0,41**	2.75800443762301e-09	**-0,09**	0.232332134389138
PPP2CB	ENSG00000104695	**-0,29**	5.60647540573015e-05	**0,32**	8.87827848932038e-06
PPP2R1A	ENSG00000105568	**0,21**	0.00411510699179665	**-0,04**	0.595479890872361
PPP2R1B	ENSG00000137713	**-0,56**	2.64266607345147e-17	**0,07**	0.329253344289962
PPP2R5A	ENSG00000066027	**-0,13**	0.0704977300686071	**-0,25**	0.000623853979771191
PPP2R5B	ENSG00000068971	**0,64**	5.1323842640375e-23	**0,45**	1.02371907339988e-10
PPP2R5C	ENSG00000078304	**-0,46**	1.24003157098214e-11	**0,17**	0.0216340555537631
PPP2R5D	ENSG00000112640	**-0,59**	5.11323513896183e-19	**-0,05**	0.533061354004436
PPP2R5E	ENSG00000154001	**-0,63**	1.24765630115595e-22	**-0,07**	0.338795382647211
PPP5C	ENSG00000011485	**0,31**	1.12725309524602e-05	**0,15**	0.0410603305318604
PRKACA	ENSG00000072062	**-0,02**	0.811248454719882	**0,30**	1.80774391133837e-05
PRKCD	ENSG00000163932	**-0,35**	8.07477316843992e-07	**-0,18**	0.0106597716713838
PRLR	ENSG00000113494	**-0,35**	4.78539784286799e-07	**0,25**	0.000489188291246032
PSMA1	ENSG00000129084	**-0,63**	5.91093840175842e-23	**-0,22**	0.00243969049691303
PSMA2	ENSG00000106588	**-0,69**	6.97558930068525e-29	**-0,22**	0.00277387524746391
PSMA3	ENSG00000100567	**-0,57**	9.31546119173232e-18	**-0,26**	0.000359242634911862
PSMA4	ENSG00000041357	**-0,70**	1.59526637778301e-29	**-0,27**	0.0001997382402363
PSMA5	ENSG00000143106	**-0,62**	1.22283291242e-21	**-0,13**	0.0689881884176315
PSMA6	ENSG00000100902	**-0,68**	2.4341994735323e-27	**-0,28**	9.64207797588967e-05
PSMA7	ENSG00000101182	**0,09**	0.201609746145319	**-0,33**	4.46556345624218e-06
PSMB1	ENSG00000008018	**-0,40**	6.58571975830427e-09	**-0,14**	0.0543987120862557
PSMB10	ENSG00000205220	**0,57**	5.46250678486104e-18	**-0,20**	0.00467914779854022
PSMB2	ENSG00000126067	**-0,26**	0.000352502494165879	**-0,24**	0.000837427358250416
PSMB4	ENSG00000159377	**-0,52**	1.81392014848244e-14	**-0,10**	0.154514540724103
PSMB5	ENSG00000100804	**-0,53**	2.09068676857083e-15	**0,12**	0.100115973272358
PSMB6	ENSG00000142507	**-0,40**	1.55165249908528e-08	**-0,06**	0.403769947502936
PSMB7	ENSG00000136930	**-0,71**	1.01161102429398e-30	**-0,29**	4.75950229454801e-05
PSMB8	ENSG00000204264	**-0,61**	3.10896602614374e-21	**-0,37**	1.56551206915383e-07
PSMB9	ENSG00000240065	**-0,07**	0.31394140105893	**-0,33**	2.37041173053431e-06
PSMC1	ENSG00000100764	**-0,47**	4.48283161451359e-12	**-0,18**	0.0109068276529132
PSMC2	ENSG00000161057	**-0,64**	1.15967334501735e-23	**0,01**	0.839520912422848
PSMC3	ENSG00000165916	**-0,46**	3.41835255513657e-11	**-0,10**	0.155875761077245
PSMC4	ENSG00000013275	**-0,18**	0.0142375115378979	**-0,19**	0.00692976553882717
PSMC5	ENSG00000087191	**-0,56**	2.41330104610685e-17	**0,05**	0.529987909623038
PSMC6	ENSG00000100519	**-0,60**	4.59730298832783e-20	**-0,13**	0.0674482024632227
PSMD1	ENSG00000173692	**-0,41**	4.74070200732273e-09	**0,15**	0.0452669845205855
PSMD10	ENSG00000101843	**-0,61**	4.627573628671e-21	**0,12**	0.108996242986061
PSMD11	ENSG00000108671	**-0,53**	2.0118848243113e-15	**-0,12**	0.0866336820354735
PSMD12	ENSG00000197170	**-0,36**	2.83806967509658e-07	**-0,19**	0.00786496076479258
PSMD13	ENSG00000185627	**-0,69**	1.01542754970781e-28	**-0,25**	0.000540829682780779
PSMD14	ENSG00000115233	**-0,47**	3.93562400048325e-12	**-0,19**	0.00725805034528575
PSMD2	ENSG00000175166	**-0,63**	1.24551034008536e-22	**-0,04**	0.62956761858887
PSMD3	ENSG00000108344	**-0,05**	0.46781563891398	**-0,17**	0.0224742031982313
PSMD4	ENSG00000159352	**-0,62**	7.1381410022842e-22	**-0,12**	0.109714837383775
PSMD5	ENSG00000095261	**-0,48**	1.39963539759038e-12	**0,09**	0.201442479756507
PSMD6	ENSG00000163636	**-0,13**	0.0635132405744652	**-0,33**	4.2750865695564e-06
PSMD7	ENSG00000103035	**-0,30**	3.4107898361622e-05	**-0,19**	0.00788862672999768
PSMD8	ENSG00000099341	**-0,02**	0.836402488045771	**-0,10**	0.180945552323428
PSMD9	ENSG00000110801	**0,27**	0.000162609651579055	**-0,30**	2.12762668062426e-05
PSME1	ENSG00000092010	**-0,45**	1.05830651223265e-10	**-0,24**	0.000743550092461142
PSME2	ENSG00000100911	**-0,53**	1.78027227145649e-15	**-0,26**	0.000269999842557765
PSME3	ENSG00000131467	**-0,65**	6.44367280155985e-24	**-0,21**	0.004243908170461
PSME4	ENSG00000068878	**-0,69**	8.47686877068856e-28	**-0,31**	1.19988348855721e-05
PSMF1	ENSG00000125818	**-0,08**	0.27649658186066	**0,09**	0.211586853080366
PSPN	ENSG00000125650	**0,25**	0.000391748768345642	**0,34**	2.06101331582609e-06
PTAFR	ENSG00000169403	**-0,40**	7.80619939501601e-09	**-0,22**	0.00175393574728179
PTGS2	ENSG00000073756	**-0,37**	1.06202411715418e-07	**-0,41**	5.66990919222164e-09
PTK2	ENSG00000169398	**-0,42**	1.07152054821376e-09	**0,24**	0.000760328366267928

PTK2B	ENSG00000120899		**0,18**	0.0104280244768455		**-0,09**	0.227088637359813
PTPN1	ENSG00000196396		**0,13**	0.0757562099293001		**0,19**	0.00894536646261136
PTPN11	ENSG00000179295		**-0,44**	2.83742915818007e-10		**0,19**	0.00849151413351793
PTPN2	ENSG00000175354		**-0,40**	8.0158053878309e-09		**-0,56**	7.30794410228303e-17
PTPN6	ENSG00000111679		**0,28**	9.07159554868796e-05		**-0,09**	0.193182499885867
PTPRA	ENSG00000132670		**-0,19**	0.0100332626684455		**0,37**	1.03574261973419e-07
RAE1	ENSG00000101146		**-0,30**	3.34846462301726e-05		**-0,14**	0.0568252262484159
RAF1	ENSG00000132155		**-0,63**	2.07438219146617e-22		**-0,58**	2.05221822580788e-18
RAG1	ENSG00000166349		**-0,45**	6.08272380941514e-11		**-0,16**	0.0321046862483524
RAG2	ENSG00000175097		**0,00**	0.960816602179011		**-0,05**	0.476322243024174
RANBP2	ENSG00000153201		**-0,23**	0.0014755121451859		**0,26**	0.000242215222635279
RANBP9	ENSG00000010017		**-0,44**	3.08446592250804e-10		**0,06**	0.405437584768801
RAP1A	ENSG00000116473		**-0,62**	2.68365683637027e-21		**-0,06**	0.375865435998495
RAP1B	ENSG00000127314		**-0,48**	2.43262422550207e-12		**-0,28**	8.36087851979959e-05
RAPGEF1	ENSG00000107263		**0,47**	7.21420063705013e-12		**-0,15**	0.0328580551711442
RAPGEF2	ENSG00000109756		**-0,55**	8.62364043874343e-17		**-0,39**	3.48163787733981e-08
RASA1	ENSG00000145715		**-0,23**	0.00163724540007045		**0,21**	0.00422151354769547
RASA2	ENSG00000155903		**-0,44**	2.00136031350772e-10		**-0,57**	1.35022620482298e-17
RASA3	ENSG00000185989		**0,18**	0.0120536003079959		**-0,13**	0.0688132564373196
RASA4	ENSG00000105808		**0,69**	2.27225713975035e-28		**0,46**	3.38227661797595e-11
RASAL1	ENSG00000111344		**0,09**	0.197020922071119		**-0,18**	0.0139437752807615
RASAL2	ENSG00000075391		**-0,57**	3.4345593376472e-18		**-0,25**	0.000629779600307539
RASAL3	ENSG00000105122		**0,37**	2.0424474413675e-07		**-0,12**	0.0913776715973027
RASGEF1A	ENSG00000198915		**0,30**	2.08391972882806e-05		**0,63**	6.01509495709097e-23
RASGRF1	ENSG00000058335		**0,27**	0.00012526437903715		**0,74**	7.10905913655742e-35
RASGRF2	ENSG00000113319		**0,24**	0.000900942106677157		**0,35**	1.00263490484336e-06
RASGRP1	ENSG00000172575		**-0,12**	0.11219567877165		**0,44**	1.24364310748378e-10
RASGRP3	ENSG00000152689		**-0,43**	5.11724716822638e-10		**-0,60**	4.98639628508352e-20
RASGRP4	ENSG00000171777		**0,20**	0.00452824398849106		**-0,04**	0.588323087183766
RBX1	ENSG00000100387		**-0,37**	1.26598373615541e-07		**-0,11**	0.117758027066243
RELA	ENSG00000173039		**0,33**	2.60509100663762e-06		**-0,32**	4.81257582740091e-06
RELB	ENSG00000104856		**0,34**	1.49315930795871e-06		**-0,40**	1.15561076208071e-08
RET	ENSG00000165731		**0,31**	1.75515792857121e-05		**0,32**	6.74857844552179e-06
RHOU	ENSG00000116574		**-0,57**	8.72103181529119e-18		**-0,45**	5.63342696299737e-11
RIPK2	ENSG00000104312		**-0,40**	7.76508291623069e-09		**-0,59**	4.30641191933855e-19
RNASEL	ENSG00000135828		**-0,23**	0.00109628575311977		**0,14**	0.060581002434791
RORA	ENSG00000069667		**-0,01**	0.87186285420794		**0,01**	0.941001231698221
RORC	ENSG00000143365		**0,23**	0.00128873734992982		**0,23**	0.00161669938527305
RPS27A	ENSG00000143947		**-0,52**	1.29280938389443e-14		**-0,34**	1.10727580600848e-06
RSAD2	ENSG00000134321		**-0,22**	0.00234969317224505		**-0,19**	0.00789868450016011
S1PR1	ENSG00000170989		**0,02**	0.819116264631616		**0,10**	0.15711272946185
SAA1	ENSG00000173432		**-0,20**	0.00533019861105571		**-0,17**	0.0223168546849501
SAMHD1	ENSG00000101347		**-0,43**	4.29490781061603e-10		**-0,09**	0.239278167595729
SEH1L	ENSG00000085415		**-0,56**	4.93599592435943e-17		**-0,07**	0.330701210950543
SH2B1	ENSG00000178188		**0,65**	4.76530834506318e-24		**0,03**	0.661961164327496
SHC1	ENSG00000160691		**-0,15**	0.0334010352016602		**-0,28**	9.70767651076089e-05
SHC2	ENSG00000129946		**0,70**	4.10151320017954e-29		**-0,06**	0.425304112263489
SHC3	ENSG00000148082		**0,08**	0.24842868160891		**-0,12**	0.0996121122533175
SKP1	ENSG00000113558		**-0,62**	1.19710147539641e-21		**-0,09**	0.199328127159309
SMARCA4	ENSG00000127616		**0,53**	2.24356595606207e-15		**-0,01**	0.882042182699483
SOCS1	ENSG00000185338		**0,62**	1.13362876375065e-21		**-0,11**	0.135148843402378
SOCS2	ENSG00000120833		**0,17**	0.0169989833644122		**-0,07**	0.325388859958781
SOCS2-AS1	ENSG00000246985		**0,16**	0.0275537081020637		**0,16**	0.0257654666230072
SOCS3	ENSG00000184557		**0,24**	0.000917143429051332		**-0,39**	1.89577019821364e-08
SOCS4	ENSG00000180008		**-0,42**	1.09908953291492e-09		**0,15**	0.0337534242591804
SOCS5	ENSG00000171150		**-0,34**	1.16229795500811e-06		**0,28**	7.78587049118092e-05
SOCS5P4	ENSG00000227536		**0,15**	0.0449913041068375		**0,00**	0.953049055954659
SOCS6	ENSG00000170677		**-0,45**	5.81029926580992e-11		**0,24**	0.000833671270241291

SOS1	ENSG00000115904	**-0,51**	7.07731058980343e-14	**0,18**	0.013979692177311
SOX2	ENSG00000181449	**-0,07**	0.342908890948238	**-0,08**	0.2480840686946
SP100	ENSG00000067066	**-0,69**	5.24596293123841e-28	**-0,67**	1.06105331400465e-26
SPRED1	ENSG00000166068	**-0,68**	6.25382886727468e-27	**-0,31**	1.72177408979535e-05
SPRED2	ENSG00000198369	**-0,32**	6.26519554144452e-06	**-0,18**	0.0122258388314194
SPRED3	ENSG00000188766	**0,63**	1.02458257304999e-22	**0,19**	0.00858261006652422
SPTAN1	ENSG00000197694	**-0,29**	4.36784209963666e-05	**-0,15**	0.0323907834283405
SPTB	ENSG00000070182	**0,61**	1.56970670352961e-20	**0,59**	3.42176195722659e-19
SPTBN1	ENSG00000115306	**-0,48**	2.66300953936054e-12	**-0,39**	2.54246882391312e-08
SPTBN2	ENSG00000173898	**0,80**	6.43571263031852e-43	**0,23**	0.00123864311713928
SPTBN4	ENSG00000160460	**0,83**	6.93874620907857e-49	**0,33**	3.03342572919351e-06
SPTBN5	ENSG00000137877	**0,48**	1.58147972287098e-12	**-0,26**	0.000353404661915741
SQSTM1	ENSG00000161011	**0,34**	1.0468206756078e-06	**-0,26**	0.000240691537685364
SRC	ENSG00000197122	**0,08**	0.262402472097997	**-0,39**	2.39005445263932e-08
STAT1	ENSG00000115415	**-0,46**	1.4084182737644e-11	**0,08**	0.270253906811317
STAT2	ENSG00000170581	**-0,39**	1.74298679914003e-08	**0,06**	0.42665854407596
STAT3	ENSG00000168610	**-0,46**	1.24763123118793e-11	**0,13**	0.0793916043044484
STAT5A	ENSG00000126561	**-0,17**	0.0193706168094797	**-0,35**	8.63634668143367e-07
STAT5B	ENSG00000173757	**-0,08**	0.246635997702101	**0,18**	0.0130559703411971
STAT6	ENSG00000166888	**-0,25**	0.000617279201061092	**-0,34**	1.44452420385678e-06
STX1A	ENSG00000106089	**0,74**	9.80358947408866e-35	**0,33**	3.69356064054362e-06
STX3	ENSG00000166900	**-0,20**	0.0049334206878707	**-0,07**	0.315421789938787
STX4	ENSG00000103496	**-0,02**	0.752224523715741	**-0,37**	1.08476798350082e-07
SUMO1	ENSG00000116030	**-0,53**	2.47603772375236e-15	**-0,12**	0.107537020215407
SYK	ENSG00000165025	**-0,56**	2.27291333125735e-17	**-0,24**	0.000919588522465793
SYNGAP1	ENSG00000197283	**0,57**	1.45215668549315e-17	**0,33**	4.4782837044856e-06
TAB1	ENSG00000100324	**0,63**	1.28386119895019e-22	**0,03**	0.66563699546499
TAB2	ENSG00000055208	**-0,41**	5.05079019304517e-09	**0,04**	0.573441685779374
TAB3	ENSG00000157625	**-0,52**	1.75063160477548e-14	**0,01**	0.939505621197513
TEC	ENSG00000135605	**0,04**	0.620873521725902	**0,27**	0.000198382251602702
TEK	ENSG00000120156	**-0,22**	0.00272244213226528	**0,12**	0.0909038862850218
TGFB1	ENSG00000105329	**0,55**	1.32308433819893e-16	**0,01**	0.897066530135416
TIMP1	ENSG00000102265	**0,17**	0.0192965888765126	**0,06**	0.420418613804641
TLN1	ENSG00000137076	**0,07**	0.357222755155559	**0,08**	0.279649653625641
TNF	ENSG00000232810	**-0,17**	0.0219343961824148	**-0,33**	2.27682620675884e-06
TNFRSF11A	ENSG00000141655	**-0,05**	0.530375301551475	**0,18**	0.0148482737165256
TNFRSF11B	ENSG00000164761	**-0,33**	4.20310444357322e-06	**-0,10**	0.177258719078243
TNFRSF12A	ENSG00000006327	**0,44**	1.33906626529597e-10	**-0,32**	7.51562673086217e-06
TNFRSF14	ENSG00000157873	**0,67**	6.21552243012454e-26	**-0,07**	0.348979672551429
TNFRSF18	ENSG00000186891	**0,53**	2.66139092572026e-15	**-0,07**	0.343631083939922
TNFRSF1A	ENSG00000067182	**0,16**	0.0249198701319338	**-0,31**	1.15091426619657e-05
TNFRSF1B	ENSG00000028137	**-0,06**	0.404942955135092	**-0,14**	0.0477794458894424
TNFRSF25	ENSG00000215788	**0,43**	6.47770095024338e-10	**-0,11**	0.114125196411887
TNFRSF4	ENSG00000186827	**0,74**	2.16882986031124e-34	**0,04**	0.571010011360076
TNFRSF6B	ENSG00000243509	**0,62**	2.27464606718979e-21	**-0,04**	0.607501024867946
TNFRSF9	ENSG00000049249	**-0,50**	1.0070028517877e-13	**-0,35**	8.87565160868024e-07
TNFSF11	ENSG00000120659	**-0,38**	5.60512313684955e-08	**-0,35**	7.11257109015937e-07
TNFSF12	ENSG00000239697	**0,47**	6.09118885732618e-12	**0,05**	0.534358536366696
TNFSF13	ENSG00000161955	**-0,12**	0.108513720967559	**-0,11**	0.124282656441977
TNFSF13B	ENSG00000102524	**0,03**	0.671849702058623	**-0,15**	0.0449051433756027
TNFSF14	ENSG00000125735	**-0,13**	0.067541145631017	**-0,28**	6.87163173038349e-05
TNFSF15	ENSG00000181634	**-0,43**	5.7751199031524e-10	**-0,24**	0.000762369856125732
TNFSF18	ENSG00000120337	**-0,22**	0.00256556137585864	**-0,05**	0.529961608286036
TNFSF4	ENSG00000117586	**-0,05**	0.513552135998122	**0,52**	2.33517396890738e-14
TNFSF9	ENSG00000125657	**0,16**	0.0313243857917947	**-0,36**	4.55003719792772e-07
TNIP2	ENSG00000168884	**0,55**	2.95516914995812e-16	**-0,21**	0.00359823075359182
TOLLIP	ENSG00000078902	**0,83**	5.81664600081825e-50	**0,27**	0.000184423083762339
TP53	ENSG00000141510	**-0,33**	2.50339530430448e-06	**-0,13**	0.0795110617038005

TPR	ENSG00000047410	**-0,28**	9.1218883555313e-05	**-0,03**	0.670969248921206
TRAF2	ENSG00000127191	**0,62**	1.04382589432806e-21	**-0,03**	0.656224728116167
TRAF3	ENSG00000131323	**0,56**	1.74566636174895e-17	**0,09**	0.213743615770432
TRAF6	ENSG00000175104	**-0,55**	8.75714446453464e-17	**-0,03**	0.708375135881821
TRIM10	ENSG00000204613	**-0,23**	0.00156289312372338	**-0,24**	0.000974832087318299
TRIM14	ENSG00000106785	**0,20**	0.00517951985555743	**-0,03**	0.700124652637551
TRIM17	ENSG00000162931	**0,28**	7.0171863253822e-05	**-0,15**	0.0400242635409909
TRIM2	ENSG00000109654	**-0,06**	0.404391480392056	**0,53**	3.48048683374772e-15
TRIM21	ENSG00000132109	**-0,52**	1.69581064085608e-14	**-0,23**	0.00114695604613319
TRIM22	ENSG00000132274	**-0,29**	5.60814749934302e-05	**-0,20**	0.00657301357749269
TRIM25	ENSG00000121060	**-0,52**	6.71996864034073e-15	**-0,21**	0.00382735405395848
TRIM26	ENSG00000234127	**-0,17**	0.0188485001001899	**-0,29**	4.20187009111721e-05
TRIM29	ENSG00000137699	**-0,11**	0.136420081544235	**-0,19**	0.00917730597847544
TRIM3	ENSG00000110171	**0,65**	2.28360443101778e-24	**0,18**	0.0153836516626748
TRIM31	ENSG00000204616	**-0,15**	0.0351680587470194	**-0,25**	0.000410941117778941
TRIM34	ENSG00000258659	**-0,47**	4.18960538952854e-12	**-0,21**	0.00307383823744581
TRIM35	ENSG00000104228	**-0,01**	0.934064622295998	**0,12**	0.101272344512958
TRIM38	ENSG00000112343	**-0,51**	3.94248494347381e-14	**-0,31**	1.5450032546911e-05
TRIM45	ENSG00000134253	**-0,28**	7.80981009979289e-05	**-0,36**	3.74850625143589e-07
TRIM46	ENSG00000163462	**0,71**	2.6361543793505e-30	**0,18**	0.010887443150471
TRIM5	ENSG00000132256	**-0,49**	3.73190027201171e-13	**-0,27**	0.000168554525425517
TRIM6	ENSG00000121236	**-0,56**	4.72032373754496e-17	**-0,39**	2.67008418478172e-08
TRIM62	ENSG00000116525	**0,11**	0.139952373286212	**0,02**	0.798763084780243
TRIM68	ENSG00000167333	**-0,50**	2.53573098673162e-13	**0,16**	0.0252675494280328
TRIM8	ENSG00000171206	**0,54**	3.93309820324546e-16	**-0,15**	0.0422667660760826
TWIST1	ENSG00000122691	**0,35**	8.12817948872299e-07	**-0,09**	0.204810417583056
TXLNA	ENSG00000084652	**-0,12**	0.0884062756279006	**0,42**	1.31225027256287e-09
TYK2	ENSG00000105397	**0,65**	1.55184202437123e-24	**-0,02**	0.769230270167196
UBA3	ENSG00000144744	**-0,59**	4.32557498833879e-19	**-0,08**	0.283724776655148
UBA52	ENSG00000221983	**-0,72**	1.86618598734546e-31	**-0,24**	0.000873939378966847
UBA7	ENSG00000182179	**0,05**	0.480049135193552	**-0,23**	0.00158606487991596
UBB	ENSG00000170315	**-0,59**	2.50119210933739e-19	**0,14**	0.0625638107445379
UBC	ENSG00000150991	**-0,56**	2.78723123981906e-17	**-0,07**	0.364084254050423
UBE2E1	ENSG00000170142	**-0,72**	2.09272003809478e-31	**-0,20**	0.00612306999863171
UBE2L6	ENSG00000156587	**-0,40**	1.07744726518065e-08	**-0,01**	0.928487466220116
UBE2M	ENSG00000130725	**0,58**	2.79356741447247e-18	**0,02**	0.820551005180682
UBE2N	ENSG00000177889	**-0,67**	2.73300126693736e-26	**-0,03**	0.639894938211311
UBE2V1	ENSG00000244687	**-0,76**	4.52754229365559e-37	**-0,16**	0.0243507130100157
USP18	ENSG00000184979	**-0,22**	0.00230514533456041	**0,38**	4.49955508301541e-08
USP41	ENSG00000161133	**-0,09**	0.235345585994485	**0,53**	5.97833039674411e-15
VAV1	ENSG00000141968	**-0,22**	0.00188089971434898	**-0,11**	0.137469707678826
VCAM1	ENSG00000162692	**-0,49**	9.16998464331095e-13	**-0,39**	1.73018586390805e-08
VCL	ENSG00000035403	**-0,46**	1.73507451509654e-11	**-0,13**	0.0666242946820397
WDR83	ENSG00000123154	**0,50**	3.35451945973665e-13	**-0,07**	0.37026468253741
VEGFA	ENSG00000112715	**0,53**	1.81895745605374e-15	**0,23**	0.00121702159828756
VIM	ENSG00000026025	**-0,17**	0.0197071921753891	**-0,22**	0.0025610374159709
VWF	ENSG00000110799	**0,13**	0.0668068058761352	**0,06**	0.441535516380717
XAF1	ENSG00000132530	**-0,12**	0.10030354173073	**-0,04**	0.607941869840259
YES1	ENSG00000176105	**-0,51**	5.02818472381152e-14	**-0,36**	2.85572502308216e-07
YWHAB	ENSG00000166913	**-0,58**	1.11996181937778e-18	**-0,10**	0.18611295501768
YWHAZ	ENSG00000164924	**-0,68**	7.43283883774398e-27	**-0,43**	6.05930707585603e-10
ZEB1	ENSG00000148516	**-0,32**	8.1544148872618e-06	**0,09**	0.235240783189018

Table S2C

***MAFA* and *MAFB* co-expression correlations with gene-induced downstream cytokine signaling**

Genename	GeneID	MAFA_GC	*P*-value	MAFB_GC	*P*-value
ACE	ENSG00000159640	**0,36**	3.07221154809881e-07	**0,24**	0.00076864978212352
ADCY1	ENSG00000164742	**0,34**	1.23002449200616e-06	**0,70**	4.00921961866783e-29
AGER	ENSG00000204305	**0,39**	3.59588223542614e-08	**-0,17**	0.0174285717422343
AGTR1	ENSG00000144891	**-0,07**	0.31248549648881	**0,12**	0.10662154160853
AKT1	ENSG00000142208	**0,49**	4.07315761278472e-13	**-0,12**	0.0868735039250571
AKT2	ENSG00000105221	**0,49**	3.72634275580924e-13	**-0,19**	0.00818748202932539
AKT3	ENSG00000117020	**0,15**	0.0403729970246283	**0,58**	1.18585366248312e-18
ALOX5	ENSG00000012779	**0,30**	1.87152802173304e-05	**-0,06**	0.437355233947124
ANTXR1	ENSG00000169604	**-0,33**	3.36899260340411e-06	**0,08**	0.243951849682682
ANTXR2	ENSG00000163297	**-0,50**	3.09017986607524e-13	**-0,25**	0.000398844463605281
AOX1	ENSG00000138356	**-0,34**	2.14361228521916e-06	**-0,07**	0.327134306896325
APEX1	ENSG00000100823	**-0,73**	4.99792910510267e-33	**-0,24**	0.000823482510460096
APP	ENSG00000142192	**-0,47**	3.89752983276076e-12	**0,16**	0.0240831635670573
ARIH2	ENSG00000177479	**-0,44**	1.56498146449489e-10	**-0,14**	0.0523466306426547
ATF1	ENSG00000123268	**-0,56**	4.24181941097132e-17	**0,02**	0.804377996583631
ATF2	ENSG00000115966	**-0,30**	3.2891616577512e-05	**0,32**	8.62644875169852e-06
ATG12	ENSG00000145782	**-0,64**	1.69389230749401e-23	**-0,20**	0.00675881239009479
ATG16L1	ENSG00000085978	**-0,40**	1.22327485014815e-08	**-0,17**	0.0212374436233437
ATG5	ENSG00000057663	**-0,47**	4.90127566011895e-12	**-0,31**	1.11914538411058e-05
ATRAID	ENSG00000138085	**0,07**	0.353462838460288	**0,14**	0.0486641035106652
B2M	ENSG00000166710	**-0,63**	1.57472746308634e-22	**-0,26**	0.000301092518759588
BACH2	ENSG00000112182	**-0,29**	4.57520038667183e-05	**-0,41**	5.2898841627837e-09
BATF	ENSG00000156127	**0,15**	0.040132158107048	**-0,13**	0.0763698625994746
BCL10	ENSG00000142867	**-0,59**	2.77171789458138e-19	**-0,61**	3.75492806887587e-21
BCL11B	ENSG00000127152	**-0,22**	0.00179908667005796	**-0,11**	0.119566751490548
BCL2	ENSG00000171791	**-0,61**	1.57569459075146e-20	**-0,37**	1.25077746579985e-07
BCL2L1	ENSG00000171552	**-0,05**	0.485231362791119	**-0,20**	0.00575913089355628
BCL6	ENSG00000113916	**-0,13**	0.0713773538285527	**-0,51**	6.06666734707207e-14
BDKRB2	ENSG00000168398	**-0,46**	3.05039888861174e-11	**-0,23**	0.00130689270892648
BIRC2	ENSG00000110330	**-0,70**	1.91247074936007e-29	**-0,49**	3.47471121485483e-13
BIRC3	ENSG00000023445	**-0,53**	5.64894897445921e-15	**-0,52**	1.01889991519376e-14
BLNK	ENSG00000095585	**-0,43**	7.83727840585589e-10	**-0,42**	2.33253796601925e-09
BMS1P4	ENSG00000271816	**0,12**	0.0961128686830574	**-0,25**	0.000459505853007303
BMS1P4	ENSG00000242338	**0,17**	0.0213790518064362	**-0,22**	0.00237511311369295
BMX	ENSG00000102010	**-0,13**	0.0824375599515399	**-0,12**	0.0862478163888359
BRCC3	ENSG00000185515	**-0,60**	4.76915223850473e-20	**0,00**	0.948826447152677
BTK	ENSG00000010671	**-0,25**	0.000460965731176732	**0,09**	0.239600973233454
BTRC	ENSG00000166167	**-0,41**	2.45485533120055e-09	**0,32**	6.28551551572272e-06
C1QA	ENSG00000173372	**0,17**	0.0184792411047264	**0,12**	0.103291903030109
C1QB	ENSG00000173369	**0,13**	0.0662561426266017	**0,15**	0.0379178898234398
C1QBP	ENSG00000108561	**-0,27**	0.000179898229939293	**-0,25**	0.000580065877950579
C1QC	ENSG00000159189	**0,35**	8.93754611036145e-07	**0,11**	0.135483924559332
C3	ENSG00000125730	**-0,31**	9.18296977902629e-06	**-0,34**	1.12574702169532e-06
CABIN1	ENSG00000099991	**0,31**	1.19236825777335e-05	**0,32**	4.63071555219305e-06
CALR	ENSG00000179218	**-0,54**	5.86251283144638e-16	**-0,01**	0.908583859637705
CARD11	ENSG00000198286	**0,31**	1.08474132109785e-05	**0,04**	0.553737198478194
CARD16	ENSG00000204397	**-0,10**	0.185296309718169	**-0,11**	0.146466963695937
CARD6	ENSG00000132357	**-0,66**	4.67365799818045e-25	**-0,47**	4.11341831900753e-12
CARD8	ENSG00000105483	**-0,17**	0.021431909686224	**0,03**	0.637331568977448
CARD9	ENSG00000187796	**0,68**	5.17865902357258e-27	**-0,01**	0.873620011090735
CASP1	ENSG00000137752	**-0,37**	1.27731924197375e-07	**-0,28**	9.59804207795709e-05
CASP12	ENSG00000204403	**-0,10**	0.159292141779571	**0,03**	0.664342030620829
CASP4	ENSG00000196954	**-0,64**	2.77024102061509e-23	**-0,66**	2.75533330707951e-25

CASP8	ENSG00000064012		**-0,50**	2.00944932332427e-13		**-0,40**	8.97088158582183e-09
CASR	ENSG00000036828		**0,10**	0.157107715298627		**0,67**	2.73499975540068e-26
CBL	ENSG00000110395		**-0,55**	3.46855474788441e-16		**0,05**	0.504839833517787
CBLB	ENSG00000114423		**-0,43**	7.85886612417744e-10		**-0,03**	0.726133961907254
CCL2	ENSG00000108691		**-0,47**	6.41416189475132e-12		**-0,46**	1.41332859075084e-11
CCND1	ENSG00000110092		**-0,33**	4.00428196513867e-06		**-0,37**	1.33681641242413e-07
CCND2	ENSG00000118971		**-0,25**	0.000390220294231601		**0,18**	0.01226290018092
CCND3	ENSG00000112576		**0,63**	1.99608430889085e-22		**0,43**	6.19337045886328e-10
CD14	ENSG00000170458		**0,63**	1.80968303395632e-22		**0,10**	0.15297374967771
CD180	ENSG00000134061		**-0,30**	3.11125104536506e-05		**0,09**	0.221293104019655
CD2	ENSG00000116824		**-0,31**	1.31400834625803e-05		**-0,21**	0.00393827816533609
CD247	ENSG00000198821		**-0,05**	0.453918883964064		**-0,23**	0.00169976049177098
CD274	ENSG00000120217		**-0,41**	3.48301356939234e-09		**-0,21**	0.00309209704619428
CD276	ENSG00000103855		**0,29**	3.60074165967333e-05		**-0,12**	0.0861441389517456
CD2AP	ENSG00000198087		**-0,52**	9.33056482053055e-15		**-0,24**	0.000722228872781915
CD34	ENSG00000174059		**-0,03**	0.691145710777611		**0,09**	0.238991357785317
CD36	ENSG00000135218		**-0,14**	0.0501483907875544		**0,33**	3.98276990022087e-06
CD38	ENSG00000004468		**-0,25**	0.000451467300035776		**-0,10**	0.183691593006662
CD3E	ENSG00000198851		**-0,33**	2.23054669030193e-06		**-0,25**	0.000519246889560994
CD40	ENSG00000101017		**-0,22**	0.00245987679052234		**-0,27**	0.000173068923433882
CD44	ENSG00000026508		**-0,61**	3.86976825536918e-21		**-0,55**	8.21955012712848e-17
CD46	ENSG00000117335		**-0,38**	4.5217780783939e-08		**0,14**	0.0468538562862642
CD63	ENSG00000135404		**-0,47**	4.62592637766424e-12		**-0,21**	0.00312753635346265
CD74	ENSG00000019582		**-0,22**	0.00176761821586089		**-0,43**	7.37162803041539e-10
CD79A	ENSG00000105369		**0,09**	0.195021460756633		**-0,21**	0.00393681661902317
CD80	ENSG00000121594		**-0,09**	0.222628048127229		**-0,26**	0.000315313495242874
CD82	ENSG00000085117		**0,52**	6.86972257855284e-15		**0,23**	0.00158324328647096
CD83	ENSG00000112149		**-0,31**	1.57624387518375e-05		**-0,10**	0.155603315294833
CD86	ENSG00000114013		**-0,45**	5.19997845189528e-11		**-0,22**	0.00274785207855291
CD8A	ENSG00000153563		**-0,14**	0.0511538289510875		**0,12**	0.0872547964958877
CDCP1	ENSG00000163814		**-0,52**	2.22049758653104e-14		**-0,13**	0.0785770499816104
CDIP1	ENSG00000089486		**0,43**	5.9545586917864e-10		**0,68**	1.99111413476404e-27
CDKN1A	ENSG00000124762		**0,21**	0.00313834927732949		**-0,13**	0.0807422336973029
CEACAM1	ENSG00000079385		**-0,33**	2.7445863111038e-06		**-0,05**	0.468684559310743
CEACAM5	ENSG00000105388		**-0,20**	0.00598873307632171		**-0,10**	0.174119666036429
CEBPA	ENSG00000245848		**0,21**	0.00304521634877005		**-0,34**	1.33299119629224e-06
CEBPB	ENSG00000172216		**0,60**	2.9899164695653e-20		**-0,17**	0.0189782157002947
CEBPD	ENSG00000221869		**0,55**	1.46597502611609e-16		**-0,24**	0.000846135608802876
CFLAR	ENSG00000003402		**-0,55**	8.85426705917221e-17		**-0,61**	4.70444178155653e-21
CHUK	ENSG00000213341		**-0,34**	1.1178268897534e-06		**0,20**	0.00625863331816267
CIITA	ENSG00000179583		**0,16**	0.0251925400006313		**0,05**	0.454992654836226
CISH	ENSG00000114737		**-0,04**	0.623436251380992		**-0,53**	1.83498073104994e-15
CNPY3	ENSG00000137161		**0,26**	0.000232340144421899		**0,18**	0.0137918276095558
CNTF	ENSG00000242689		**-0,38**	8.88862438427588e-08		**-0,15**	0.035450938097936
CNTFR	ENSG00000122756		**0,37**	2.04816568631161e-07		**-0,06**	0.411403060567298
COG8	ENSG00000272617		**0,33**	2.54106421135974e-06		**0,24**	0.000714724698464077
COG8	ENSG00000213380		**0,16**	0.0261138224580358		**0,04**	0.620638377021973
CREB1	ENSG00000118260		**-0,50**	2.03336285650518e-13		**0,13**	0.0669786486565456
CREBBP	ENSG00000005339		**0,21**	0.00401466401809987		**-0,14**	0.0520105793553384
CRHR1	ENSG00000120088		**-0,26**	0.00022533086282746		**0,37**	1.0127341939085e-07
CSF2	ENSG00000164400		**0,03**	0.681839701485133		**-0,33**	4.43750237595565e-06
CSF2RB	ENSG00000100368		**0,13**	0.0688725900856352		**0,05**	0.470530770022826
CSF3	ENSG00000108342		**0,18**	0.0154185750024194		**-0,36**	2.92319384299176e-07
CSF3R	ENSG00000119535		**-0,02**	0.827356498428349		**-0,05**	0.48260413478207
CTF1	ENSG00000150281		**0,06**	0.374255243047533		**-0,15**	0.0449285995603537
CTNNAL1	ENSG00000119326		**-0,55**	2.91947244597432e-16		**-0,47**	1.16545157428446e-11
CTSB	ENSG00000164733		**-0,23**	0.00164734200909167		**0,15**	0.0374069104674115
CTSK	ENSG00000143387		**-0,27**	0.000166098540894403		**-0,14**	0.0541527448122166

CTSL	ENSG00000135047		**-0,45**	6.64945503807615e-11		**-0,05**	0.481623419548414
CTSS	ENSG00000163131		**-0,64**	2.80608287840347e-23		**-0,44**	1.50630985858255e-10
CTSV	ENSG00000136943		**-0,07**	0.312328687620984		**0,25**	0.000546119597743917
CUEDC2	ENSG00000107874		**0,38**	8.52365558581324e-08		**0,11**	0.13155172264729
CUL1	ENSG00000055130		**-0,52**	2.14761458340907e-14		**-0,21**	0.00400553337582581
CXCL1	ENSG00000163739		**-0,21**	0.00367753408294341		**-0,41**	2.5377874669153e-09
CXCL10	ENSG00000169245		**-0,20**	0.00635049523593809		**-0,30**	1.90252887758536e-05
CXCL11	ENSG00000169248		**-0,25**	0.000407303894057176		**-0,23**	0.00109819780946129
CXCL12	ENSG00000107562		**-0,25**	0.000383990851726459		**-0,39**	2.10479387532965e-08
CXCL2	ENSG00000081041		**-0,17**	0.0193860342280525		**-0,47**	7.45811154682921e-12
CXCL3	ENSG00000163734		**-0,13**	0.0751678973976666		**-0,53**	3.42505660121794e-15
CXCL8	ENSG00000169429		**-0,42**	1.12097774486885e-09		**-0,54**	1.55144253234339e-15
CXCL9	ENSG00000138755		**-0,21**	0.00288462294587905		**-0,21**	0.00355173584180992
CXCR4	ENSG00000121966		**-0,18**	0.0125262267219206		**0,03**	0.650330003763597
CXCR5	ENSG00000160683		**0,34**	1.14022885829554e-06		**-0,30**	2.11468012073142e-05
CYB561D2	ENSG00000114395		**0,46**	1.48845693188467e-11		**-0,03**	0.664963989251457
CYBA	ENSG00000051523		**0,52**	1.63351244427542e-14		**-0,26**	0.000286402080127753
CYBB	ENSG00000165168		**-0,44**	2.64750126097317e-10		**-0,10**	0.162358053660834
CYLD	ENSG00000083799		**-0,24**	0.000867446080372755		**0,22**	0.00257779403575003
DAPP1	ENSG00000070190		**-0,37**	1.15785472813085e-07		**-0,32**	7.22587891589799e-06
DCLRE1C	ENSG00000152457		**-0,64**	2.94752586147688e-23		**-0,46**	2.15893958584831e-11
DDX58	ENSG00000107201		**-0,42**	9.47228472906936e-10		**-0,23**	0.00110291790660329
DHX33	ENSG00000005100		**-0,62**	1.84303262785406e-21		**-0,31**	1.25445246833463e-05
DHX36	ENSG00000174953		**-0,45**	1.01826907853331e-10		**0,20**	0.00500524683407353
DHX58	ENSG00000108771		**0,47**	4.42466552537902e-12		**-0,22**	0.00206682956848339
DHX9	ENSG00000135829		**-0,51**	4.06940241394339e-14		**-0,12**	0.11122258089935
DNM1	ENSG00000106976		**0,60**	2.27903640224175e-20		**0,50**	1.24954258666217e-13
DNM1L	ENSG00000087470		**-0,52**	1.65116124976206e-14		**0,15**	0.0388038796102578
DNM2	ENSG00000079805		**0,48**	1.87895913695288e-12		**-0,09**	0.203129019485856
DNM3	ENSG00000197959		**0,39**	2.202097629183e-08		**0,40**	1.03591433385072e-08
DUSP3	ENSG00000108861		**-0,37**	1.68260584981176e-07		**0,21**	0.00419548757052364
DUSP4	ENSG00000120875		**-0,42**	2.32293611490365e-09		**-0,54**	1.09789203695076e-15
DUSP6	ENSG00000139318		**-0,17**	0.0206498672669411		**-0,29**	5.71289633798277e-05
DUSP7	ENSG00000164086		**0,57**	4.05257135265869e-18		**0,03**	0.716312135057821
ECSIT	ENSG00000130159		**0,50**	1.06435295847241e-13		**-0,16**	0.0268018127541946
EEA1	ENSG00000102189		**-0,52**	6.91807588875939e-15		**0,03**	0.698535858465582
EGF	ENSG00000138798		**-0,41**	5.7824858431494e-09		**-0,32**	8.98574152974385e-06
EGFR	ENSG00000146648		**-0,60**	4.3058782674096e-20		**-0,50**	1.1243564967149e-13
EGR1	ENSG00000120738		**-0,14**	0.0623343681152722		**-0,22**	0.00280085718660578
ELK1	ENSG00000126767		**0,27**	0.000160530947133916		**0,00**	0.970772676472226
ENPP3	ENSG00000154269		**0,03**	0.722571319429362		**0,12**	0.0870954154617166
EP300	ENSG00000100393		**-0,21**	0.00340861626434952		**0,00**	0.990585855749299
EPOR	ENSG00000187266		**0,68**	1.0512794301319e-27		**0,06**	0.449496651539812
ERBIN	ENSG00000112851		**-0,68**	1.84040505573995e-27		**-0,30**	3.08744217174619e-05
ERC1	ENSG00000082805		**-0,37**	1.30976557715621e-07		**0,23**	0.00148115475931647
FADD	ENSG00000168040		**-0,15**	0.0356583211386548		**-0,22**	0.00279319763901373
FAS	ENSG00000026103		**-0,50**	2.27089514974497e-13		**-0,36**	4.30367878444592e-07
FBXW11	ENSG00000072803		**-0,34**	1.52499677719491e-06		**0,30**	2.63687140639665e-05
FBXW5	ENSG00000159069		**0,67**	8.300357758917e-26		**-0,07**	0.34772404092846
FER	ENSG00000151422		**-0,41**	6.1414569469831e-09		**0,11**	0.118017837300769
FGA	ENSG00000171560		**-0,31**	1.56959891589665e-05		**-0,31**	1.34666094371246e-05
FGB	ENSG00000171564		**-0,25**	0.000378372380822799		**-0,19**	0.0080915117011165
FGG	ENSG00000171557		**-0,29**	3.76327983815819e-05		**-0,32**	8.44356327212361e-06
FHL1	ENSG00000022267		**-0,03**	0.705289749761935		**0,46**	1.28645214407851e-11
FLI1	ENSG00000151702		**-0,08**	0.288590233390852		**0,12**	0.109616914294162
FOS	ENSG00000170345		**0,07**	0.319430866802173		**-0,10**	0.171969344062599
FOSB	ENSG00000125740		**-0,07**	0.346250438022188		**-0,19**	0.00692814571659591
FOSL1	ENSG00000175592		**0,01**	0.914382309139964		**-0,33**	2.55881682999694e-06

FOSL2	ENSG00000075426		**-0,35**	8.4636821023894e-07		**-0,64**	2.72553634975147e-23
FOXP3	ENSG00000049768		**0,47**	6.03958835098345e-12		**0,13**	0.0689972235202294
GABARAP	ENSG00000170296		**-0,48**	3.67438012266113e-12		**0,18**	0.0132480655612883
GABARAPL1	ENSG00000139112		**-0,41**	3.03874483722e-09		**-0,24**	0.000715774843026657
GABARAPL2	ENSG00000034713		**0,19**	0.00722966796322994		**0,22**	0.00201095226776799
GATA2	ENSG00000179348		**0,38**	4.45201041396795e-08		**-0,23**	0.00165022138823529
GATA3	ENSG00000107485		**0,07**	0.316648697971256		**-0,19**	0.0069102554727463
GATA6	ENSG00000141448		**0,18**	0.0118513556956707		**-0,36**	2.81561672589483e-07
GBP1	ENSG00000117228		**-0,54**	3.72749092694847e-16		**-0,39**	1.62642204239511e-08
GBP2	ENSG00000162645		**-0,48**	2.60404210434064e-12		**-0,48**	2.35061797063963e-12
GBP3	ENSG00000117226		**-0,33**	3.50073784638969e-06		**-0,26**	0.000333079671380302
GBP4	ENSG00000162654		**-0,20**	0.00599815698168757		**-0,01**	0.87584477265925
GBP5	ENSG00000154451		**-0,32**	8.76934311592565e-06		**-0,24**	0.000648027424968863
GDF15	ENSG00000130513		**0,53**	2.65575445929727e-15		**-0,20**	0.00653922299482418
GFAP	ENSG00000131095		**0,02**	0.829704642602318		**0,11**	0.138342656781757
GHR	ENSG00000112964		**-0,32**	8.68237515685064e-06		**0,29**	6.13402030775807e-05
GIMAP5	ENSG00000196329		**-0,17**	0.0183333113261417		**0,17**	0.017130288663123
GNA11	ENSG00000088256		**0,75**	4.42510410324174e-35		**0,11**	0.114958752856712
GNA14	ENSG00000156049		**-0,24**	0.000846535758117121		**-0,17**	0.0163801613613496
GNA15	ENSG00000060558		**0,24**	0.000664080634858414		**-0,15**	0.0453458975114817
GNAI1	ENSG00000127955		**0,07**	0.360975106197036		**0,65**	2.50539940076264e-24
GNAI2	ENSG00000114353		**0,72**	2.84968091546757e-31		**0,49**	7.51529355457076e-13
GNAI3	ENSG00000065135		**-0,68**	7.22903848301321e-27		**-0,30**	3.22859217667975e-05
GNAL	ENSG00000141404		**0,08**	0.271026598480733		**-0,03**	0.641203767508172
GNAO1	ENSG00000087258		**0,61**	1.27707576261866e-20		**0,75**	3.16438474852285e-36
GNAQ	ENSG00000156052		**-0,34**	1.12776930638562e-06		**0,25**	0.000436008994415049
GNAS	ENSG00000087460		**0,06**	0.396315918150228		**0,61**	1.8705399746648e-20
GPX1	ENSG00000233276		**0,64**	3.16946788665237e-23		**-0,12**	0.112601732785659
GRB2	ENSG00000177885		**-0,33**	4.11133454298968e-06		**0,00**	0.988232456850839
GRK6	ENSG00000198055		**0,60**	2.27519295088087e-20		**-0,09**	0.196655260820615
GSDMD	ENSG00000104518		**0,60**	2.14960484813796e-20		**-0,18**	0.0149668836944798
GZMB	ENSG00000100453		**-0,07**	0.351670093339684		**-0,15**	0.0340925504782046
HAVCR1	ENSG00000113249		**-0,35**	6.57283091896305e-07		**-0,13**	0.0687601242119925
HAVCR2	ENSG00000135077		**-0,30**	3.37417972950292e-05		**-0,12**	0.096150386924099
HCK	ENSG00000101336		**-0,24**	0.000667377904420875		**-0,03**	0.634449615819388
HCLS1	ENSG00000180353		**-0,25**	0.000470464901844308		**0,03**	0.65923540517845
HMGB1	ENSG00000189403		**-0,47**	1.09830958412856e-11		**-0,19**	0.00703428082896763
HMOX1	ENSG00000100292		**0,09**	0.201600022912768		**-0,07**	0.326888502930445
HRAS	ENSG00000174775		**0,55**	2.46001885119236e-16		**0,06**	0.405352115542166
HSP90AA1	ENSG00000080824		**-0,39**	3.46285886000608e-08		**0,10**	0.167349914358279
HSP90AB1	ENSG00000096384		**-0,60**	5.75613230831096e-20		**0,05**	0.480447459721814
HSP90B1	ENSG00000166598		**-0,30**	2.46119325311968e-05		**0,00**	0.96229049370025
ICAM2	ENSG00000108622		**0,11**	0.134328040339662		**0,32**	6.1274373747144e-06
ID3	ENSG00000117318		**0,59**	1.15986638978871e-19		**-0,08**	0.259396705013588
IDO1	ENSG00000131203		**-0,21**	0.00398221657944314		**-0,17**	0.0182573367158244
IFI16	ENSG00000163565		**-0,34**	1.67670760690685e-06		**-0,27**	0.000192580545122838
IFIH1	ENSG00000115267		**-0,35**	4.95295621421065e-07		**0,19**	0.0080246166595001
IFNAR1	ENSG00000142166		**-0,63**	1.93927974544317e-22		**-0,26**	0.000304375990188567
IFNAR2	ENSG00000159110		**-0,66**	1.12799580148387e-25		**-0,42**	1.9875301607214e-09
IFNGR1	ENSG00000027697		**-0,61**	9.20222557434055e-21		**-0,47**	6.89646309761597e-12
IFNGR2	ENSG00000159128		**-0,51**	2.75065468913454e-14		**-0,54**	4.3989143154181e-16
IFNLR1	ENSG00000185436		**-0,31**	1.556330238438e-05		**-0,13**	0.0754043913196151
IKBKB	ENSG00000104365		**0,00**	0.955333101431861		**-0,29**	6.32388919004456e-05
IKZF1	ENSG00000185811		**-0,32**	5.98788332420838e-06		**-0,15**	0.0327310047413403
IL10	ENSG00000136634		**-0,35**	6.62961598191475e-07		**-0,14**	0.0481751827565998
IL10RA	ENSG00000110324		**0,18**	0.0125206546242096		**0,57**	3.4786531533015e-18
IL10RB	ENSG00000243646		**-0,42**	9.56550701097362e-10		**-0,38**	5.57365766134934e-08
IL11	ENSG00000095752		**-0,07**	0.365283087770948		**-0,32**	7.52132567074106e-06

IL11RA	ENSG00000137070		**0,39**	1.96444396848656e-08		**-0,07**	0.319166387616187
IL12A	ENSG00000168811		**0,00**	0.991110726408829		**-0,38**	7.9570963243035e-08
IL12RB1	ENSG00000096996		**0,23**	0.00122230356369201		**0,15**	0.0390269569523579
IL12RB2	ENSG00000081985		**-0,33**	2.71023299394142e-06		**-0,30**	1.96597294538937e-05
IL13RA1	ENSG00000131724		**-0,53**	3.74050990258939e-15		**-0,02**	0.760937217074865
IL13RA2	ENSG00000123496		**-0,31**	1.25502366462693e-05		**-0,18**	0.0109951027134327
IL15	ENSG00000164136		**-0,22**	0.00267988795387774		**-0,70**	6.9321397775139e-29
IL15RA	ENSG00000134470		**0,02**	0.776052277699761		**-0,58**	2.89052913197342e-18
IL17D	ENSG00000172458		**0,35**	6.02171551746405e-07		**0,30**	1.98213111798617e-05
IL18	ENSG00000150782		**-0,27**	0.000193778534666499		**-0,32**	4.62993766015631e-06
IL1B	ENSG00000125538		**-0,35**	7.39860946753494e-07		**-0,35**	8.80488246544108e-07
IL1RN	ENSG00000136689		**-0,28**	7.23573823279185e-05		**-0,34**	1.91738457528817e-06
IL20RA	ENSG00000016402		**0,09**	0.193573144428191		**0,41**	3.82020705217257e-09
IL20RB	ENSG00000174564		**0,01**	0.867333638448443		**-0,22**	0.0023392089228126
IL21R	ENSG00000103522		**0,37**	1.34228519291631e-07		**0,06**	0.427981985859474
IL22RA1	ENSG00000142677		**-0,30**	3.08367034022956e-05		**-0,48**	2.87726914589897e-12
IL23A	ENSG00000110944		**-0,03**	0.644645797902959		**-0,45**	9.67115060054828e-11
IL23R	ENSG00000162594		**-0,22**	0.00257454489699698		**0,22**	0.00182712979345897
IL24	ENSG00000162892		**-0,29**	3.79070970091223e-05		**-0,17**	0.0192959023633358
IL27RA	ENSG00000104998		**-0,10**	0.162994150074083		**-0,40**	8.86323974097409e-09
IL2RB	ENSG00000100385		**-0,18**	0.0144431332335081		**-0,26**	0.000343963819283761
IL2RG	ENSG00000147168		**-0,40**	7.15846989614708e-09		**-0,24**	0.000732077598678967
IL4R	ENSG00000077238		**-0,24**	0.000698199612902479		**-0,60**	9.01546609244505e-20
IL5RA	ENSG00000091181		**0,03**	0.700688253503954		**0,18**	0.0131308852385535
IL6	ENSG00000136244		**-0,38**	6.23851661010328e-08		**-0,54**	1.04547716421021e-15
IL6R	ENSG00000160712		**0,24**	0.00098114997789636		**0,36**	3.88431360002777e-07
IL6ST	ENSG00000134352		**-0,75**	3.44003434019804e-35		**-0,27**	0.000126298974719615
IL7	ENSG00000104432		**-0,13**	0.0689954954170196		**-0,11**	0.125212694452644
IL7R	ENSG00000168685		**-0,39**	2.05832329971e-08		**-0,28**	9.30815288694941e-05
INPP5D	ENSG00000168918		**-0,03**	0.680907530145568		**0,13**	0.0686788270498966
INPPL1	ENSG00000165458		**0,40**	8.76495288213761e-09		**-0,03**	0.68995585681913
IRAK1	ENSG00000184216		**0,50**	2.20514607971888e-13		**-0,12**	0.103457118518497
IRAK2	ENSG00000134070		**-0,35**	6.95029660032743e-07		**-0,43**	7.01973501615173e-10
IRAK3	ENSG00000090376		**-0,39**	2.47592964601667e-08		**-0,13**	0.0726301846659023
IRAK4	ENSG00000198001		**-0,47**	5.89109885371024e-12		**-0,07**	0.309402333728931
IRF1	ENSG00000125347		**-0,22**	0.00218498430516907		**-0,45**	6.8595246836266e-11
IRF2	ENSG00000168310		**-0,53**	2.64551578741172e-15		**-0,12**	0.0860556588295567
IRF2BP1	ENSG00000170604		**0,72**	9.17104891882578e-32		**0,10**	0.183485049010059
IRF2BP2	ENSG00000168264		**0,53**	1.68888176200459e-15		**0,24**	0.000896726432369371
IRF2BPL	ENSG00000119669		**0,74**	6.00356439834529e-34		**0,10**	0.191013699605074
IRF3	ENSG00000126456		**0,62**	1.55820327947092e-21		**-0,13**	0.0674161842953076
IRF4	ENSG00000137265		**-0,37**	1.1784521145191e-07		**-0,50**	3.2774625407313e-13
IRF5	ENSG00000128604		**0,52**	6.57680600283766e-15		**-0,13**	0.0848245154461347
IRF6	ENSG00000117595		**-0,56**	4.31345058997722e-17		**0,07**	0.317195808715754
IRF7	ENSG00000185507		**0,66**	2.2146313704963e-25		**-0,12**	0.108319133878486
IRF8	ENSG00000140968		**-0,17**	0.0189482704504927		**-0,20**	0.00606151359148182
IRF9	ENSG00000213928		**-0,01**	0.927355270888246		**-0,11**	0.13027341850353
ITCH	ENSG00000078747		**-0,60**	4.22823020929935e-20		**0,00**	0.995443777689389
ITGAM	ENSG00000169896		**-0,25**	0.000595098079308897		**-0,39**	3.24725303031618e-08
ITGB2	ENSG00000160255		**0,11**	0.115161320399373		**-0,07**	0.302669643195739
ITK	ENSG00000113263		**-0,35**	9.48872696010573e-07		**-0,36**	2.7307851825753e-07
ITPR1	ENSG00000150995		**-0,19**	0.00737111439320625		**0,36**	2.55509115938012e-07
ITPR2	ENSG00000123104		**-0,43**	3.87773800527843e-10		**-0,02**	0.777396709371253
ITPR3	ENSG00000096433		**0,52**	1.4137230967383e-14		**0,03**	0.644539315465679
JAK1	ENSG00000162434		**-0,52**	1.21965899014069e-14		**-0,17**	0.0181285138677532
JAK2	ENSG00000096968		**-0,21**	0.00293620050676593		**0,31**	1.15950395778076e-05
JAK3	ENSG00000105639		**0,16**	0.0254759799124599		**-0,12**	0.10278525129845
JUN	ENSG00000177606		**0,52**	1.31951739040314e-14		**-0,14**	0.0515921985821478

JUNB	ENSG00000171223	**0,64**	7.67212510391768e-24	**-0,12**	0.102603877780216
JUND	ENSG00000130522	**0,63**	3.82201233809119e-22	**-0,04**	0.545072472364982
KIT	ENSG00000157404	**-0,32**	6.06911145992919e-06	**-0,14**	0.0548835554504869
KITLG	ENSG00000049130	**-0,59**	2.75247625001181e-19	**-0,24**	0.000939067200012976
L1CAM	ENSG00000198910	**0,66**	3.85954487597072e-25	**0,52**	2.3287909822314e-14
LAG3	ENSG00000089692	**0,26**	0.000351965640202587	**-0,11**	0.130712200205277
LAT	ENSG00000213658	**0,43**	3.55290622637628e-10	**-0,19**	0.00780893729859522
LAT2	ENSG00000086730	**0,15**	0.0396274372030864	**0,28**	7.09962378807394e-05
LBP	ENSG00000129988	**-0,31**	1.41177897209494e-05	**-0,36**	4.62292072031325e-07
LCK	ENSG00000182866	**-0,15**	0.0353462625797426	**-0,23**	0.00136232130345569
LCP1	ENSG00000136167	**-0,60**	1.10630737088561e-19	**-0,32**	8.83239700673441e-06
LCP2	ENSG00000043462	**-0,33**	3.3978182306189e-06	**-0,01**	0.887113276895679
LEPR	ENSG00000116678	**-0,13**	0.0727229845871705	**0,44**	2.83709669415057e-10
LGALS1	ENSG00000100097	**0,18**	0.0136906312741114	**-0,06**	0.446393595057236
LGALS3	ENSG00000131981	**-0,04**	0.625148796013988	**-0,54**	1.39997868335968e-15
LGALS9	ENSG00000168961	**-0,24**	0.000649493932591455	**-0,41**	6.19081511091341e-09
LGMN	ENSG00000100600	**-0,06**	0.372975840224934	**0,25**	0.000634980657576646
LIF	ENSG00000128342	**-0,05**	0.482819781309866	**-0,45**	6.38650230162e-11
LIFR	ENSG00000113594	**-0,64**	2.66231348605376e-23	**-0,42**	1.55726367986194e-09
LILRB1	ENSG00000104972	**-0,03**	0.632167860358662	**-0,05**	0.523110539175119
LITAF	ENSG00000189067	**-0,54**	1.51346752468505e-15	**-0,48**	3.61387449261535e-12
LSP1	ENSG00000130592	**0,37**	2.01883529356566e-07	**-0,11**	0.113768012545791
LY86	ENSG00000112799	**-0,27**	0.000120473749485373	**-0,05**	0.512495295967913
LY96	ENSG00000154589	**-0,22**	0.00195846263185404	**-0,13**	0.0753037824128098
LYN	ENSG00000254087	**-0,60**	9.33987560128866e-20	**-0,54**	1.34097702010841e-15
MALT1	ENSG00000172175	**-0,49**	6.06702914488493e-13	**-0,33**	2.59213679360673e-06
MAP2K1	ENSG00000169032	**-0,42**	1.0089549299884e-09	**-0,13**	0.0641132819324937
MAP2K2	ENSG00000126934	**0,66**	1.17674104864783e-25	**0,00**	0.958168479966848
MAP2K3	ENSG00000034152	**0,31**	1.74885548526188e-05	**-0,30**	2.24391426857431e-05
MAP2K4	ENSG00000065559	**-0,62**	1.34976778554994e-21	**-0,07**	0.364097650238115
MAP2K6	ENSG00000108984	**-0,40**	1.53752344947262e-08	**0,19**	0.00843758230584174
MAP2K7	ENSG00000076984	**0,64**	3.49620863487539e-23	**-0,10**	0.152819022261597
MAP3K1	ENSG00000095015	**-0,59**	2.14954752454161e-19	**-0,38**	5.75968519951562e-08
MAP3K14	ENSG00000006062	**0,46**	1.93455883792861e-11	**-0,23**	0.00161677069109988
MAP3K7	ENSG00000135341	**-0,35**	6.39003437413233e-07	**-0,01**	0.867092351786451
MAP3K8	ENSG00000107968	**-0,24**	0.000771602514091049	**-0,43**	5.60752233400665e-10
MAP4K1	ENSG00000104814	**0,19**	0.00926934558022212	**-0,16**	0.0250800474502605
MAPK1	ENSG00000100030	**-0,60**	2.99200936810869e-20	**0,08**	0.251282465884628
MAPK10	ENSG00000109339	**0,00**	0.962978066789964	**0,63**	1.24976684735071e-22
MAPK11	ENSG00000185386	**0,64**	1.33530520718186e-23	**0,08**	0.268922126268205
MAPK12	ENSG00000188130	**0,70**	3.25251537512553e-29	**0,02**	0.772127715624114
MAPK13	ENSG00000156711	**0,42**	2.1806270771068e-09	**0,09**	0.206238498899922
MAPK14	ENSG00000112062	**-0,44**	1.17566206635571e-10	**0,05**	0.507090038075621
MAPK3	ENSG00000102882	**0,36**	2.87917061872028e-07	**0,30**	2.0009180219269e-05
MAPK7	ENSG00000166484	**0,49**	6.3630284280541e-13	**-0,14**	0.046384182889557
MAPK8	ENSG00000107643	**-0,56**	2.23804290203971e-17	**-0,30**	1.96903338705634e-05
MAPK9	ENSG00000050748	**-0,46**	1.64214884535091e-11	**0,20**	0.00516805948761377
MAPKAPK2	ENSG00000162889	**0,09**	0.217208145936071	**-0,23**	0.00163436054379597
MAPKAPK3	ENSG00000114738	**-0,40**	9.98193686266164e-09	**-0,30**	2.70847438766614e-05
MAVS	ENSG00000088888	**0,04**	0.579983207901823	**0,02**	0.83104014829524
MCL1	ENSG00000143384	**-0,62**	2.55283843795004e-21	**-0,52**	1.22181414236806e-14
MCU	ENSG00000156026	**-0,46**	2.78873038718332e-11	**-0,12**	0.0917992486347978
MECOM	ENSG00000085276	**-0,54**	6.6927597721106e-16	**-0,39**	3.04494273552936e-08
MEF2A	ENSG00000068305	**-0,12**	0.0970125648247134	**0,36**	3.21453133657993e-07
MEF2C	ENSG00000081189	**-0,32**	5.17879565738277e-06	**0,07**	0.362073670060379
MEFV	ENSG00000103313	**0,09**	0.192965919970094	**0,09**	0.230447139700035
MFN1	ENSG00000171109	**-0,67**	4.72366636761926e-26	**-0,20**	0.00445902590122448
MFN2	ENSG00000116688	**-0,48**	1.97963303230989e-12	**0,07**	0.324564401772141

MKL1	ENSG00000196588	**0,12**	0.0901771134989606	**-0,29**	4.45200356614047e-05
MLANA	ENSG00000120215	**-0,17**	0.0185560094806087	**0,08**	0.275706555347608
MLST8	ENSG00000167965	**0,70**	6.58810809014195e-30	**0,05**	0.49115449048096
MPL	ENSG00000117400	**0,24**	0.000986450382894598	**0,17**	0.0198430138589992
MPO	ENSG00000005381	**0,11**	0.127206685393783	**-0,08**	0.265453735207945
MTOR	ENSG00000198793	**-0,46**	1.39801399115379e-11	**0,03**	0.700528947537056
MYB	ENSG00000118513	**-0,26**	0.000353500865171918	**-0,33**	2.45512804537522e-06
MYC	ENSG00000136997	**-0,17**	0.0168645538571789	**-0,49**	3.93334794486251e-13
MYD88	ENSG00000172936	**-0,63**	1.30792485750974e-22	**-0,47**	7.90972736361841e-12
NAIP	ENSG00000249437	**-0,15**	0.0414780495721263	**0,22**	0.00234598439617827
NAMPT	ENSG00000105835	**-0,55**	2.80614905607492e-16	**-0,58**	1.57004054808471e-18
NCAM1	ENSG00000149294	**-0,05**	0.490859806182678	**0,47**	1.21484470358627e-11
NCF1	ENSG00000158517	**0,10**	0.178866957148361	**-0,10**	0.156594679769947
NCF4	ENSG00000100365	**0,15**	0.0389342502295355	**-0,26**	0.000310496186227933
NCK1	ENSG00000158092	**-0,49**	6.53802239141109e-13	**-0,13**	0.0666609316618826
NDRG1	ENSG00000104419	**0,07**	0.34285519864896	**0,10**	0.182211700758676
NEK7	ENSG00000151414	**-0,50**	3.30675191180202e-13	**-0,09**	0.215065227444383
NFATC1	ENSG00000131196	**0,05**	0.485414685834601	**-0,50**	9.96576792227391e-14
NFATC2	ENSG00000101096	**-0,14**	0.0555556865480954	**-0,05**	0.453747745548718
NFATC3	ENSG00000072736	**-0,59**	2.06247756182526e-19	**0,06**	0.448382179739784
NFATC4	ENSG00000100968	**0,31**	1.1832082113729e-05	**-0,01**	0.883659976472231
NFIL3	ENSG00000165030	**-0,50**	1.03858930611913e-13	**-0,39**	2.45598804160981e-08
NFKB1	ENSG00000109320	**-0,59**	1.41337345847154e-19	**-0,40**	1.27178951836757e-08
NFKB2	ENSG00000077150	**0,22**	0.00270211457539767	**-0,42**	1.95754158193685e-09
NFKBIA	ENSG00000100906	**-0,22**	0.00228519241789342	**-0,59**	5.63470401469128e-19
NFKBIB	ENSG00000104825	**0,39**	3.51981854643213e-08	**-0,19**	0.00737618848454116
NFKBIE	ENSG00000146232	**0,27**	0.00017205593019912	**-0,46**	3.34329888230106e-11
NFKBIZ	ENSG00000144802	**-0,27**	0.000209260576551246	**-0,52**	1.59179814485082e-14
NLRC3	ENSG00000167984	**0,51**	3.59989646250484e-14	**0,06**	0.449225544802749
NLRC4	ENSG00000091106	**-0,25**	0.00041205306661544	**-0,03**	0.656870791530623
NLRC5	ENSG00000140853	**0,25**	0.000597976325727681	**-0,31**	1.17107390335828e-05
NLRP1	ENSG00000091592	**0,77**	3.79719345912581e-38	**0,37**	1.09255412687703e-07
NLRP11	ENSG00000179873	**-0,23**	0.00149190925976655	**0,29**	3.89786051491714e-05
NLRP14	ENSG00000158077	**-0,11**	0.134218056054006	**-0,03**	0.701693109882654
NLRP2	ENSG00000022556	**-0,19**	0.00927056750753622	**-0,06**	0.376765102136367
NLRP2B	ENSG00000215174	**0,17**	0.0209262564937694	**0,07**	0.357116883469357
NLRP3	ENSG00000162711	**-0,10**	0.19094381413985	**0,13**	0.0798381199584165
NLRP9	ENSG00000185792	**0,03**	0.671254925883758	**-0,18**	0.0113685271077864
NLRX1	ENSG00000160703	**0,62**	2.53561542128123e-21	**0,06**	0.393120541657589
NOD1	ENSG00000106100	**0,31**	1.28556623781801e-05	**-0,16**	0.0233950677883959
NOD2	ENSG00000167207	**-0,16**	0.0271555303549858	**-0,40**	1.19593954178795e-08
NOS2	ENSG00000007171	**0,04**	0.553775969547528	**-0,01**	0.849052646270222
NQO1	ENSG00000181019	**-0,16**	0.0236421302308966	**0,06**	0.432696285529527
NR1D1	ENSG00000126368	**0,42**	1.32528821776255e-09	**-0,11**	0.128289219760083
NR4A1	ENSG00000123358	**0,41**	3.24061388017239e-09	**-0,16**	0.0250101211685672
NT5E	ENSG00000135318	**-0,42**	9.43901683342149e-10	**-0,29**	3.50539193809275e-05
OAS1	ENSG00000089127	**-0,15**	0.0328201484234877	**0,05**	0.480262960349265
OAS2	ENSG00000111335	**-0,24**	0.000864956449482526	**-0,08**	0.252654097541656
OAS3	ENSG00000111331	**-0,26**	0.000330722148169071	**-0,14**	0.0474655004707886
OSM	ENSG00000099985	**0,24**	0.000652799939162351	**-0,25**	0.000431003212695394
OSMR	ENSG00000145623	**-0,65**	2.03919984970206e-24	**-0,60**	7.58445286554061e-20
OTUD5	ENSG00000068308	**0,29**	6.14263040136555e-05	**0,01**	0.87548682456972
OTULIN	ENSG00000154124	**-0,56**	2.49292618845894e-17	**-0,37**	1.25137464427724e-07
P2RX7	ENSG00000089041	**-0,33**	3.85496202626781e-06	**-0,15**	0.0408852116686779
PANX1	ENSG00000110218	**-0,63**	2.26924415522877e-22	**-0,34**	1.65931159458679e-06
PAWR	ENSG00000177425	**-0,51**	8.34928734754634e-14	**-0,34**	1.93466025983374e-06
PAX5	ENSG00000196092	**0,20**	0.00579142192396889	**0,06**	0.409230878578268
PBX1	ENSG00000185630	**-0,66**	4.01668012801769e-25	**-0,13**	0.0842075528725004

PDCD1LG2	ENSG00000197646	**-0,37**	1.27447973188059e-07	**-0,04**	0.562863902274061
PDGFA	ENSG00000197461	**0,04**	0.626089165063433	**-0,45**	4.13858779805118e-11
PDGFB	ENSG00000100311	**0,19**	0.0081006964117123	**-0,15**	0.0411937918306799
PDGFRA	ENSG00000134853	**-0,34**	1.69482491096601e-06	**-0,17**	0.0201033963368846
PDGFRB	ENSG00000113721	**0,02**	0.827196848427399	**0,19**	0.00836832573983567
PDLIM2	ENSG00000120913	**0,74**	3.45372262845816e-34	**0,14**	0.057930476284971
PDXDC2P	ENSG00000255185	**0,29**	6.22932103964344e-05	**-0,12**	0.105482149605217
PDXDC2P	ENSG00000196696	**0,26**	0.000215554292578816	**-0,11**	0.125199982222296
PELI1	ENSG00000197329	**-0,38**	4.34016196806095e-08	**-0,13**	0.0785560525101174
PELI2	ENSG00000139946	**-0,33**	3.06310669946723e-06	**-0,24**	0.000705477624879387
PELI3	ENSG00000174516	**0,83**	1.58777613151634e-49	**0,50**	2.80364309678431e-13
PIAS1	ENSG00000033800	**-0,28**	9.55460502211832e-05	**0,38**	4.92018850220099e-08
PIAS2	ENSG00000078043	**-0,26**	0.00022405124725867	**0,28**	0.000115020841347794
PIAS3	ENSG00000131788	**0,19**	0.00845215288768675	**0,13**	0.0730097499669709
PIAS4	ENSG00000105229	**0,79**	2.5433816202907e-41	**0,16**	0.0283593171943679
PIK3C3	ENSG00000078142	**-0,65**	2.72676581426382e-24	**-0,08**	0.259187632633284
PIK3CA	ENSG00000121879	**-0,67**	2.39529234700326e-26	**-0,17**	0.0210010819676031
PIK3CB	ENSG00000051382	**-0,63**	4.05405996223875e-22	**-0,20**	0.00456660588519594
PIK3CD	ENSG00000171608	**0,29**	5.85616427793295e-05	**-0,34**	1.86148637990212e-06
PIK3CG	ENSG00000105851	**-0,23**	0.00110467851680458	**0,09**	0.192800970725085
PIK3R1	ENSG00000145675	**-0,70**	4.94075866857068e-29	**-0,09**	0.19404408185396
PIK3R2	ENSG00000105647	**0,77**	6.52792398222713e-39	**0,42**	2.10515977066689e-09
PIK3R3	ENSG00000117461	**-0,05**	0.526593151012579	**0,55**	1.10202969066579e-16
PIK3R4	ENSG00000196455	**-0,51**	8.92940035618747e-14	**0,18**	0.0118004048728248
PIK3R5	ENSG00000141506	**-0,26**	0.00030486410290903	**-0,22**	0.00229483366082675
PIM1	ENSG00000137193	**-0,17**	0.0224194258620482	**-0,53**	1.66038013424789e-15
PIM2	ENSG00000102096	**0,22**	0.00258716519275119	**0,29**	4.84964694210049e-05
PIM3	ENSG00000198355	**0,63**	8.71485578755993e-23	**-0,11**	0.144870122108829
PIR	ENSG00000087842	**0,09**	0.212714082411612	**0,35**	9.68201056300833e-07
PLCB1	ENSG00000182621	**-0,35**	8.1419133064092e-07	**0,24**	0.000655213260053309
PLCB2	ENSG00000137841	**0,02**	0.808882447332441	**-0,08**	0.257671521303034
PLCB3	ENSG00000149782	**0,35**	5.27778453002387e-07	**-0,21**	0.00313949767236579
PLCB4	ENSG00000101333	**0,20**	0.00662714513277555	**0,62**	2.48298916295547e-21
PLCG2	ENSG00000197943	**-0,16**	0.0230647123569113	**0,20**	0.00670425002113378
PLK1	ENSG00000166851	**0,34**	1.50673321112083e-06	**0,07**	0.338487565433572
POLR2J4	ENSG00000272655	**0,28**	9.52155431834251e-05	**-0,04**	0.542454108967578
POLR2J4	ENSG00000214783	**0,16**	0.0276471738470529	**0,01**	0.87623141564244
PPIA	ENSG00000196262	**-0,35**	7.7201959368117e-07	**-0,35**	7.925141630842e-07
PPP2CA	ENSG00000113575	**-0,41**	2.75800443762301e-09	**-0,09**	0.232332134389138
PPP2CB	ENSG00000104695	**-0,29**	5.60647540573015e-05	**0,32**	8.87827848932038e-06
PPP2R1A	ENSG00000105568	**0,21**	0.00411510699179665	**-0,04**	0.595479890872361
PPP2R1B	ENSG00000137713	**-0,56**	2.64266607345147e-17	**0,07**	0.329253344289962
PPP2R2A	ENSG00000221914	**-0,59**	2.19129426250063e-19	**-0,22**	0.00211556723369208
PPP2R2B	ENSG00000156475	**-0,01**	0.896499500443703	**0,37**	1.66536338900677e-07
PPP2R2C	ENSG00000074211	**0,36**	2.19762143759916e-07	**0,70**	7.34023567340372e-30
PPP2R2D	ENSG00000175470	**-0,02**	0.753234688162031	**-0,27**	0.000141891395998047
PPP2R5D	ENSG00000112640	**-0,59**	5.11323513896183e-19	**-0,05**	0.533061354004436
PPP3CA	ENSG00000138814	**-0,63**	3.47699012017508e-22	**0,05**	0.457649845983456
PPP3CB	ENSG00000107758	**-0,21**	0.00431500877194442	**0,47**	7.94593162044097e-12
PPP3CC	ENSG00000120910	**-0,18**	0.0146576693605873	**-0,11**	0.127603654675323
PRDM1	ENSG00000057657	**-0,14**	0.0468846090467221	**-0,03**	0.718980336151392
PRKCD	ENSG00000163932	**-0,35**	8.07477316843992e-07	**-0,18**	0.0106597716713838
PRKCQ	ENSG00000065675	**-0,39**	2.69572524203856e-08	**0,08**	0.283721721238615
PRLR	ENSG00000113494	**-0,35**	4.78539784286799e-07	**0,25**	0.000489188291246032
PSIP1	ENSG00000164985	**0,12**	0.107902057450008	**0,51**	3.12756893040823e-14
PSMB8	ENSG00000204264	**-0,61**	3.10896602614374e-21	**-0,37**	1.56551206915383e-07
PSTPIP1	ENSG00000140368	**0,21**	0.00308693426500499	**-0,18**	0.0150606510591915
PTGS1	ENSG00000095303	**-0,29**	5.40111873845437e-05	**-0,04**	0.538575963245348

PTGS2	ENSG00000073756		**-0,37**	1.06202411715418e-07		**-0,41**	5.66990919222164e-09
PTK2B	ENSG00000120899		**0,18**	0.0104280244768455		**-0,09**	0.227088637359813
PTPN11	ENSG00000179295		**-0,44**	2.83742915818007e-10		**0,19**	0.00849151413351793
PTPN2	ENSG00000175354		**-0,40**	8.0158053878309e-09		**-0,56**	7.30794410228303e-17
PTPN22	ENSG00000134242		**-0,18**	0.011600198237508		**-0,37**	1.18438543700136e-07
PTPN4	ENSG00000088179		**-0,21**	0.00283288659587911		**0,44**	1.43557137760863e-10
PTPN6	ENSG00000111679		**0,28**	9.07159554868796e-05		**-0,09**	0.193182499885867
PTPRC	ENSG00000081237		**-0,40**	1.48074898772267e-08		**-0,12**	0.0917501247852444
PVR	ENSG00000073008		**-0,32**	5.0344876713875e-06		**-0,04**	0.561130995151631
PYCARD	ENSG00000103490		**0,50**	9.96104671154407e-14		**-0,16**	0.0309530348574785
RABGEF1	ENSG00000154710		**-0,49**	8.24379333868165e-13		**-0,16**	0.0278681982138999
RAC1	ENSG00000136238		**-0,57**	4.08102412241896e-18		**-0,25**	0.000530049895111784
RAF1	ENSG00000132155		**-0,63**	2.07438219146617e-22		**-0,58**	2.05221822580788e-18
RAG1	ENSG00000166349		**-0,45**	6.08272380941514e-11		**-0,16**	0.0321046862483524
RBCK1	ENSG00000125826		**0,71**	6.96471927151766e-31		**-0,07**	0.365753386050884
RBSN	ENSG00000131381		**-0,46**	1.86209090958448e-11		**-0,18**	0.0150670795713591
REL	ENSG00000162924		**-0,52**	7.85048830110015e-15		**-0,14**	0.0478115197701923
RELA	ENSG00000173039		**0,33**	2.60509100663762e-06		**-0,32**	4.81257582740091e-06
RELB	ENSG00000104856		**0,34**	1.49315930795871e-06		**-0,40**	1.15561076208071e-08
RGS5	ENSG00000232995		**-0,20**	0.00511519707968173		**0,20**	0.00501735236093705
RGS5	ENSG00000143248		**-0,07**	0.322589926695806		**0,27**	0.000136253917536132
RHOA	ENSG00000067560		**-0,62**	6.80393236968241e-22		**-0,14**	0.046709148538254
RIPK1	ENSG00000137275		**-0,55**	8.4800183856022e-17		**-0,05**	0.461793480056313
RIPK2	ENSG00000104312		**-0,40**	7.76508291623069e-09		**-0,59**	4.30641191933855e-19
RIPK3	ENSG00000129465		**-0,20**	0.00530966421272976		**-0,42**	1.15219531942584e-09
RIPK4	ENSG00000183421		**0,19**	0.00701996578783995		**-0,39**	2.91388919950574e-08
RNASEL	ENSG00000135828		**-0,23**	0.00109628575311977		**0,14**	0.060581002434791
RNF216	ENSG00000011275		**0,32**	6.44379989509249e-06		**0,33**	2.97326583175837e-06
RNF31	ENSG00000092098		**0,46**	1.53494275615763e-11		**-0,10**	0.150092128414363
RNF41	ENSG00000181852		**-0,56**	6.53825979785689e-17		**0,20**	0.0068272826776972
RPS27A	ENSG00000143947		**-0,52**	1.29280938389443e-14		**-0,34**	1.10727580600848e-06
RPS6KA1	ENSG00000117676		**-0,21**	0.00328909032437553		**-0,31**	1.20130711451515e-05
RPS6KA2	ENSG00000071242		**-0,16**	0.031317578899912		**-0,32**	5.31640212754824e-06
RPS6KA3	ENSG00000177189		**-0,41**	3.9799424436064e-09		**0,12**	0.0850318783976356
RPS6KA5	ENSG00000100784		**-0,44**	2.27624067378863e-10		**-0,45**	9.60597349232598e-11
RSAD2	ENSG00000134321		**-0,22**	0.00234969317224505		**-0,19**	0.00789868450016011
RUNX1	ENSG00000159216		**-0,30**	3.01060398236867e-05		**-0,43**	5.66165960110254e-10
RUNX1T1	ENSG00000079102		**0,17**	0.0222667162006569		**0,62**	8.90606786893307e-22
S100A1	ENSG00000160678		**-0,04**	0.544275767089726		**-0,48**	3.30346661762165e-12
S100A8	ENSG00000143546		**-0,31**	1.25854553582187e-05		**-0,22**	0.00253496444159315
S100A9	ENSG00000163220		**-0,20**	0.00505988928443025		**-0,27**	0.000163823407430586
S100B	ENSG00000160307		**-0,12**	0.104800630357125		**0,24**	0.000667004503289965
SAA1	ENSG00000173432		**-0,20**	0.00533019861105571		**-0,17**	0.0223168546849501
SARM1	ENSG00000004139		**0,11**	0.123602079181675		**0,63**	2.84561714161296e-22
SERPINA3	ENSG00000273259		**-0,42**	1.42104706249298e-09		**-0,43**	3.27873039927722e-10
SERPINA3	ENSG00000196136		**-0,42**	1.56808288090945e-09		**-0,43**	3.22480254445208e-10
SERPINE1	ENSG00000106366		**-0,09**	0.219204800193226		**-0,03**	0.69413671922963
SFTPA2	ENSG00000185303		**-0,04**	0.556341807577317		**-0,06**	0.406750710791915
SFTPD	ENSG00000133661		**0,52**	1.46662444808833e-14		**0,26**	0.000358339964913249
SH3KBP1	ENSG00000147010		**0,01**	0.861054227655836		**0,48**	3.3164529146065e-12
SHARPIN	ENSG00000179526		**0,71**	2.07361837377747e-30		**-0,03**	0.649870036329941
SIGIRR	ENSG00000185187		**0,63**	1.19898285030601e-22		**-0,14**	0.0466520513360271
SIRPA	ENSG00000198053		**-0,28**	0.00010853234303029		**-0,51**	4.221396093417e-14
SIVA1	ENSG00000184990		**0,57**	5.10946045058438e-18		**-0,09**	0.233043809474865
SKP1	ENSG00000113558		**-0,62**	1.19710147539641e-21		**-0,09**	0.199328127159309
SMAD2	ENSG00000175387		**-0,62**	2.55757344303848e-21		**0,01**	0.901783263914251
SMAD3	ENSG00000166949		**-0,52**	1.32561383824166e-14		**-0,68**	6.22028241866963e-27
SMAD6	ENSG00000137834		**0,56**	3.36394232638854e-17		**-0,05**	0.518355878742066

SNORA31	ENSG00000253051	**0,08**	0.247884241060087	**-0,36**	2.27528053925494e-07
SNORA31	ENSG00000199477	**0,14**	0.0458869844227978	**-0,03**	0.665768994204159
SOCS1	ENSG00000185338	**0,62**	1.13362876375065e-21	**-0,11**	0.135148843402378
SOCS2	ENSG00000120833	**0,17**	0.0169989833644122	**-0,07**	0.325388859958781
SOCS3	ENSG00000184557	**0,24**	0.000917143429051332	**-0,39**	1.89577019821364e-08
SOCS4	ENSG00000180008	**-0,42**	1.09908953291492e-09	**0,15**	0.0337534242591804
SOCS5	ENSG00000171150	**-0,34**	1.16229795500811e-06	**0,28**	7.78587049118092e-05
SOCS6	ENSG00000170677	**-0,45**	5.81029926580992e-11	**0,24**	0.000833671270241291
SOS1	ENSG00000115904	**-0,51**	7.07731058980343e-14	**0,18**	0.013979692177311
SOS2	ENSG00000100485	**-0,30**	3.29676387364346e-05	**0,23**	0.00166593904360047
SPATA13	ENSG00000228741	**-0,11**	0.114114781230806	**-0,49**	6.85317565706166e-13
SPATA13	ENSG00000182957	**-0,25**	0.000460265723198588	**0,19**	0.00703600084024159
SPI1	ENSG00000066336	**0,49**	9.08606474832705e-13	**-0,10**	0.173284886774545
SPIB	ENSG00000269404	**-0,18**	0.0115329434489913	**-0,23**	0.00116526772643817
SPP1	ENSG00000118785	**-0,42**	9.37489933209361e-10	**-0,24**	0.00104613032082141
SQSTM1	ENSG00000161011	**0,34**	1.0468206756078e-06	**-0,26**	0.000240691537685364
SRC	ENSG00000197122	**0,08**	0.262402472097997	**-0,39**	2.39005445263932e-08
STAM	ENSG00000136738	**-0,57**	9.13107670527743e-18	**-0,02**	0.816272844952455
STAM2	ENSG00000115145	**-0,60**	2.25232612953512e-20	**-0,03**	0.729233553337462
STAT1	ENSG00000115415	**-0,46**	1.4084182737644e-11	**0,08**	0.270253906811317
STAT2	ENSG00000170581	**-0,39**	1.74298679914003e-08	**0,06**	0.42665854407596
STAT3	ENSG00000168610	**-0,46**	1.24763123118793e-11	**0,13**	0.0793916043044484
STAT4	ENSG00000138378	**-0,02**	0.759118455330614	**0,47**	5.63943268292532e-12
STAT5A	ENSG00000126561	**-0,17**	0.0193706168094797	**-0,35**	8.63634668143367e-07
STAT5B	ENSG00000173757	**-0,08**	0.246635997702101	**0,18**	0.0130559703411971
STAT6	ENSG00000166888	**-0,25**	0.000617279201061092	**-0,34**	1.44452420385678e-06
STK17B	ENSG00000081320	**-0,67**	4.88516918811407e-26	**-0,35**	5.42276731686786e-07
SUGT1	ENSG00000165416	**-0,45**	1.0266721425207e-10	**0,20**	0.00462187129620602
SWAP70	ENSG00000133789	**-0,55**	3.07766839648613e-16	**-0,27**	0.000206665166274767
SYK	ENSG00000165025	**-0,56**	2.27291333125735e-17	**-0,24**	0.000919588522465793
TAB1	ENSG00000100324	**0,63**	1.28386119895019e-22	**0,03**	0.66563699546499
TAB2	ENSG00000055208	**-0,41**	5.05079019304517e-09	**0,04**	0.573441685779374
TAB3	ENSG00000157625	**-0,52**	1.75063160477548e-14	**0,01**	0.939505621197513
TAL1	ENSG00000162367	**-0,07**	0.37045006164026	**0,07**	0.349444553036844
TANK	ENSG00000136560	**-0,76**	7.68308912814543e-38	**-0,31**	1.21568714013484e-05
TAP1	ENSG00000168394	**-0,42**	1.73146238727583e-09	**-0,44**	2.13727148818295e-10
TAP2	ENSG00000204267	**-0,23**	0.00158640560243182	**-0,34**	1.24471165672954e-06
TAX1BP1	ENSG00000106052	**-0,55**	1.28351384299721e-16	**-0,07**	0.328172593866926
TBC1D26	ENSG00000255104	**-0,65**	2.55270312189865e-24	**-0,20**	0.00536225046447314
TBC1D26	ENSG00000214946	**0,10**	0.154786928924414	**-0,24**	0.00099466939695812
TBK1	ENSG00000183735	**-0,29**	3.91972671249438e-05	**-0,17**	0.0192847214049868
TBKBP1	ENSG00000198933	**0,64**	5.27569005744411e-23	**0,00**	0.946312658712726
TCF12	ENSG00000140262	**-0,69**	8.56951339569049e-29	**-0,29**	5.87131551934035e-05
TCF3	ENSG00000071564	**0,66**	5.76092112247012e-25	**-0,11**	0.145046310025324
TEC	ENSG00000135605	**0,04**	0.620873521725902	**0,27**	0.000198382251602702
TGFB1	ENSG00000105329	**0,55**	1.32308433819893e-16	**0,01**	0.897066530135416
TGFB2	ENSG00000092969	**-0,54**	9.97863508641403e-16	**-0,46**	2.26000359991139e-11
TGFB3	ENSG00000119699	**-0,24**	0.000852620116120712	**-0,04**	0.609803092435416
TGFBR1	ENSG00000106799	**-0,67**	1.20734362266116e-26	**-0,20**	0.00549665146022675
TGFBR2	ENSG00000163513	**-0,65**	1.38394975434637e-24	**-0,46**	2.20891995244439e-11
THPO	ENSG00000090534	**-0,15**	0.0443482032489941	**-0,23**	0.00149823615820835
TICAM1	ENSG00000127666	**0,63**	1.40496099616993e-22	**-0,10**	0.149260318141619
TICAM2	ENSG00000243414	**0,06**	0.413887218692825	**0,09**	0.22107127916853
TIFA	ENSG00000145365	**-0,55**	1.91314569465526e-16	**-0,47**	1.07063946067422e-11
TIRAP	ENSG00000150455	**-0,24**	0.00087036894631872	**0,11**	0.12756277882149
TLR1	ENSG00000174125	**-0,57**	6.01430176187931e-18	**-0,43**	3.42971134324157e-10
TLR2	ENSG00000137462	**-0,48**	2.96617868898488e-12	**-0,48**	2.71067384522745e-12
TLR3	ENSG00000164342	**-0,47**	4.00939827290733e-12	**-0,39**	2.31978643767263e-08

TLR4	ENSG00000136869	**-0,43**	7.82742680718726e-10	**-0,07**	0.357624727027409
TLR5	ENSG00000187554	**-0,26**	0.00029403815219429	**0,18**	0.0126212081739423
TLR6	ENSG00000174130	**-0,50**	1.11449961999442e-13	**-0,30**	2.30339759335871e-05
TLR7	ENSG00000196664	**-0,13**	0.0772145115191967	**0,21**	0.00436660461831649
TLR8	ENSG00000101916	**-0,28**	8.38399939393977e-05	**-0,04**	0.553864088023912
TLR9	ENSG00000239732	**0,65**	1.96293418717653e-24	**-0,09**	0.22292155485444
TMED7	ENSG00000134970	**-0,39**	3.80253336262601e-08	**0,09**	0.227115529052119
TMEM173	ENSG00000184584	**-0,11**	0.11548464364466	**-0,11**	0.123682115112946
TMEM256-PLSCR3	ENSG00000262481	**0,57**	7.47453548257485e-18	**-0,08**	0.279757496240658
TNF	ENSG00000232810	**-0,17**	0.0219343961824148	**-0,33**	2.27682620675884e-06
TNFAIP3	ENSG00000118503	**-0,35**	5.59862265015102e-07	**-0,54**	1.00186587810008e-15
TNFRSF10B	ENSG00000120889	**-0,48**	1.29409164237683e-12	**-0,67**	5.94046739263928e-26
TNFRSF10D	ENSG00000173530	**-0,38**	4.88693380823324e-08	**-0,52**	7.97519224952918e-15
TNFRSF11A	ENSG00000141655	**-0,05**	0.530375301551475	**0,18**	0.0148482737165256
TNFRSF12A	ENSG00000006327	**0,44**	1.33906626529597e-10	**-0,32**	7.51562673086217e-06
TNFRSF18	ENSG00000186891	**0,53**	2.66139092572026e-15	**-0,07**	0.343631083939922
TNFRSF1A	ENSG00000067182	**0,16**	0.0249198701319338	**-0,31**	1.15091426619657e-05
TNFRSF1B	ENSG00000028137	**-0,06**	0.404942955135092	**-0,14**	0.0477794458894424
TNFRSF25	ENSG00000215788	**0,43**	6.47770095024338e-10	**-0,11**	0.114125196411887
TNFRSF4	ENSG00000186827	**0,74**	2.16882986031124e-34	**0,04**	0.571010011360076
TNFRSF9	ENSG00000049249	**-0,50**	1.0070028517877e-13	**-0,35**	8.87565160868024e-07
TNFSF11	ENSG00000120659	**-0,38**	5.60512313684955e-08	**-0,35**	7.11257109015937e-07
TNFSF12	ENSG00000239697	**0,47**	6.09118885732618e-12	**0,05**	0.534358536366696
TNFSF13B	ENSG00000102524	**0,03**	0.671849702058623	**-0,15**	0.0449051433756027
TNFSF15	ENSG00000181634	**-0,43**	5.7751199031524e-10	**-0,24**	0.000762369856125732
TNFSF4	ENSG00000117586	**-0,05**	0.513552135998122	**0,52**	2.33517396890738e-14
TNIP1	ENSG00000145901	**-0,22**	0.00215420269830205	**-0,59**	1.57503796869035e-19
TNIP2	ENSG00000168884	**0,55**	2.95516914995812e-16	**-0,21**	0.00359823075359182
TOLLIP	ENSG00000078902	**0,83**	5.81664600081825e-50	**0,27**	0.000184423083762339
TP53BP1	ENSG00000067369	**-0,20**	0.00531588663163381	**0,39**	2.75096101094694e-08
TPT1	ENSG00000133112	**-0,39**	3.31226952720264e-08	**-0,50**	1.69911809584445e-13
TRAF1	ENSG00000056558	**-0,13**	0.0707500888507634	**-0,39**	2.73710498021346e-08
TRAF2	ENSG00000127191	**0,62**	1.04382589432806e-21	**-0,03**	0.656224728116167
TRAF3	ENSG00000131323	**0,56**	1.74566636174895e-17	**0,09**	0.213743615770432
TRAF4	ENSG00000076604	**0,07**	0.313277913045204	**-0,47**	1.04511786810452e-11
TRAF5	ENSG00000082512	**-0,37**	1.13034719455547e-07	**-0,49**	6.58968530301118e-13
TRAF6	ENSG00000175104	**-0,55**	8.75714446453464e-17	**-0,03**	0.708375135881821
TRAFD1	ENSG00000135148	**-0,58**	8.92714741918826e-19	**-0,05**	0.483350418355343
TREM1	ENSG00000124731	**-0,39**	2.09092730512601e-08	**-0,22**	0.00217552454830663
TRIB2	ENSG00000071575	**-0,32**	5.05930330302539e-06	**-0,48**	1.89950400634015e-12
TRIM25	ENSG00000121060	**-0,52**	6.71996864034073e-15	**-0,21**	0.00382735405395848
TRIP6	ENSG00000087077	**0,43**	3.80635878458557e-10	**-0,33**	2.47686065145311e-06
TRPM2	ENSG00000142185	**0,67**	6.21692865447595e-26	**0,20**	0.00604580854014028
TRPM7	ENSG00000092439	**-0,38**	5.69896213563714e-08	**0,09**	0.20934193375527
TRPV2	ENSG00000187688	**-0,03**	0.713864705847724	**0,14**	0.0582557052241475
TSLP	ENSG00000145777	**-0,23**	0.00115323266128778	**-0,18**	0.0121142857078653
TXN	ENSG00000136810	**-0,49**	9.00540109003706e-13	**-0,44**	1.64371614728299e-10
TXN2	ENSG00000100348	**-0,26**	0.000244419731624454	**0,00**	0.955036399521385
TYK2	ENSG00000105397	**0,65**	1.55184202437123e-24	**-0,02**	0.769230270167196
TYROBP	ENSG00000011600	**0,36**	4.21023261915518e-07	**-0,08**	0.271242022897435
U3	ENSG00000221496	**0,34**	1.53928303647714e-06	**0,00**	0.967154102852758
U3	ENSG00000200693	**0,14**	0.0496575518679295	**0,04**	0.53664472683544
UBA52	ENSG00000221983	**-0,72**	1.86618598734546e-31	**-0,24**	0.000873939378966847
UBB	ENSG00000170315	**-0,59**	2.50119210933739e-19	**0,14**	0.0625638107445379
UBC	ENSG00000150991	**-0,56**	2.78723123981906e-17	**-0,07**	0.364084254050423
UBD	ENSG00000213886	**-0,42**	1.28937273192377e-09	**-0,29**	3.87750622045804e-05
UBE2D1	ENSG00000072401	**-0,17**	0.0223304402788047	**-0,37**	1.48904201358508e-07

UBE2D2	ENSG00000131508	**-0,12**	0.0896614230489009	**-0,02**	0.767514104162234
UBE2D3	ENSG00000109332	**-0,45**	7.80311120389263e-11	**-0,11**	0.116257490538917
UBE2N	ENSG00000177889	**-0,67**	2.73300126693736e-26	**-0,03**	0.639894938211311
UBE2V1	ENSG00000244687	**-0,76**	4.52754229365559e-37	**-0,16**	0.0243507130100157
USP15	ENSG00000135655	**-0,73**	3.24866889647988e-33	**-0,29**	4.94735408754956e-05
USP7	ENSG00000187555	**-0,38**	5.65757832222714e-08	**0,11**	0.128899796540657
VCAM1	ENSG00000162692	**-0,49**	9.16998464331095e-13	**-0,39**	1.73018586390805e-08
VDAC1	ENSG00000213585	**-0,69**	3.82012204638129e-28	**-0,25**	0.000609366405616976
VDAC2	ENSG00000165637	**-0,13**	0.0816138286832395	**-0,38**	4.87736643155976e-08
VDAC3	ENSG00000078668	**-0,57**	3.79182614906668e-18	**0,04**	0.5446635006983
VRK3	ENSG00000105053	**-0,26**	0.000311817662092865	**-0,05**	0.479118600266547
VTCN1	ENSG00000134258	**-0,40**	9.66088260352251e-09	**-0,22**	0.00215505844841412
XIAP	ENSG00000101966	**-0,59**	6.25035150733446e-19	**0,16**	0.027976270689277
XRCC5	ENSG00000079246	**-0,71**	3.34902387285549e-30	**-0,07**	0.346423064836956
ZAP70	ENSG00000115085	**0,31**	9.52484863713989e-06	**-0,18**	0.0119483164628887
ZBTB16	ENSG00000109906	**0,25**	0.000452757101184267	**-0,08**	0.287115434266541
ZBTB7B	ENSG00000160685	**0,62**	7.74804102458198e-22	**-0,08**	0.258840781459306
ZMYND11	ENSG00000015171	**-0,34**	1.53816901969717e-06	**0,38**	6.51149115865463e-08

Table S3A

MAFA and *MAFB* co-expression correlations with T1D risk genes

**Genename**	**GeneID**	**MAFA_GC**	***P*-value**	**MAFB_GC**	***P*-value**
AFF3	ENSG00000144218	**-0,02**	0.734867579441171	**0,14**	0.0617982796618209
BACH2	ENSG00000112182	**-0,29**	4.57520038667183e-05	**-0,41**	5.2898841627837e-09
C1QTNF6	ENSG00000133466	**0,43**	5.07288584424574e-10	**0,27**	0.000127948621652364
CCR5	ENSG00000160791	**-0,38**	5.16932265268277e-08	**-0,03**	0.706147121741022
CD226	ENSG00000150637	**-0,19**	0.00690819876364436	**-0,03**	0.69068251339251
CD69	ENSG00000110848	**-0,28**	0.00011151243749079	**-0,23**	0.00166527346414262
CENPW	ENSG00000203760	**-0,34**	2.02994096538379e-06	**-0,07**	0.338263538815517
CLEC16A	ENSG00000038532	**0,16**	0.0317948087309315	**0,04**	0.542532144507101
COBL	ENSG00000106078	**0,04**	0.5766368100334	**0,28**	8.63092428279222e-05
CPE	ENSG00000109472	**-0,06**	0.441702555351971	**0,59**	1.3103850390649e-19
CTRB1	ENSG00000168925	**0,46**	2.04103615890964e-11	**-0,28**	7.12248942709951e-05
CTSH	ENSG00000103811	**-0,10**	0.166599491184495	**-0,31**	9.08146686157259e-06
CYP27B1	ENSG00000111012	**-0,11**	0.136648414210512	**-0,50**	2.69966966275471e-13
DLK1	ENSG00000185559	**0,66**	2.68850311428816e-25	**0,20**	0.00579862569912101
DNAH2	ENSG00000183914	**0,07**	0.351176377225313	**-0,01**	0.922000568665814
EFR3B	ENSG00000084710	**0,02**	0.759925503857163	**0,48**	1.3867541100746e-12
ERBB3	ENSG00000065361	**-0,41**	4.66347012065983e-09	**-0,40**	1.01911321543803e-08
FAS	ENSG00000026103	**-0,50**	2.27089514974497e-13	**-0,36**	4.30367878444592e-07
FUT2	ENSG00000176920	**-0,27**	0.000126582547127163	**-0,18**	0.0144822715570176
GAB3	ENSG00000160219	**-0,47**	1.01165428250934e-11	**-0,26**	0.000299826984314779
GAD1	ENSG00000128683	**0,21**	0.00338564626367873	**-0,08**	0.290607828725846
GAD2	ENSG00000136750	**-0,02**	0.782751450965247	**0,59**	1.42128854896397e-19
GLIS3	ENSG00000107249	**-0,43**	7.7232688984128e-10	**-0,01**	0.941217812240755
GPR183	ENSG00000169508	**-0,46**	2.32200603802395e-11	**-0,26**	0.000340431078890772
GZMB	ENSG00000100453	**-0,07**	0.351670093339684	**-0,15**	0.0340925504782046
HERC2	ENSG00000128731	**0,35**	5.47413316301011e-07	**0,50**	1.2075714468256e-13

HLA-A	ENSG00000206503	**0,41**	3.17676782898581e-09	**0,05**	0.501993587554691
HLA-B	ENSG00000234745	**-0,07**	0.321245752822301	**-0,37**	1.35154815601213e-07
HLA-C	ENSG00000204525	**0,22**	0.00177092256548731	**-0,05**	0.493859111819826
HLA-DMA	ENSG00000204257	**-0,37**	1.97327915790382e-07	**-0,16**	0.0257260865572971
HLA-DMB	ENSG00000242574	**-0,23**	0.00118730016677934	**0,06**	0.39164006739694
HLA-DOA	ENSG00000204252	**-0,25**	0.000496014501624067	**-0,09**	0.200546981205146
HLA-DOB	ENSG00000241106	**-0,09**	0.221712768925515	**-0,27**	0.000127220766912069
HLA-DPA1	ENSG00000231389	**-0,33**	3.48390737616717e-06	**-0,14**	0.0625700888034012
HLA-DPB1	ENSG00000223865	**-0,14**	0.0500989017843944	**-0,29**	6.04620119758697e-05
HLA-DQA1	ENSG00000196735	**-0,35**	7.32409952502228e-07	**-0,06**	0.395299635736331
HLA-DQB1	ENSG00000179344	**-0,16**	0.0281057297508137	**-0,09**	0.220240452128911
HLA-DRA	ENSG00000204287	**-0,49**	9.0078408324313e-13	**-0,17**	0.0198061579232227
HLA-DRB1	ENSG00000196126	**-0,34**	1.84758362592658e-06	**-0,14**	0.0539708285037983
HLA-DRB5	ENSG00000198502	**-0,22**	0.00268285198387145	**-0,11**	0.127383172104309
HLA-E	ENSG00000204592	**0,16**	0.0291932833068203	**-0,28**	9.86111154029346e-05
HLA-F	ENSG00000204642	**0,48**	3.0844601875437e-12	**-0,25**	0.000591139503654884
HLA-G	ENSG00000204632	**0,05**	0.482261735708689	**-0,31**	9.9969053922563e-06
HSPD1	ENSG00000144381	**-0,42**	1.51101497137221e-09	**-0,03**	0.70674992640563
HTRA1	ENSG00000166033	**-0,16**	0.0253226996570963	**0,02**	0.779682250737062
ICA1	ENSG00000003147	**-0,29**	4.03360945480688e-05	**0,29**	3.52722991124353e-05
IFIH1	ENSG00000115267	**-0,35**	4.95295621421065e-07	**0,19**	0.0080246166595001
IKZF1	ENSG00000185811	**-0,32**	5.98788332420838e-06	**-0,15**	0.0327310047413403
IL10	ENSG00000136634	**-0,35**	6.62961598191475e-07	**-0,14**	0.0481751827565998
IL18RAP	ENSG00000115607	**0,03**	0.73091966474103	**-0,32**	6.22477610834922e-06
IL7R	ENSG00000168685	**-0,39**	2.05832329971e-08	**-0,28**	9.30815288694941e-05
INS	ENSG00000254647	**0,76**	3.28813327984766e-37	**0,11**	0.143142992730977
KIF5A	ENSG00000155980	**0,19**	0.00743295799022492	**0,49**	6.39098027833842e-13
LMO7	ENSG00000136153	**-0,49**	7.63282012964022e-13	**-0,33**	3.60720299743556e-06
LTA	ENSG00000226979	**0,11**	0.115156450378494	**-0,07**	0.32644901266702
ORMDL3	ENSG00000172057	**-0,47**	1.19126018897978e-11	**0,07**	0.365520401099593
PDXDC2P	ENSG00000196696	**0,26**	0.000215554292578816	**-0,12**	0.105482149605217
PGM1	ENSG00000079739	**-0,57**	1.41755522166158e-17	**-0,22**	0.00199817992063461
PRF1	ENSG00000180644	**-0,06**	0.431743056382847	**-0,15**	0.0436730463269114
PRKCQ	ENSG00000065675	**-0,39**	2.69572524203856e-08	**0,08**	0.283721721238615
PRKD2	ENSG00000105287	**0,50**	2.89331396118485e-13	**-0,16**	0.0241652482815126
PTPN2	ENSG00000175354	**-0,40**	8.0158053878309e-09	**-0,56**	7.30794410228303e-17
PTPN22	ENSG00000134242	**-0,18**	0.011600198237508	**-0,37**	1.18438543700136e-07
RASGRP1	ENSG00000172575	**-0,12**	0.11219567877165	**0,44**	1.24364310748378e-10
RGS1	ENSG00000090104	**-0,33**	2.57457538343008e-06	**-0,20**	0.00445367226257139
RNLS	ENSG00000184719	**-0,05**	0.529740880572392	**0,37**	1.4417227790833e-07
SH2B3	ENSG00000111252	**-0,10**	0.151496947280661	**-0,12**	0.0978361182552971
SKAP2	ENSG00000005020	**-0,40**	1.14193591355129e-08	**0,03**	0.650588172482157
SMARCE1	ENSG00000073584	**-0,44**	1.43919275793419e-10	**-0,01**	0.848390842436457
STAT4	ENSG00000138378	**-0,02**	0.759118455330614	**0,47**	5.63943268292532e-12
TAGAP	ENSG00000164691	**0,12**	0.0887068974190489	**0,05**	0.456841402755305
TNFAIP3	ENSG00000118503	**-0,35**	5.59862265015102e-07	**-0,54**	1.00186587810008e-15

TRIB2	ENSG00000071575	**-0,32**	5.05930330302539e-06	**-0,48**	1.89950400634015e-12
TYK2	ENSG00000105397	**0,65**	1.55184202437123e-24	**-0,02**	0.769230270167196

Table S3B

***MAFA* and *MAFB* co-expression correlations with T2D risk genes**

**Genename**	**GeneID**	**MAFA_GC**	***P*-value**	**MAFB_GC**	***P*-value**
ABCC8	ENSG00000006071	**0,34**	2.10330211688118e-06	**0,52**	1.28120588155402e-14
AKT1	ENSG00000142208	**0,49**	4.07315761278472e-13	**-0,12**	0.0868735039250571
CACNA1A	ENSG00000141837	**0,53**	2.21979789340497e-15	**0,53**	3.57238187784291e-15
CACNA1B	ENSG00000148408	**0,54**	1.07236808674557e-15	**0,66**	2.63860159496157e-25
CACNA1C	ENSG00000151067	**0,57**	1.57338173452952e-17	**0,67**	9.46345928324057e-26
CACNA1D	ENSG00000157388	**0,18**	0.0122445950182052	**0,63**	8.11006635367094e-23
CACNA1E	ENSG00000198216	**0,01**	0.93856452391617	**0,08**	0.250165259195648
CACNA1G	ENSG00000006283	**0,38**	6.84829917772518e-08	**0,06**	0.434378301917194
GCK	ENSG00000106633	**0,53**	1.89645691952229e-15	**0,60**	5.34597962445488e-20
GK	ENSG00000198814	**-0,50**	1.19986437672528e-13	**-0,38**	7.10036125634837e-08
HK1	ENSG00000156515	**-0,54**	7.22041621322333e-16	**-0,43**	5.44184526502941e-10
HK2	ENSG00000159399	**-0,41**	2.78000065516091e-09	**-0,44**	1.23066696173455e-10
HK3	ENSG00000160883	**0,26**	0.000282823401822961	**0,06**	0.383923684604543
HKDC1	ENSG00000156510	**-0,36**	4.25541914830772e-07	**-0,30**	2.05393658114493e-05
HNF1A	ENSG00000135100	**0,70**	1.95754813519648e-29	**0,14**	0.0475722317693501
HNF4A	ENSG00000101076	**-0,39**	1.68266598161732e-08	**-0,14**	0.0470842022213394
IKBKB	ENSG00000104365	**0,00**	0.955333101431861	**-0,29**	6.32388919004456e-05
INS	ENSG00000254647	**0,76**	3.28813327984766e-37	**0,11**	0.143142992730977
INS-IGF2	ENSG00000129965	**0,76**	7.81247683954942e-37	**0,11**	0.145621556236361
INSR	ENSG00000171105	**-0,42**	1.88076124507707e-09	**-0,13**	0.0798184039304975
IRS1	ENSG00000169047	**0,19**	0.00779396677946712	**-0,36**	2.89957043440865e-07
IRS2	ENSG00000185950	**0,26**	0.000226329855985828	**0,23**	0.00164737210953704
ISL1	ENSG00000016082	**0,42**	1.31545971999211e-09	**0,61**	9.49887396196788e-21
KCNJ11	ENSG00000187486	**0,86**	2.54594666859907e-58	**0,55**	1.3208698915733e-16
MAFA	ENSG00000182759	**1,00**	0	**0,45**	1.05379019767395e-10
MAPK1	ENSG00000100030	**-0,60**	2.99200936810869e-20	**0,08**	0.251282465884628
MAPK10	ENSG00000109339	**0,00**	0.962978066789964	**0,63**	1.24976684735071e-22
MAPK3	ENSG00000102882	**0,36**	2.87917061872028e-07	**0,30**	2.0009180219269e-05
MAPK8	ENSG00000107643	**-0,56**	2.23804290203971e-17	**-0,30**	1.96903338705634e-05
MAPK9	ENSG00000050748	**-0,46**	1.64214884535091e-11	**0,20**	0.00516805948761377
MTOR	ENSG00000198793	**-0,46**	1.39801399115379e-11	**0,03**	0.700528947537056
NEUROD1	ENSG00000162992	**0,23**	0.00134494789150507	**0,79**	3.00215374064025e-41
PDX1	ENSG00000139515	**0,55**	1.23217107593688e-16	**0,77**	8.16286586695198e-39
PHKA2	ENSG00000044446	**0,28**	6.63900708979025e-05	**0,15**	0.0373743719227656
PIK3C2A	ENSG00000011405	**-0,50**	2.01148983364103e-13	**0,03**	0.678424233701642
PIK3CA	ENSG00000121879	**-0,67**	2.39529234700326e-26	**-0,17**	0.0210010819676031
PIK3CB	ENSG00000051382	**-0,63**	4.05405996223875e-22	**-0,20**	0.00456660588519594
PIK3CD	ENSG00000171608	**0,29**	5.85616427793295e-05	**-0,34**	1.86148637990212e-06
PIK3CG	ENSG00000105851	**-0,23**	0.00110467851680458	**0,09**	0.192800970725085
PIK3R1	ENSG00000145675	**-0,70**	4.94075866857068e-29	**-0,09**	0.19404408185396
PIK3R2	ENSG00000105647	**0,77**	6.52792398222713e-39	**0,42**	2.10515977066689e-09
PIK3R3	ENSG00000117461	**-0,05**	0.526593151012579	**0,55**	1.10202969066579e-16
PIK3R5	ENSG00000141506	**-0,26**	0.00030486410290903	**-0,22**	0.00229483366082675
PKM	ENSG00000067225	**-0,52**	8.07020188765281e-15	**-0,03**	0.718055024131205
PRKCD	ENSG00000163932	**-0,35**	8.07477316843992e-07	**-0,18**	0.0106597716713838
PRKCE	ENSG00000171132	**-0,02**	0.807943569172946	**0,52**	2.2305918745835e-14
PRKCZ	ENSG00000067606	**0,22**	0.00276199404735867	**0,11**	0.116537453902725
SLC2A2	ENSG00000163581	**-0,14**	0.0494927316336594	**0,41**	6.18209621481966e-09

SLC2A4	ENSG00000181856	**-0,08**	0.25024436762474	**-0,23**	0.00116038375283762
SOCS1	ENSG00000185338	**0,62**	1.13362876375065e-21	**-0,11**	0.135148843402378
SOCS2	ENSG00000120833	**0,17**	0.0169989833644122	**-0,07**	0.325388859958781
SOCS3	ENSG00000184557	**0,24**	0.000917143429051332	**-0,39**	1.89577019821364e-08
SOCS4	ENSG00000180008	**-0,42**	1.09908953291492e-09	**0,15**	0.0337534242591804
SURF1	ENSG00000148290	**0,15**	0.0402467378878893	**0,04**	0.543655535640052
TNF	ENSG00000232810	**-0,17**	0.0219343961824148	**-0,33**	2.27682620675884e-06
